# Supplementary material for: Optimal tagging strategies for illuminating expression profiles of genes with different abundance in zebrafish
Source: Commun Biol. 2023 Dec 21;6:1300. doi: 10.1038/s42003-023-05686-1 (PMC10739737; doi:10.1038/s42003-023-05686-1)

Supplementary Information

Optimal tagging strategies for illuminating expression profiles of genes with different abundance in zebrafish

Jiannan Liu[1], Wenyuan Li[1], Xuepu Jin[1], Fanjia Lin[1], Jiahuai Han[1,2,3,*], Yingying Zhang[1,*]

*Correspondence authors. Email: J.H. (jhan@xmu.edu.cn), Y.Z. (y.zhang@xmu.edu.cn).

This PDF file includes: Supplementary Figure 1-29

Supplementary Figure 1

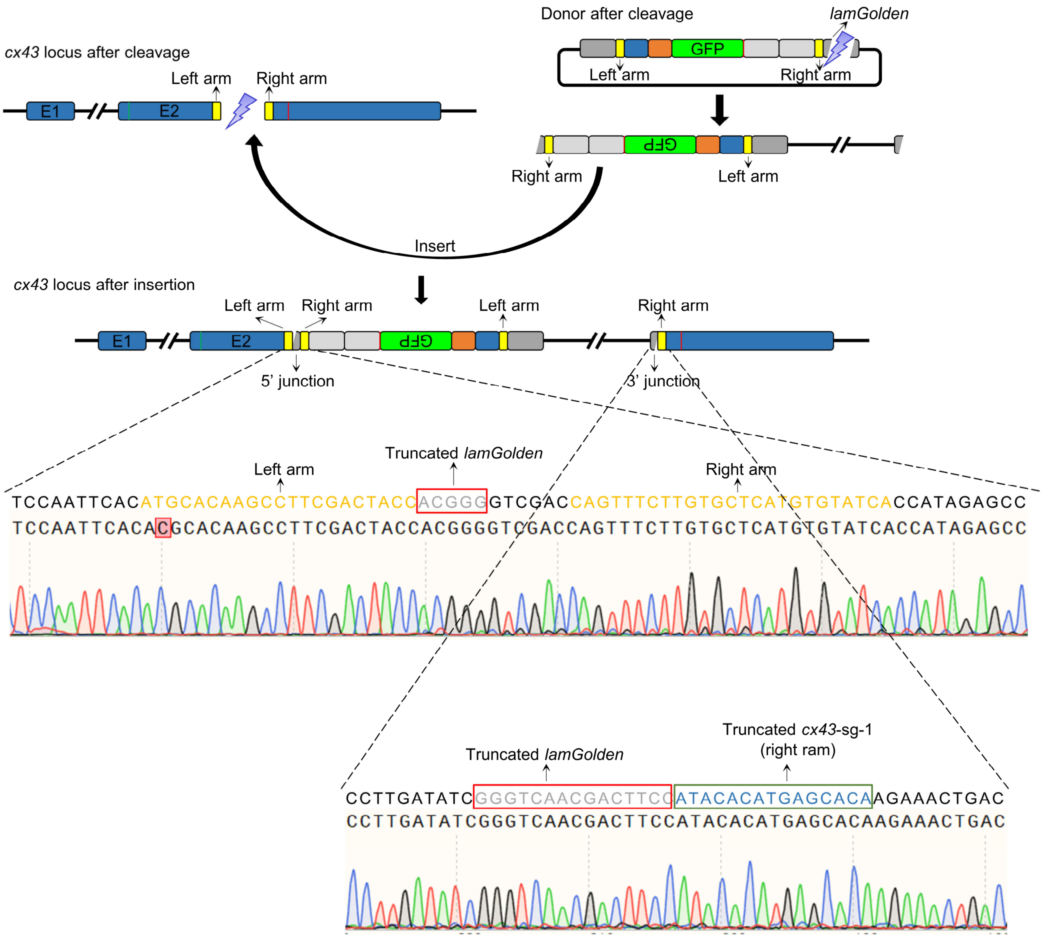

Supplementary Fig. 1. Two artificial Cas9/sgRNA sites in KI donors increase errors in MMEJ-mediated repair.

Incorrect MMEJ-repair caused by two artificial Cas9/sgRNA sites in the donor as exemplified by KI at the *cx43* locus. Cas9 cleaved only the *lamGolden* site adjacent to the right homologous arm and the entire donor was reversely inserted into the genome by NHEJ-mediated repair as revealed by junction PCR and sequencing analysis.

Supplementary Figure 2

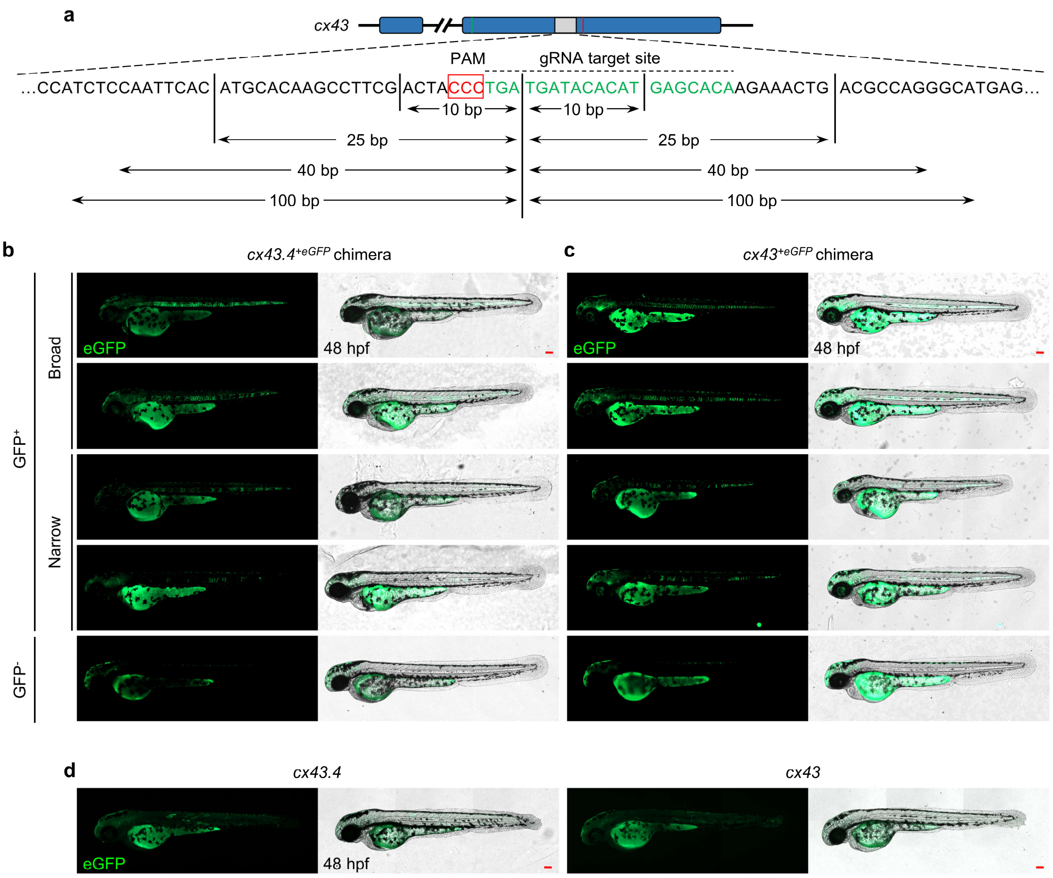

Supplementary Fig. 2. S-25 is an optimized donor for MMEJ-mediated KI. **(a)** Taking *cx43* as an example, mircohomologous right and left arms are sequences downstream and upstream of the double-strand break (DSB), respectively. 10 bp, 25 bp, 40 bp, and 100 bp mircohomologous arms were tested. **(b and c)** Images of microinjected zebrafish embryos using the MMEJ-single strategy at the *cx43.4* or *cx43* locus. GFP signals were observed in the notochord of *cx43.4* or *cx43* F_0 at 48 hpf. Embryos were separated based on the range of GFP expression, broad or narrow. Scale bars, 100 μm. Images are representatives of at least 100 F_0. **(d)** Images of microinjected zebrafish embryos using the MMEJ-double strategy at the *cx43.4* or *cx43* locus. GFP signals were randomly detected in the muscle in some F_0 embryos at 48 hpf. Scale bars, 100 μm. Images are representatives of at least 30 F_0.

Supplementary Figure 3

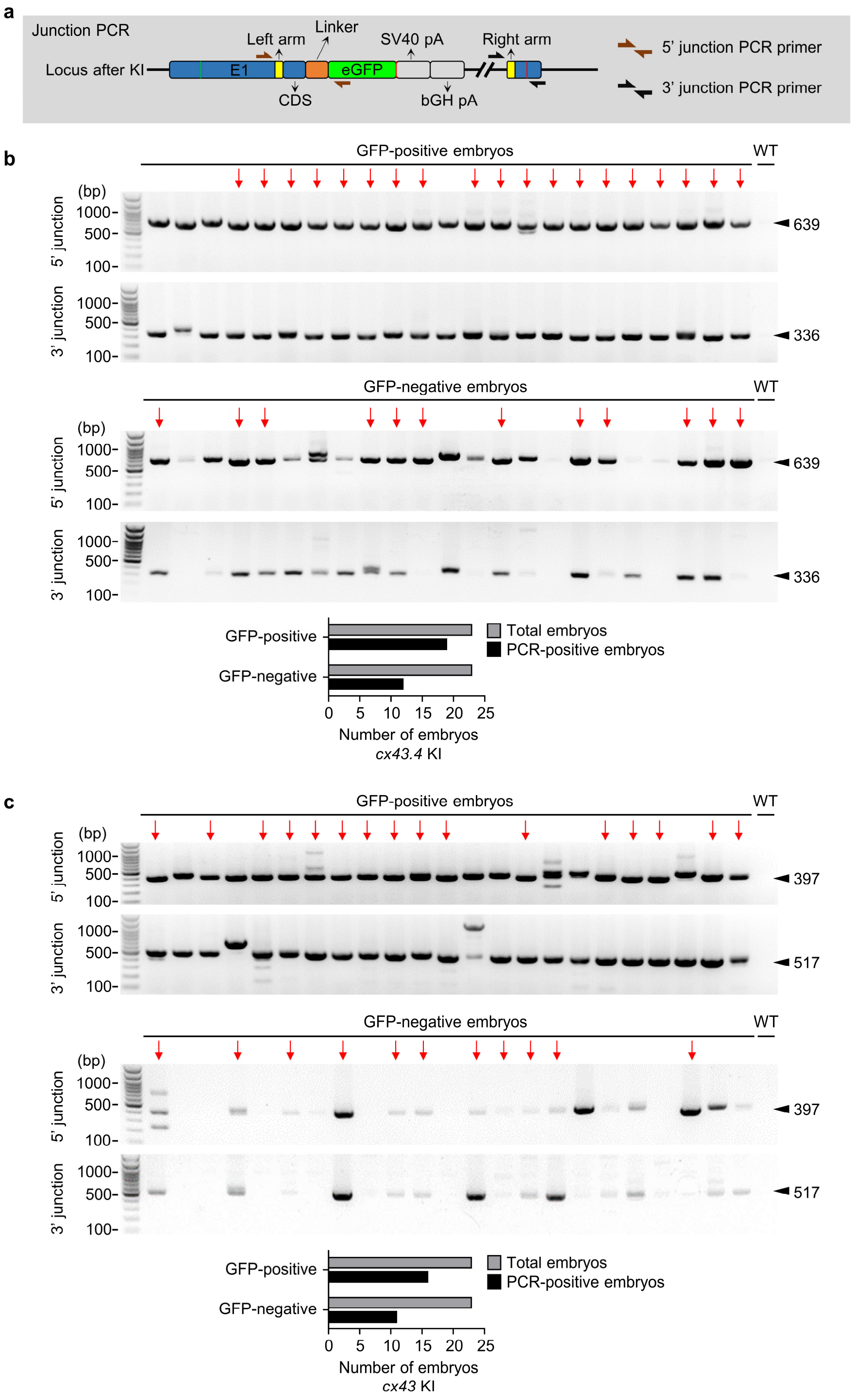

Supplementary Fig. 3. Junction PCR of GFP-positive or GFP-negative embryos after microinjection.

(a) A schematic diagram of the 5'-junction PCR and 3'-junction PCR analysis for the S-25 KI strategy. For the 5'-junction PCR, the forward primer was

designed in the genome upstream of the left arm, and the reverse primer was located in the GFP sequence of the donor. For the 3'-junction PCR, the forward primer was designed in the donor, and the reverse primer was located downstream of the right arm. **(b and c)** Junction PCR analysis of GFP-positive or GFP-negative embryos for *cx43.4* (b) or *cx43* (c) KI. Embryos after microinjection were classified into two groups, GFP-positive and GFP-negative groups at 48 hpf. Twenty-three microinjected embryos in each group were randomly selected and genotyped. PCR-positive F_0 embryos were indicated by red arrowheads.

Supplementary Figure 4

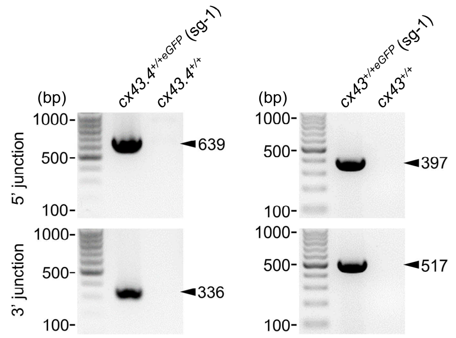

Supplementary Fig. 4. Junction PCR analysis of *cx43.4*$^{+/+eGFP}$ and *cx43*$^{+/+eGFP}$ F_1.

F_1 embryos carrying GFP-labeled *cx43.4* or *cx43* alleles were identified by 5'- and 3'-junction PCR. Data are representatives of at least 10 F_1.

Supplementary Figure 5

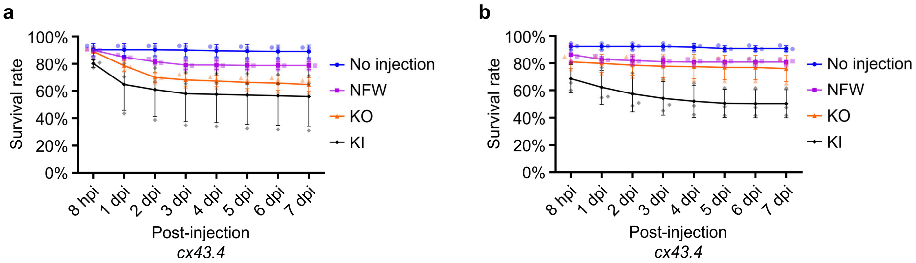

Supplementary Fig. 5. Effects of the S-25-based KI on survival rates of the microinjected embryos.

Survival rates of embryos microinjected with different gene-editing materials for *cx43.4* (a) or *cx43* (b). Different materials, including NFW (nuclease-free water), KO mix (Cas9 mRNA and sgRNA), and KI mix (Cas9 mRNA, two sgRNAs, and the corresponding S-25 donor), were injected into at least 100 one-cell-stage WT embryos. Survival rates were analyzed at different time points. Non-injected embryos were included as the control. Data represent mean ± SD of 3 independent experiments. hpi, hours post-injection; dpi, days post-injection.

Supplementary Figure 6

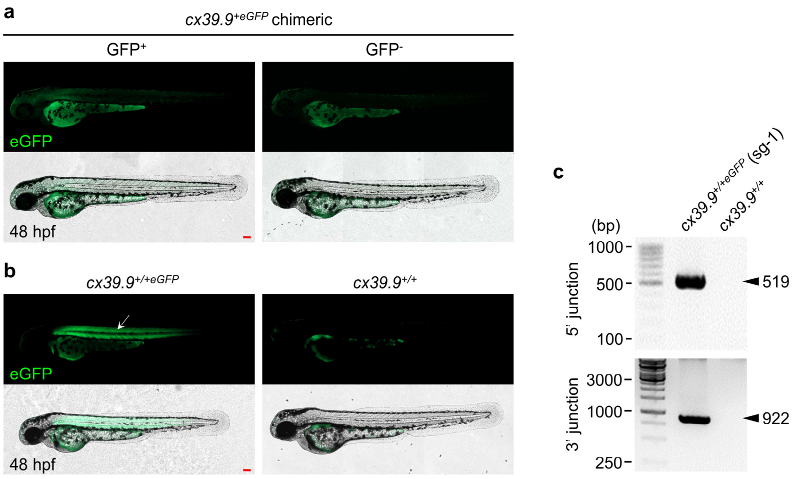

Supplementary Fig. 6. Generation of the GFP-labeled *cx39.9* KI zebrafish.

(a and b) Images of microinjected F_0 (a) and F_1 (b) embryos for *cx39.9* KI by using the S-25 strategy at 48 hpf. Scale bars, 100 μm. Images are representatives of at least 7 embryos. **(c)** Junction PCR analysis of the *cx39.9*$^{+/+eGFP}$ F_1 zebrafish. Data are representatives of at least 10 F_1.

Supplementary Figure 7

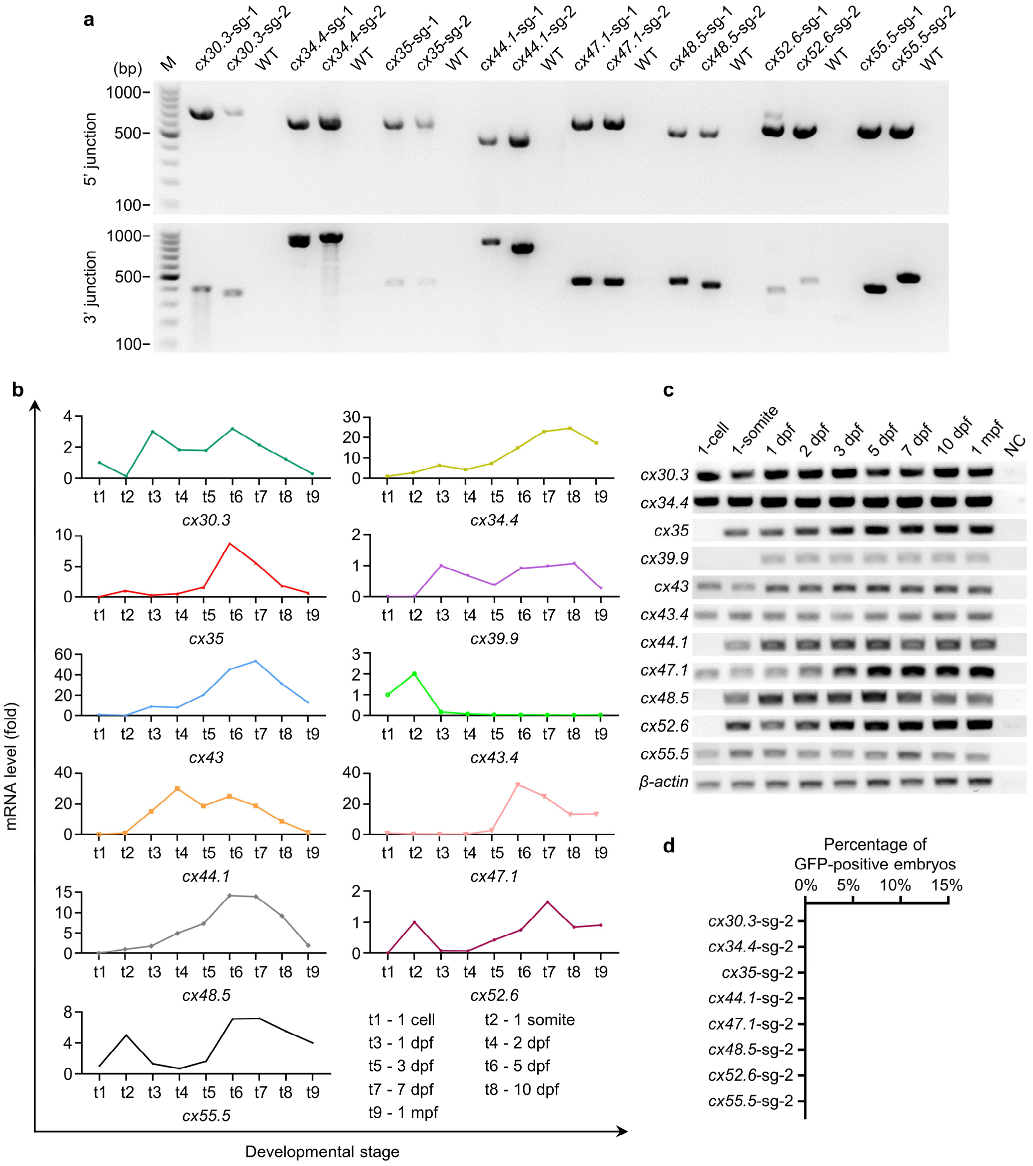

Supplementary Fig. 7. Transcriptional analysis of *connexins*.

(a) Junction PCR analysis of microinjected F_0 embryos. Two high-efficiency sgRNAs were chosen for each gene. For each sgRNA, 5 microinjected embryos were randomly selected and pooled for genomic DNA extraction and junction PCR analysis at 24 hpf. WT embryos were used as negative controls. **(b and c)** Real-time quantitative PCR (RT-qPCR) analysis of *connexins* at different developmental stages of zebrafish. Thirty WT zebrafish embryos were pooled to extract RNA for time points before 1-month post fertilization (mpf). At 1 mpf, a whole WT zebrafish was frozen to death and treated with 1 mL of RNAIso

Plus reagent for total RNA extraction and the subsequent RT-qPCR. For a specific gene in each sample, expression of the target gene was normalized to that of *β-actin*, and then the threshold cycle ($2^{-\Delta\Delta Ct}$) method was used and the fold change of the target gene in each sample relative to the time point the expression was first detected was plotted (b). qPCR products were verified by electrophoresis (c). NC, negative control. **(d)** Percentages of GFP-positive F_0 embryos after tagging *cx30.3*, *cx34.4*, *cx35*, *cx44.1*, *cx47.1*, *cx48.5*, *cx52.6*, or *cx55.5*. S-25 strategy was performed on at least 100 WT embryos using an alternative high-efficiency sgRNA for each gene and GFP-positive F_0 embryos were counted at 48 hpf.

Supplementary Figure 8

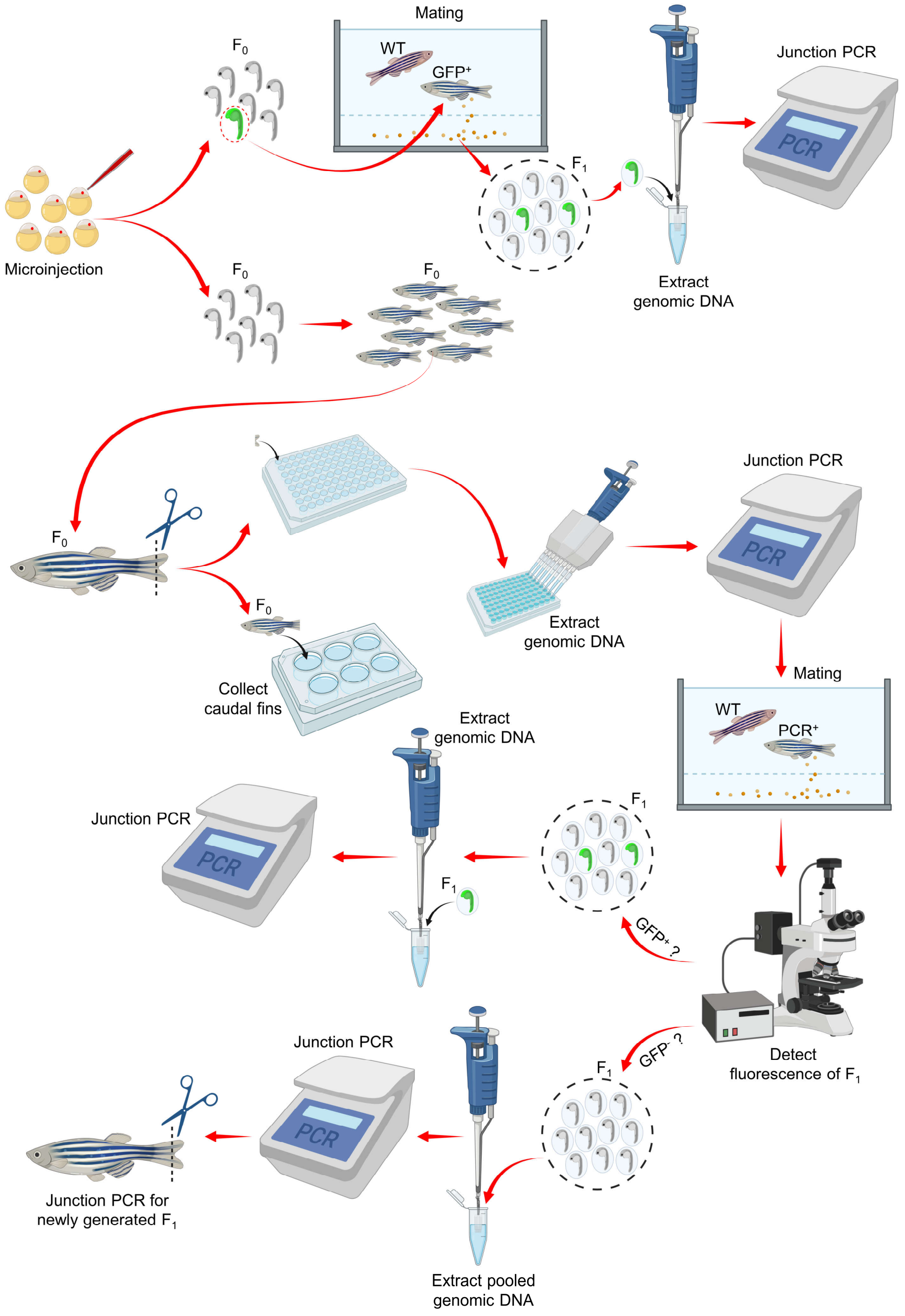

Supplementary Fig. 8. A schematic diagram of a germline transmission screen procedure combining fluorescence examination and junction PCR.

If GFP-positive embryos are observed in microinjected F_0 zebrafish, all of the GFP-positive F_0 embryos are raised to adulthood and outbred with WT. The F_1 embryos are identified by GFP expression patterns and genotyping. However,

if GFP signals cannot be detected in F_0 embryos after microinjection, all of the F_0 embryos are raised to 1 month old. Genomic DNA is then isolated from the caudal fins of these 1-month-old F_0 zebrafish and used for junction PCR to identify PCR-positive F_0. Next, fluorescence is examined in F_1 embryos obtained from outbreeding PCR-positive F_0 with WT zebrafish. If fluorescence is detected in F_1 embryos, junction PCR would be performed to determine the genotype of the F_1. Otherwise, all of the GFP-negative F_1 embryos are pooled for genomic DNA extraction and junction PCR analysis. If KI is detected in the pooled DNA, the corresponding F_0 adults would be outbred with WT again to generate new F_1. These F_1 are genotyped using caudal fins at 1-month-old to confirm KI.

Supplementary Figure 9

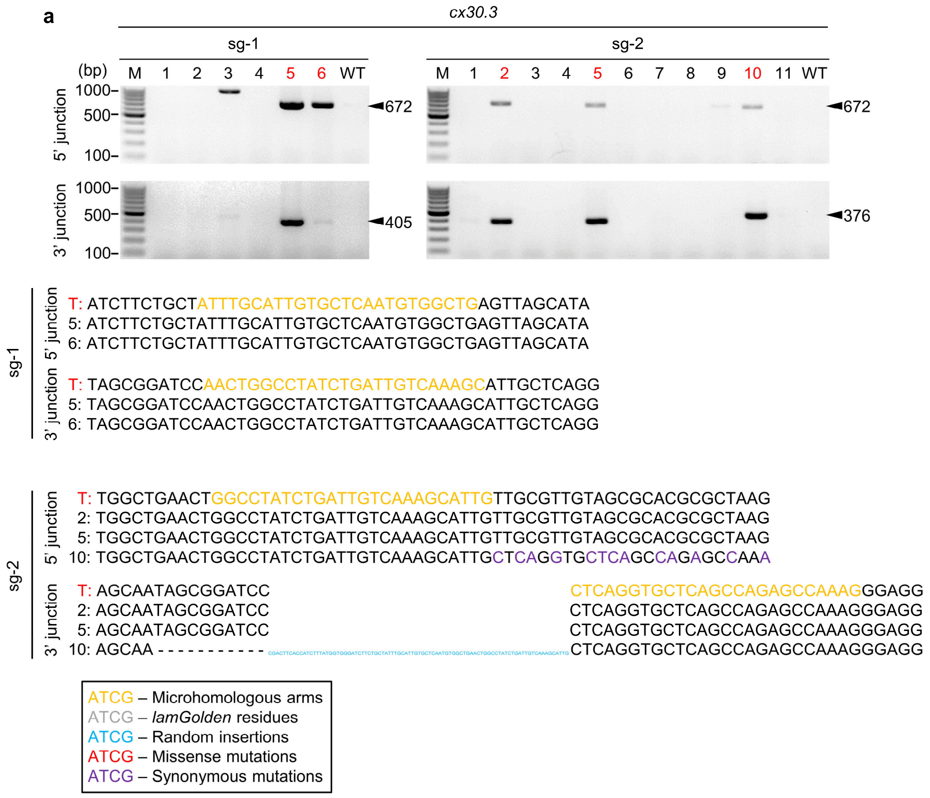

b

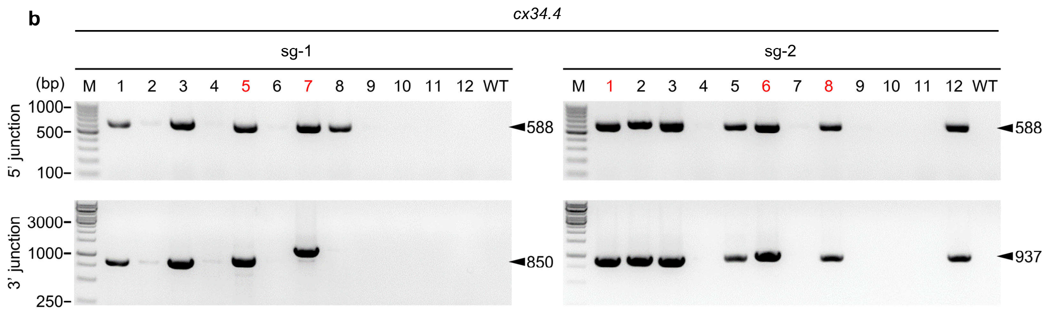

sg-1

5' junction

T: ACTGGCAAGC GAAGACCAGCCGGCACCAGCATACAGCGTAGTTAT
1: ACTGGCAAGC GAAGACCAGCCGGCACCAGCATACAGCGTAGTTAT
3: ACTGGCAAGC GTAGACCAGCCGGCACCAGCATACAGCGTAGTTAT
5: ACTGGCAAGC GAAGACCAGCCGGCACCAGCATACAGCGTAGTTAT
7: ACTGGCAAGC GAAGACCAGCCGGCACCAGCATACAGCGTAGTTAT

3' junction

T: TAGCGGATCCGTGTGGTCATGTCAGCCAAAAAGAA AACCA
1: TAGCGGATCCGTGTGGTCATGTCAGCCAAAAAGAA AACCA
3: TAGCGGATCCGTGTGGTCATGTCAGCCAAAAAGAA AACCA
5: TAGCGGATCCGTGTGGTCATGTCAGCCAAAAAGAA AACCA
7: TAGCGGATCCGTGTGGTCATGTCAGCCAAAAAGAA AACCA

Donor fragment (*cat*-promotor, *Amp*)

sg-2

5' junction

T: GGTGCAGAGC TCACGACATGTGAACAGAGAGAGATCTATGGCCAAC
1: GGTGCAGAGC TCACGACATGTGAACAGAGAGAGATCTATGGCCAAC
2: GGTGCAGAGC TCACGACATGTGAACAGAGAGAGAGAGATGGCCAAC
3: Multi-peaks in left microhomologous arm
5: GGTGCAGAGC TCACGACATGTGAACAGAGAGAGATCTATGGCCAAC
6: GGTGCAGAGC TCACGACATGTGAACAGAGAGAGATCTATGGCCAAC
8: GGTGCAGAGC TCACGACATGTGAACAGAGAGAGATCTATGGCCAAC
12: Multi-peaks in left microhomologous arm

3' junction

T: TAGCGGATCC CCATGGCTAATATGACAAACTTGAATGCTCATTTA
1: TAGCGGATCC CCATGGCTAATATGACAAACTTGAATGCTCATTTA
2: TAGCGGATCC CCATGGCTAATATGACAAACTTGAATGCTCATTTA
3: TAGCGGATCC CCATGGCTAATATGACAAACTTGAATGCTCATTTA
5: Multi-peaks in right microhomologous arm
6: TAGCGGATCC - - ATGGCTAATATGACAAACTTGAATGCTCATTTA
8: TAGCGGATCC CCATGGCTAATATGACAAACTTGAATGCTCATTTA
12: TAGCGGATCC CCATGGCTAATATGACAAACTTGAATGCTCATTTA

cx34.4 genome fragment

c

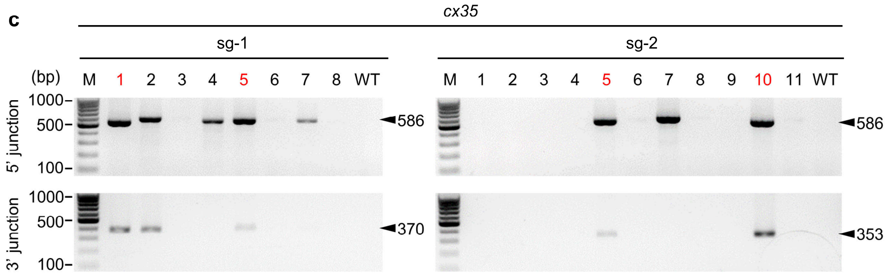

sg-1

5' junction

T: AGAAACAAGG ACTTACCGCGGATGAGCATGCCGAACTTTGGGCGG
1: AGAAACAAGG ACTTACCGCGGATGAGCATGCCGAACTTTGGGCGG
2: AGAAACAAGG ACTTACCGCGGATGAGCATGCCGAACTTTGGGCGG
5: AGAAACAAGG ACTTACCGCGGATGAGCATGCCGAACTTTGGGCGG

3' junction

T: TAGCGGATCCTTTCGGAAGAACCCAGTCCAGTGACTCTGCCTACG
1: TAGCGGATCCTTTCGGAAGAACCCAGTCCAGTGACTCTGCCTACG
2: TAGCGGATCCTTTCGGAAGAACCCAGTCCAGTGACTCTGCCTACG
5: TAGCGGATCCTTTCGGAAGAACCCAGTCCAGTGACTCTGCCTACG

sg-2

5' junction

T: GCGGATGAGCATGCCGAATTTCGGAAGAACCCAGTCTTCAGATAG
5: GCGGATGAGCATGCCGAATTTCGGAAGAACCCAGTCTTCAGATAG
10: GCGGATGAGCATGCCGAATTTCGGAAGAACCCAGTCTTCAGATAG

3' junction

T: TAGCGGATCCCCAGTGACTCTGCCTACGTTTAATGCTTTCTTCGT
5: TAGCGGATCCCCAGTGACTCTGCCTACGTTTAATGCTTTCTTCGT
10: TAGCGGATCCCCAGTGACTCTGCCTACGTTTAATGCTTTCTTCGT

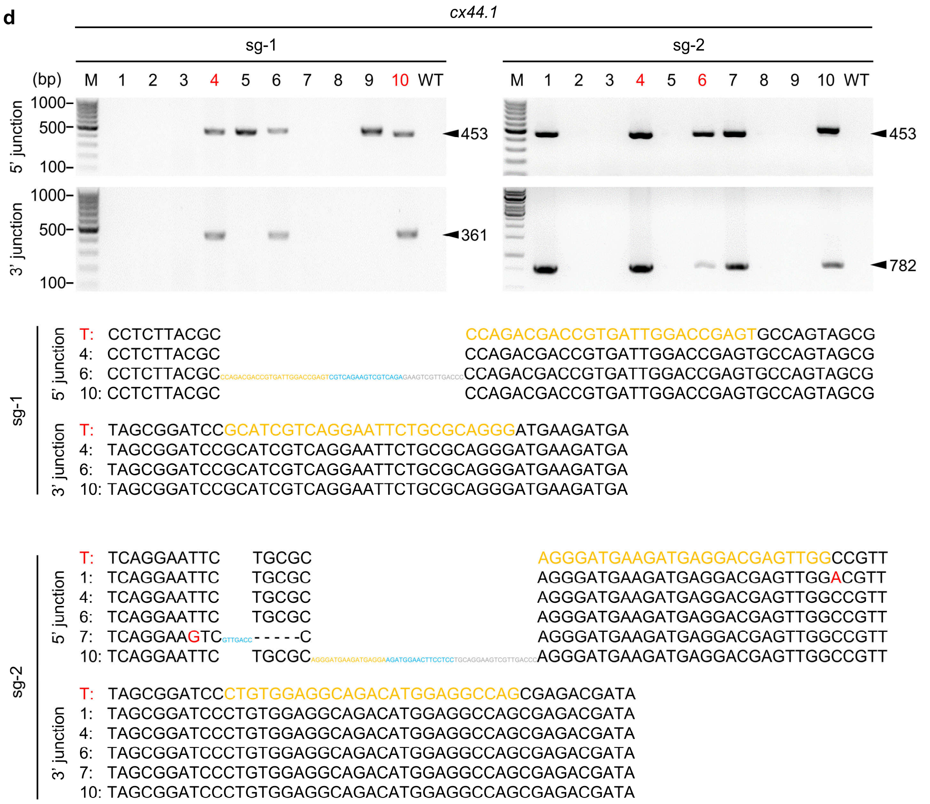
d
cx44.1
sg-1
sg-2
(bp)
M 1 2 3 4 5 6 7 8 9 10 WT
M 1 2 3 4 5 6 7 8 9 10 WT
1000–
500–
100–
5' junction
3' junction
453
453
361
782
sg-1
5' junction
T: CCTCTTACGC CCAGACGACCGTGATTGGACCGAGTGCCAGTAGCG
4: CCTCTTACGC CCAGACGACCGTGATTGGACCGAGTGCCAGTAGCG
6: CCTCTTACGC CCAGACGACCGTGATTGGACCGAGTGCCAGTAGCG
10: CCTCTTACGC CCAGACGACCGTGATTGGACCGAGTGCCAGTAGCG
3' junction
T: TAGCGGATCCGCATCGTCAGGAATTCTGCGCAGGGATGAAGATGA
4: TAGCGGATCCGCATCGTCAGGAATTCTGCGCAGGGATGAAGATGA
6: TAGCGGATCCGCATCGTCAGGAATTCTGCGCAGGGATGAAGATGA
10: TAGCGGATCCGCATCGTCAGGAATTCTGCGCAGGGATGAAGATGA
sg-2
5' junction
T: TCAGGAATTC TGCGC AGGGATGAAGATGAGGACGAGTTGGCCGTT
1: TCAGGAATTC TGCGC AGGGATGAAGATGAGGACGAGTTGGACGTT
4: TCAGGAATTC TGCGC AGGGATGAAGATGAGGACGAGTTGGCCGTT
6: TCAGGAATTC TGCGC AGGGATGAAGATGAGGACGAGTTGGCCGTT
7: TCAGGAAGTC-----C AGGGATGAAGATGAGGACGAGTTGGCCGTT
10: TCAGGAATTC TGCGC AGGGATGAAGATGAGGACGAGTTGGCCGTT
3' junction
T: TAGCGGATCCCTGTGGAGGCAGACATGGAGGCCAGCGAGACGATA
1: TAGCGGATCCCTGTGGAGGCAGACATGGAGGCCAGCGAGACGATA
4: TAGCGGATCCCTGTGGAGGCAGACATGGAGGCCAGCGAGACGATA
6: TAGCGGATCCCTGTGGAGGCAGACATGGAGGCCAGCGAGACGATA
7: TAGCGGATCCCTGTGGAGGCAGACATGGAGGCCAGCGAGACGATA
10: TAGCGGATCCCTGTGGAGGCAGACATGGAGGCCAGCGAGACGATA

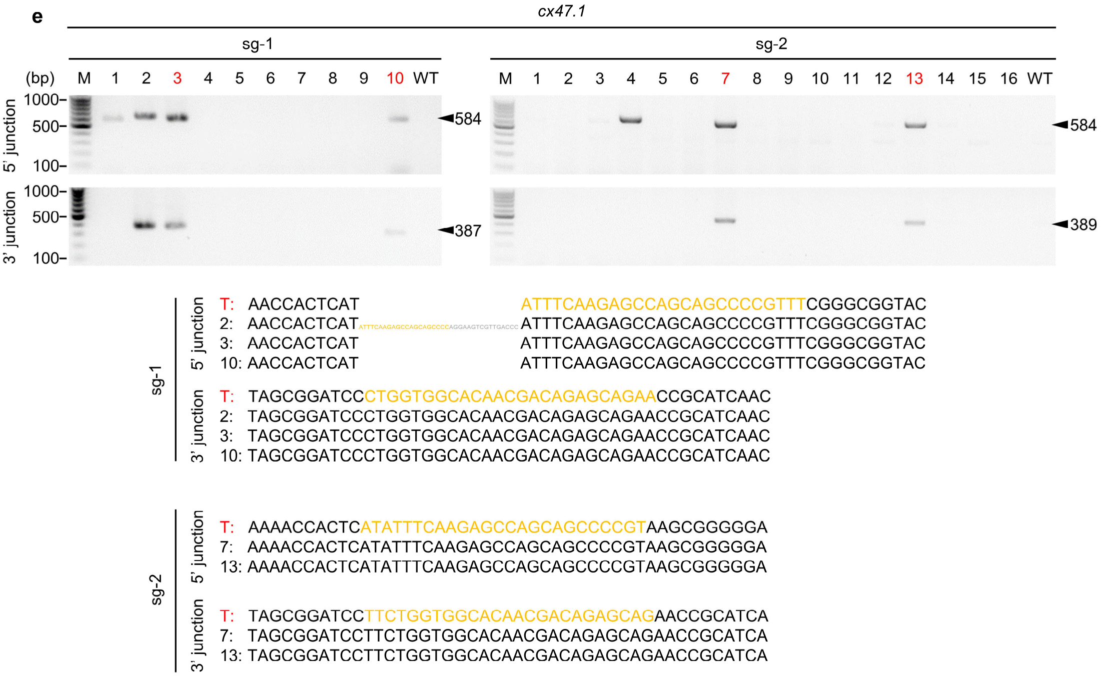
e
cx47.1
sg-1
sg-2
(bp)
M 1 2 3 4 5 6 7 8 9 10 WT
M 1 2 3 4 5 6 7 8 9 10 11 12 13 14 15 16 WT
1000–
500–
100–
5' junction
3' junction
584
584
387
389
sg-1
5' junction
T: AACCACTCAT ATTTCAAGAGCCAGCAGCCCCGTTTCGGGCGGTAC
2: AACCACTCAT ATTTCAAGAGCCAGCAGCCCCGTTTCGGGCGGTAC
3: AACCACTCAT ATTTCAAGAGCCAGCAGCCCCGTTTCGGGCGGTAC
10: AACCACTCAT ATTTCAAGAGCCAGCAGCCCCGTTTCGGGCGGTAC
3' junction
T: TAGCGGATCCCTGGTGGCACAACGACAGAGCAGAACCGCATCAAC
2: TAGCGGATCCCTGGTGGCACAACGACAGAGCAGAACCGCATCAAC
3: TAGCGGATCCCTGGTGGCACAACGACAGAGCAGAACCGCATCAAC
10: TAGCGGATCCCTGGTGGCACAACGACAGAGCAGAACCGCATCAAC
sg-2
5' junction
T: AAAACCACTCATATTTCAAGAGCCAGCAGCCCCGTAAGCGGGGGA
7: AAAACCACTCATATTTCAAGAGCCAGCAGCCCCGTAAGCGGGGGA
13: AAAACCACTCATATTTCAAGAGCCAGCAGCCCCGTAAGCGGGGGA
3' junction
T: TAGCGGATCCTTCTGGTGGCACAACGACAGAGCAGAACCGCATCA
7: TAGCGGATCCTTCTGGTGGCACAACGACAGAGCAGAACCGCATCA
13: TAGCGGATCCTTCTGGTGGCACAACGACAGAGCAGAACCGCATCA

f

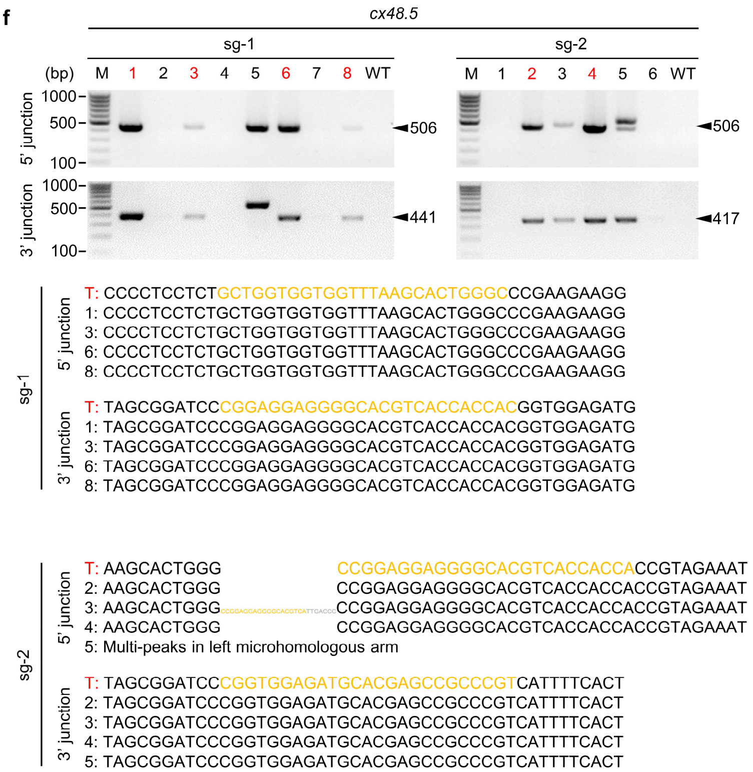
cx48.5
sg-1
sg-2
(bp)
M 1 2 3 4 5 6 7 8 WT
M 1 2 3 4 5 6 WT
5' junction
3' junction
1000
500
100
506
441
417
T: CCCCTCCTCTGCTGGTGGTGGTTTAAGCACTGGGCCCGAAGAAGG
1: CCCCTCCTCTGCTGGTGGTGGTTTAAGCACTGGGCCCGAAGAAGG
3: CCCCTCCTCTGCTGGTGGTGGTTTAAGCACTGGGCCCGAAGAAGG
6: CCCCTCCTCTGCTGGTGGTGGTTTAAGCACTGGGCCCGAAGAAGG
8: CCCCTCCTCTGCTGGTGGTGGTTTAAGCACTGGGCCCGAAGAAGG
T: TAGCGGATCCCGGAGGAGGGGCACGTCACCACCACGGTGGAGATG
1: TAGCGGATCCCGGAGGAGGGGCACGTCACCACCACGGTGGAGATG
3: TAGCGGATCCCGGAGGAGGGGCACGTCACCACCACGGTGGAGATG
6: TAGCGGATCCCGGAGGAGGGGCACGTCACCACCACGGTGGAGATG
8: TAGCGGATCCCGGAGGAGGGGCACGTCACCACCACGGTGGAGATG
T: AAGCACTGGG CCGGAGGAGGGGCACGTCACCACCACCGTAGAAAT
2: AAGCACTGGG CCGGAGGAGGGGCACGTCACCACCACCGTAGAAAT
3: AAGCACTGGG CCGGAGGAGGGGCACGTCACCACCACCGTAGAAAT
4: AAGCACTGGG CCGGAGGAGGGGCACGTCACCACCACCGTAGAAAT
5: Multi-peaks in left microhomologous arm
T: TAGCGGATCCCGGTGGAGATGCACGAGCCGCCCGTCATTTTCACT
2: TAGCGGATCCCGGTGGAGATGCACGAGCCGCCCGTCATTTTCACT
3: TAGCGGATCCCGGTGGAGATGCACGAGCCGCCCGTCATTTTCACT
4: TAGCGGATCCCGGTGGAGATGCACGAGCCGCCCGTCATTTTCACT
5: TAGCGGATCCCGGTGGAGATGCACGAGCCGCCCGTCATTTTCACT

g

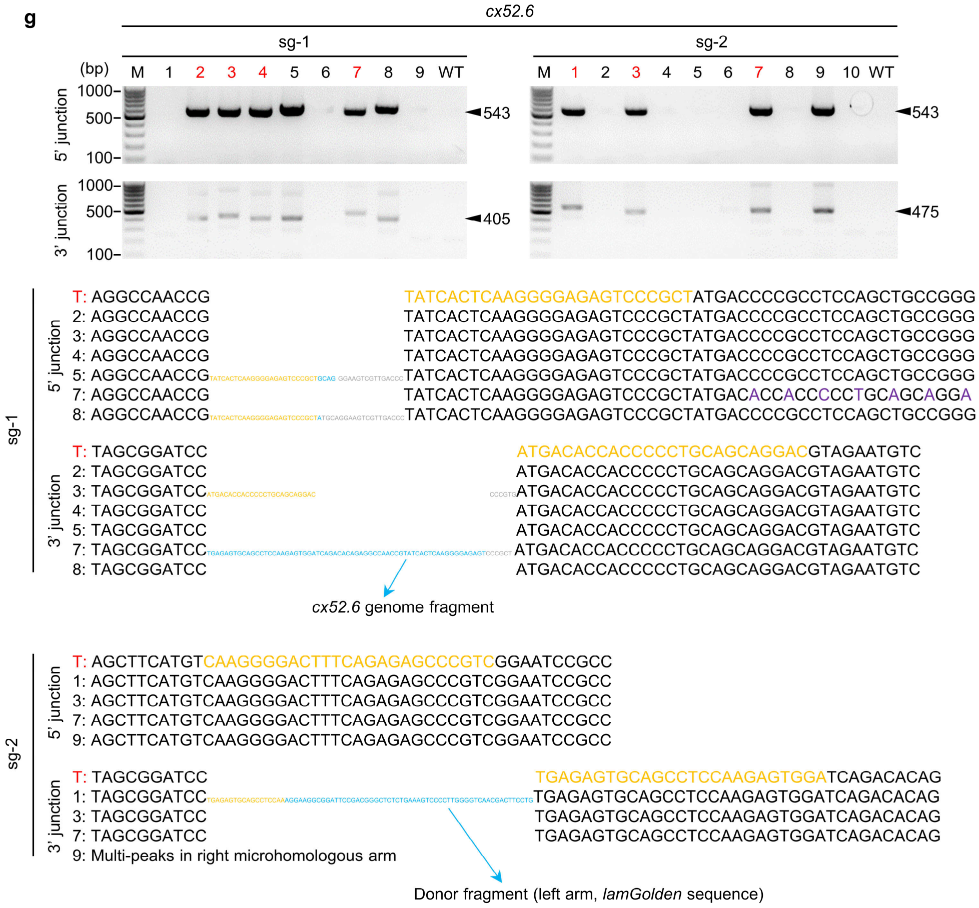
cx52.6
sg-1
sg-2
(bp)
M 1 2 3 4 5 6 7 8 9 WT
M 1 2 3 4 5 6 7 8 9 10 WT
5' junction
3' junction
1000
500
100
543
405
475
T: AGGCCAACCG TATCACTCAAGGGGAGAGTCCCGCTATGACCCCGCCTCCAGCTGCCGGG
2: AGGCCAACCG TATCACTCAAGGGGAGAGTCCCGCTATGACCCCGCCTCCAGCTGCCGGG
3: AGGCCAACCG TATCACTCAAGGGGAGAGTCCCGCTATGACCCCGCCTCCAGCTGCCGGG
4: AGGCCAACCG TATCACTCAAGGGGAGAGTCCCGCTATGACCCCGCCTCCAGCTGCCGGG
5: AGGCCAACCG TATCACTCAAGGGGAGAGTCCCGCTATGACCCCGCCTCCAGCTGCCGGG
7: AGGCCAACCG TATCACTCAAGGGGAGAGTCCCGCTATGACACCACCCCCTGCAGCAGGA
8: AGGCCAACCG TATCACTCAAGGGGAGAGTCCCGCTATGACCCCGCCTCCAGCTGCCGGG
T: TAGCGGATCC ATGACACCACCCCCTGCAGCAGGACGTAGAATGTC
2: TAGCGGATCC ATGACACCACCCCCTGCAGCAGGACGTAGAATGTC
3: TAGCGGATCC ATGACACCACCCCCTGCAGCAGGACGTAGAATGTC
4: TAGCGGATCC ATGACACCACCCCCTGCAGCAGGACGTAGAATGTC
5: TAGCGGATCC ATGACACCACCCCCTGCAGCAGGACGTAGAATGTC
7: TAGCGGATCC ATGACACCACCCCCTGCAGCAGGACGTAGAATGTC
8: TAGCGGATCC ATGACACCACCCCCTGCAGCAGGACGTAGAATGTC
cx52.6 genome fragment
T: AGCTTCATGTCAAGGGGACTTTCAGAGAGCCCGTCGGAATCCGCC
1: AGCTTCATGTCAAGGGGACTTTCAGAGAGCCCGTCGGAATCCGCC
3: AGCTTCATGTCAAGGGGACTTTCAGAGAGCCCGTCGGAATCCGCC
7: AGCTTCATGTCAAGGGGACTTTCAGAGAGCCCGTCGGAATCCGCC
9: AGCTTCATGTCAAGGGGACTTTCAGAGAGCCCGTCGGAATCCGCC
T: TAGCGGATCC TGAGAGTGCAGCCTCCAAGAGTGGATCAGACACAG
1: TAGCGGATCC TGAGAGTGCAGCCTCCAAGAGTGGATCAGACACAG
3: TAGCGGATCC TGAGAGTGCAGCCTCCAAGAGTGGATCAGACACAG
7: TAGCGGATCC TGAGAGTGCAGCCTCCAAGAGTGGATCAGACACAG
9: Multi-peaks in right microhomologous arm
Donor fragment (left arm, lamGolden sequence)

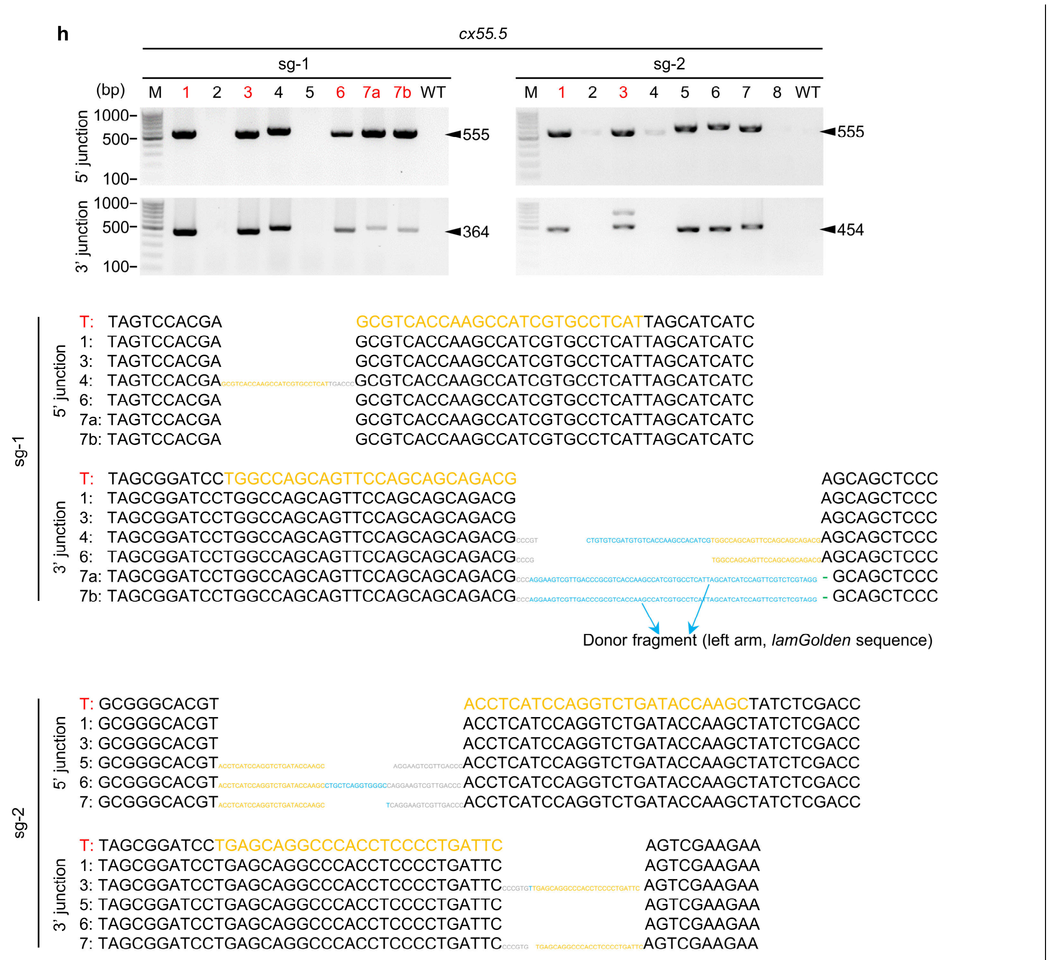

Supplementary Fig. 9. Germline transmission screen results.

Germline transmission screens for GFP-tagged *connexins* including *cx30.3* (a), *cx34.4* (b), *cx35* (c), *cx44.1* (d), *cx47.1* (e), *cx48.5* (f), *cx52.6* (g), and *cx55.5* (h) following procedures introduced in Supplemental Figure 8. GFP was detected in F_1 embryos for *cx30.3*, *cx44.1*, and *cx48.5* KI but not in F_1 embryos for *cx34.4*, *cx35*, *cx47.1*, *cx52.6*, or *cx55.5* KI. Germline transmission was confirmed by junction PCR and sequencing for all tested genes. Numbers on the top of the gels represent F_1 zebrafish pools generated by different F_0 zebrafish. Desired F_1 were indicated by red numbers. T, the desired sequence used as template for the alignment.

Supplementary Figure 10

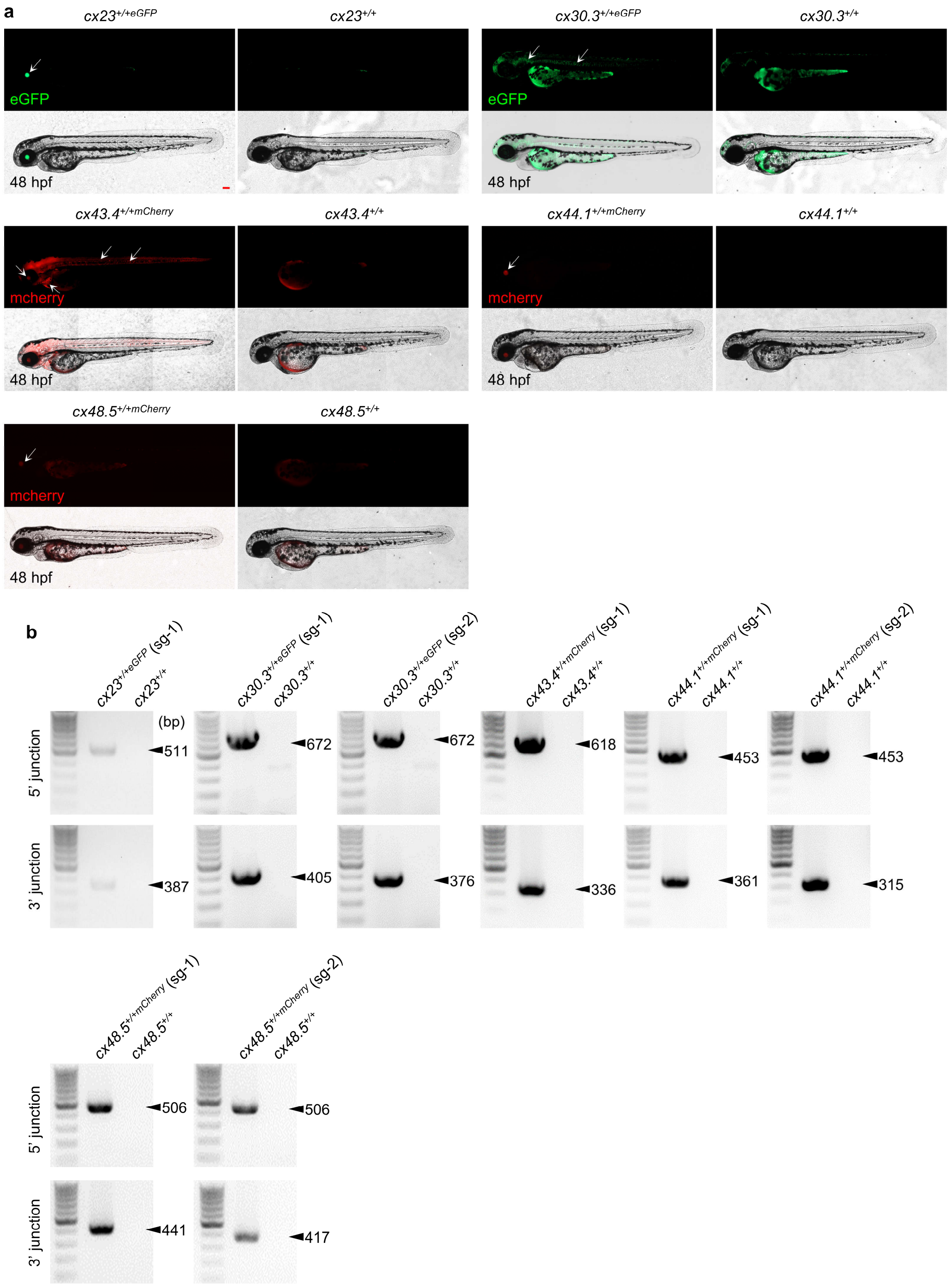

Supplementary Fig. 10. Images and junction PCR analysis of F_1 zebrafish carrying fluorescence-labeled *connexins*.

(a) *cx23*, *cx30.3*, *cx43.4*, *cx44.1*, or *cx48.5* KI F_1 zebrafish with detectable fluorescence at 48 hpf. *cx23*$^{+/+eGFP}$, *cx44.1*$^{+/+mCherry}$, and *cx48.5*$^{+/+mCherry}$ larvae showed fluorescence in the lens. *cx30.3*$^{+/+eGFP}$ larvae showed GFP expression

in the anterior notochord and skin. *cx43.4*$^{+/+mCherry}$ larvae showed the same expression patterns as that of the *cx43.4*$^{+/+eGFP}$ larvae in Fig. 1D, 4D, and 5E. Scale bars, 100 μm. Images are representatives of at least 10 F_1. **(b)** *cx23*$^{+/+eGFP}$, *cx30.3*$^{+/+eGFP}$, *cx43.4*$^{+/+mCherry}$, *cx44.1*$^{+/+mCherry}$, and *cx48.5*$^{+/+mCherry}$ F_1 embryos carrying fluorescence-labeled *connexin* alleles were confirmed by 5'- and 3'-junction PCR. Littermates were used as negative controls. Data are representatives of at least 10 F_1.

Supplementary Figure 11

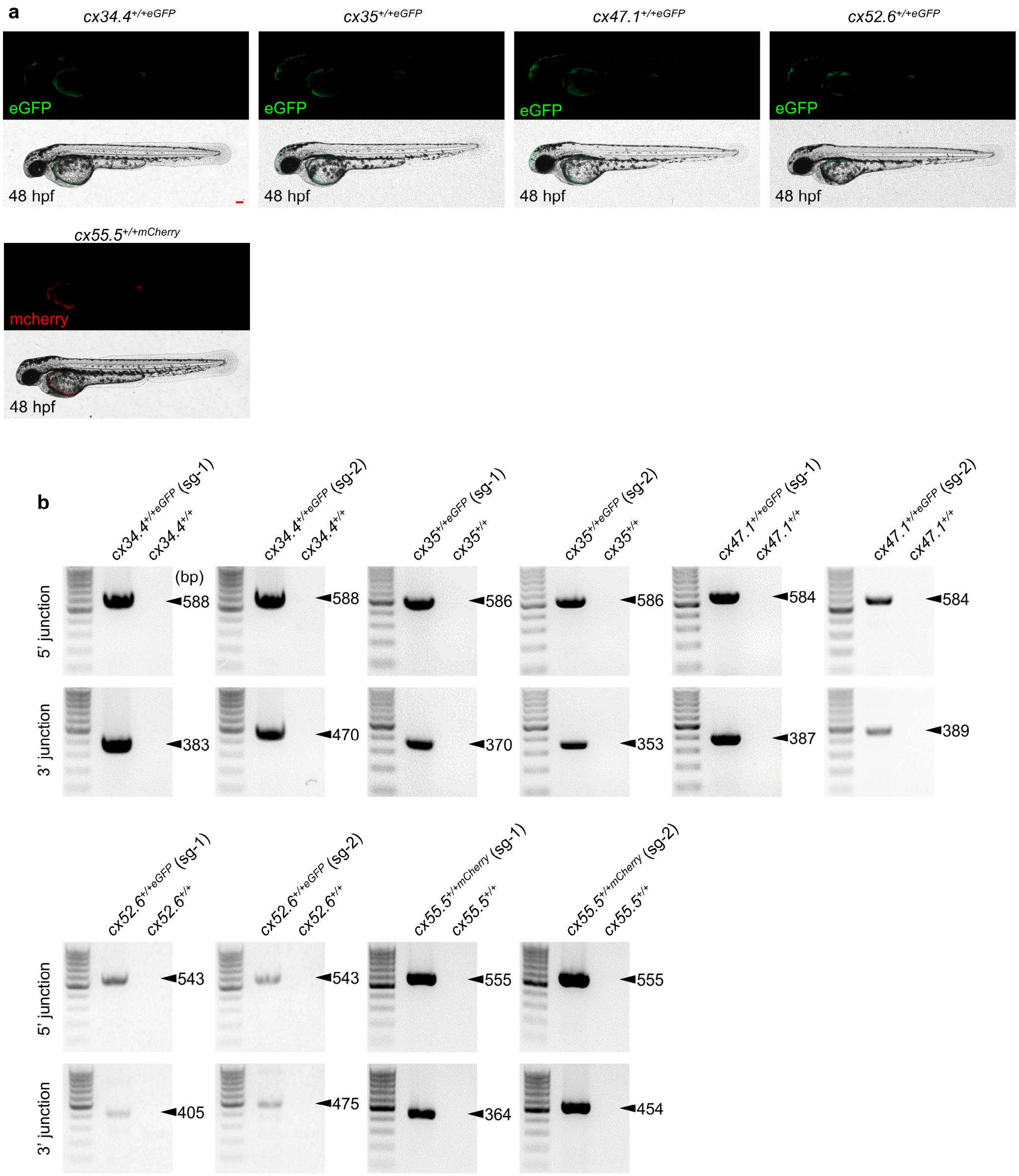

Supplementary Fig. 11. Images and Junction PCR analysis of *cx34.4*, *cx35*, *cx47.1*, *cx52.6*, or *cx55.5* KI F_1 zebrafish.

(a) *cx34.4*$^{+/+eGFP}$, *cx35*$^{+/+eGFP}$, *cx47.1*$^{+/+eGFP}$, *cx52.6*$^{+/+eGFP}$, and *cx55.5*$^{+/+mCherry}$ larvae showed no fluorescence at 48 hpf. Scale bars, 100 μm. Images are representatives of at least 10 F_1. **(b)** *cx34.4*$^{+/+eGFP}$, *cx35*$^{+/+eGFP}$, *cx47.1*$^{+/+eGFP}$, *cx52.6*$^{+/+eGFP}$, and *cx55.5*$^{+/+mCherry}$ F_1 embryos were confirmed by 5'- and 3'-junction PCR. Littermates were included as negative controls. Data are representatives of at least 10 F_1.

Supplementary Figure 12

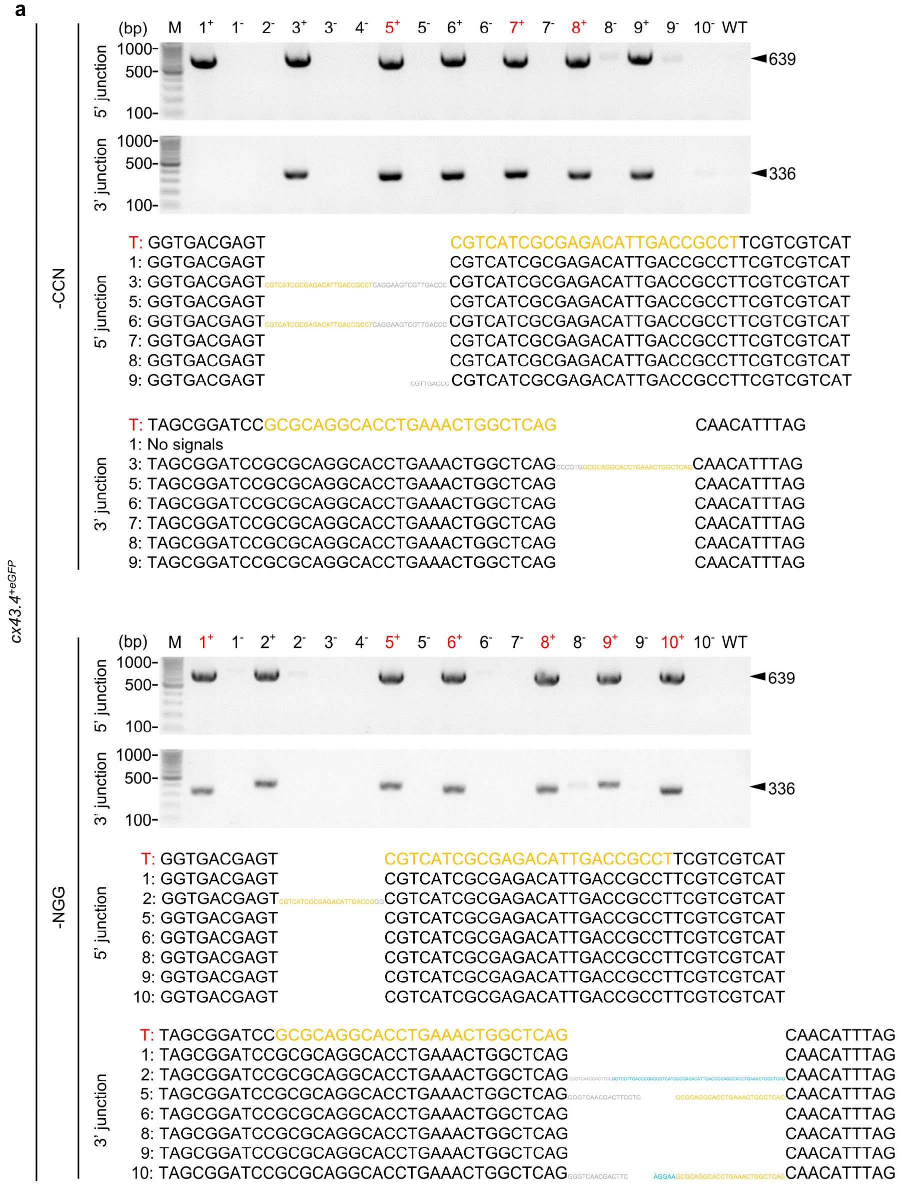

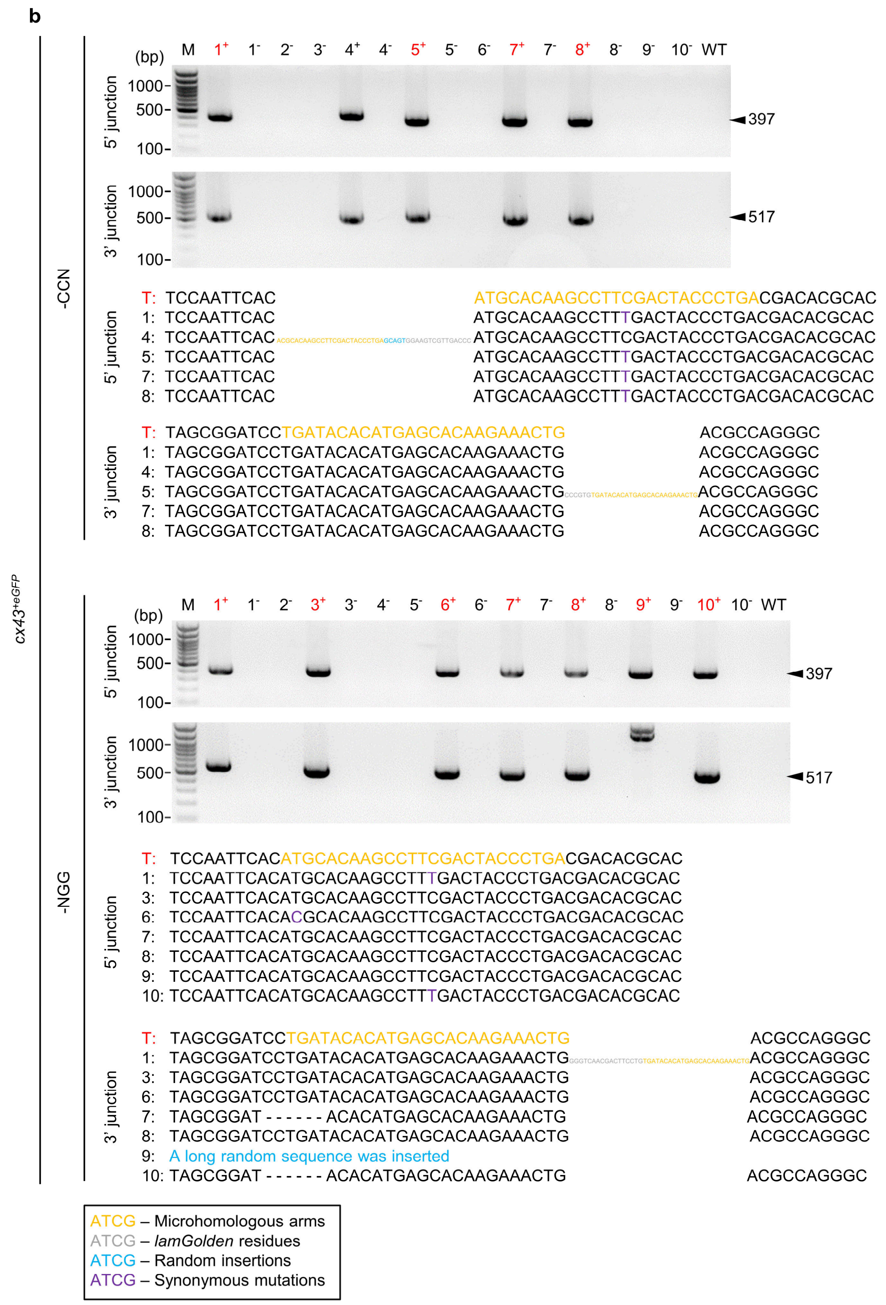

Supplementary Fig. 12. A comparison of the S-NGG-25 and S-CCN-25 strategies in generating desired F_0.

Junction PCR analysis of *cx43.4*$^{+/+eGFP}$ (a) and *cx43*$^{+/+eGFP}$ (b) F_1 generated by the S-CCN-25 or S-NGG-25 strategy. Ten randomly selected GFP-positive F_0 were screened for germline transmission. F_1 derived from outbreeding each F_0 with WT were examined for GFP expression patterns and genotypes. Numbers on the top of the gels represent F_1 generated by different F_0 zebrafish. “+” at

the upper right of each number means “GFP-positive” and “-” means “GFP-negative”. Desired F_1 were indicated in red. T, the desired sequence used as template for the alignment.

Supplementary Figure 13

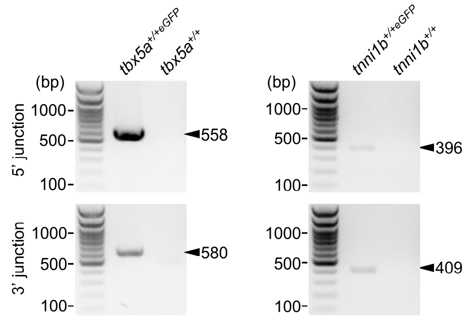

Supplementary Fig. 13. Junction PCR analysis of *tbx5a*$^{+/+eGFP}$ and *tnni1b*$^{+/+eGFP}$ F_1 zebrafish.

F_1 embryos carrying GFP-labeled *tbx5a* or *tnni1b* alleles were identified by 5’- and 3’-junction PCR analysis. Data are representatives of at least 10 F_1.

Supplementary Figure 14

F$_1$ progeny carrying fluorescence-labeled *connexin* allele					
cx23$^{+/+eGFP}$	√	*cx34.1*$^{+/+eGFP}$		*cx43.4*$^{+/+eGFP}$ (*cx43.4*$^{+/+mCherry}$)	√
cx27.5$^{+/+eGFP}$		*cx34.4*$^{+/+eGFP}$		*cx44.1*$^{+/+mCherry}$	√
cx28.1$^{+/+eGFP}$		*cx34.5*$^{+/+eGFP}$		*cx44.2*$^{+/+eGFP}$	
cx28.6$^{+/+eGFP}$		*cx35*$^{+/+eGFP}$		*cx45.6*$^{+/+eGFP}$	
cx28.8$^{+/+eGFP}$		*cx35.4*$^{+/+eGFP}$		*cx47.1*$^{+/+eGFP}$	
cx28.9$^{+/+eGFP}$		*cx36.7*$^{+/+eGFP}$		*cx48.5*$^{+/+mCherry}$	√
cx30.3$^{+/+eGFP}$	√	*cx39.4*$^{+/+eGFP}$		*cx52.6*$^{+/+eGFP}$	
cx30.9$^{+/+eGFP}$		*cx39.9*$^{+/+eGFP}$	√	*cx52.7*$^{+/+eGFP}$	
cx31.7$^{+/+eGFP}$		*cx40.8*$^{+/+eGFP}$		*cx52.9*$^{+/+eGFP}$	
cx32.2$^{+/+eGFP}$		*cx41.8*$^{+/+mCherry}$		*cx55.5*$^{+/+mCherry}$	
cx32.3$^{+/+eGFP}$		*cx43*$^{+/+eGFP}$	√	*cx79.8*$^{+/+eGFP}$	

Supplementary Fig. 14. A summary of successfully tagged *connexins* and whether or not fluorescence was observed in the KI F$_1$.

"✓" means that fluorescence-positive F$_1$ were obtained.

Supplementary Figure 15

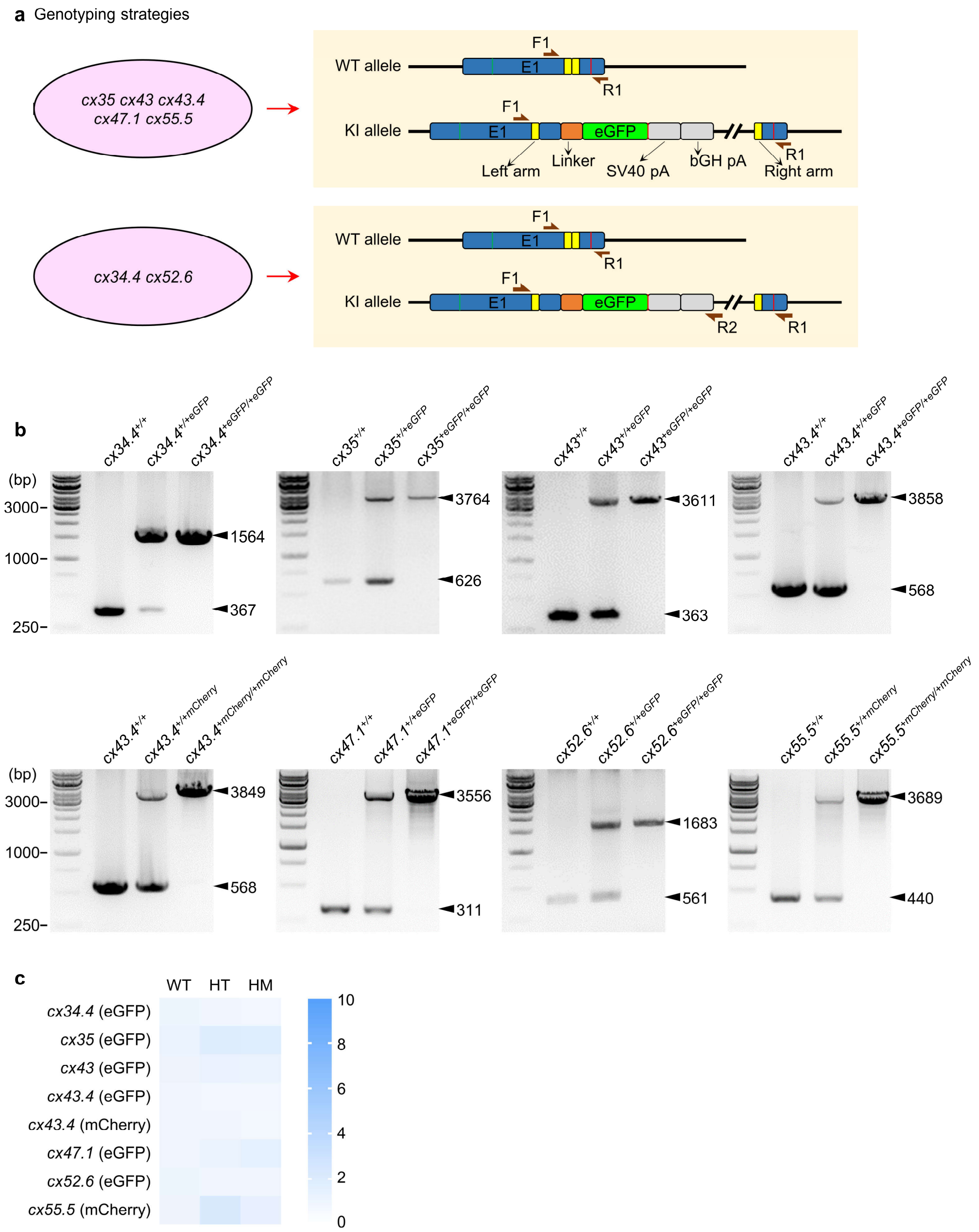

Supplementary Fig. 15. A strategy for genotyping *connexin* KI zebrafish.

(a) A schematic diagram of a genotyping method to identify WT, heterozygous (HT), or homozygous (HM) KI zebrafish. For *cx35*, *cx43*, *cx43.4*, *cx47.1*, and *cx55.5*, primers (F1 and R1) were designed to flank the homologous arms. For *cx34.4* and *cx52.6*, the reverse primer R2 targets the bGH polyA sequence and genotyping was done using primers F1, R1, and R2. **(b)** Genotyping results for *cx34.4, cx35*, *cx43*, *cx43.4*, *cx47.1*, *cx52.6,* and *cx55.5* KI. Data are representatives of at least 10 F_2. **(c)** Expression levels of *connexins* in

fluorescence-labeled HT or HM F_3 relative to WT after tagging. The total mRNA of genotype-confirmed WT, HT, or HM F_3 zebrafish for each *connexin* was extracted and used for RT-qPCR analysis. Expression of the tagged *connexin* in each of its F_3 progeny was normalized to that of *β-actin*, and then the threshold cycle ($2^{-\Delta\Delta Ct}$) method was used and the fold change of the *connexin* in each sample relative to the WT sample was plotted. At least 30 F_3 embryos per genotype were analyzed and 3 repeats were included for each sample in an RT-qPCR.

Supplementary Figure 16

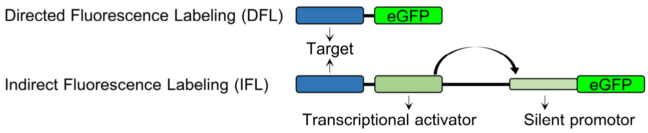

Supplementary Fig. 16. A schematic diagram of how the indirect fluorescence labeling (IFL) works.

Direct fluorescence labeling (DFL) generates fluorescent proteins (e.g. GFP) directly fused with the target protein by a linker. Indirect fluorescence labeling (IFL) indirectly produces GFP mediated by a transcriptional activator which is fused and co-expressed with the target protein, initiating GFP expression by binding a specific promoter ahead of the GFP CDS.

Supplementary Figure 17

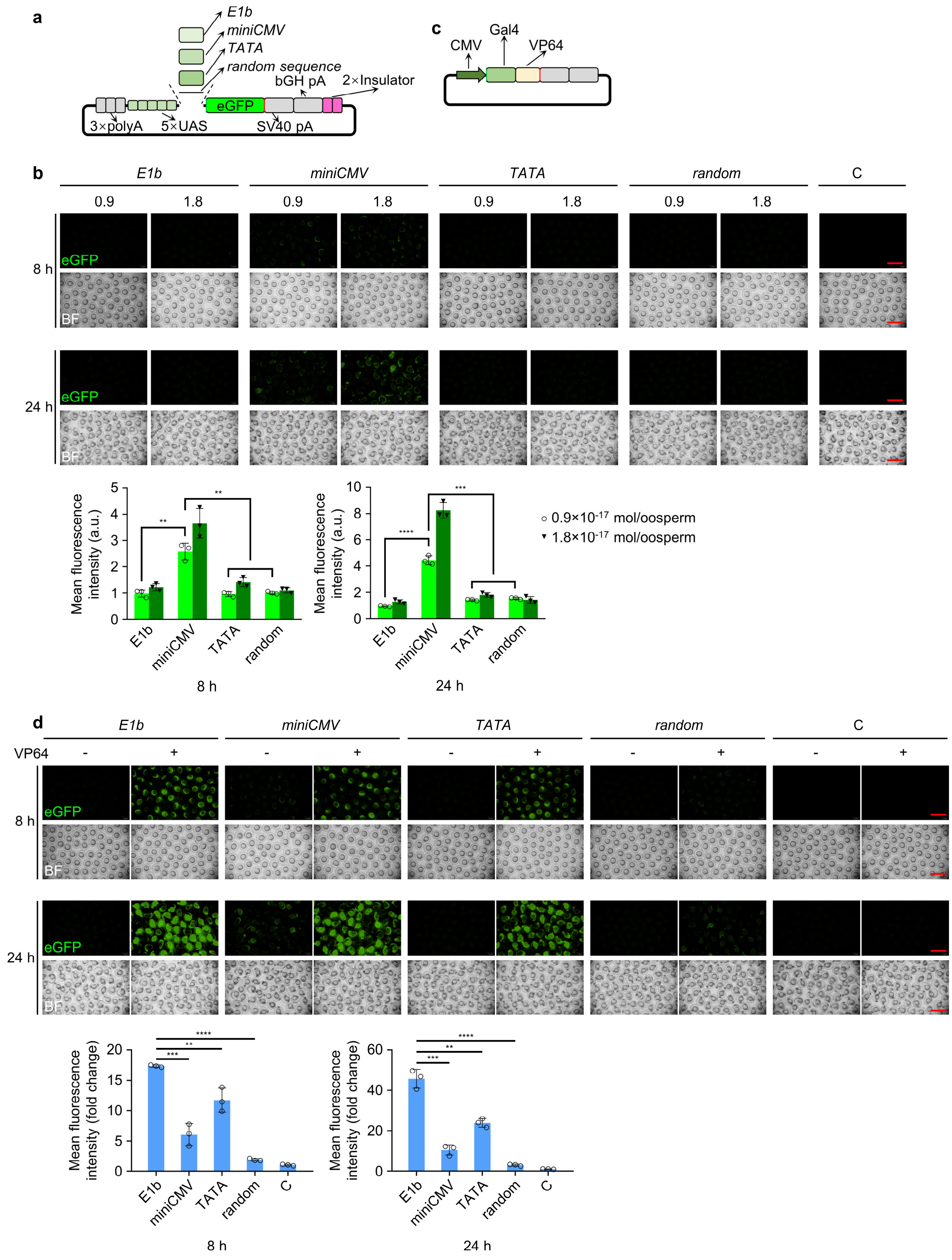

Supplementary Fig. 17. Promotor optimization for the fluorescence amplification system.

(a) A schematic diagram of the fluorescence reporter plasmid. Different promotors, including *E1b*, *miniCMV*, *TATA* (random sequence containing *TATA*

box), and random sequence (without *TATA* box), are located downstream of 5×nrUAS and upstream of eGFP CDS. **(b)** Comparison of leaky expression driven by different promotors. Different plasmids were microinjected into WT zebrafish embryos at the one-cell stage respectively. Images of embryos were taken at 8 hpf and 24 hpf to calculate MFI. C, vector control without promoters. Data are presented as mean ± SD of 3 independent experiments. *P* values were calculated using an unpaired Student *t* test. **, $P < 0.01$; ***, $P < 0.001$; ****, $P < 0.0001$. Scale bars, 2 mm. **(c)** A schematic diagram of the Gal4-VP64 transcriptional activator plasmid. Gal4-VP64 is driven by a CMV promotor. **(d)** Comparison of transcriptional activation of different promotors by Gal4-VP64. Gal4-VP64 and fluorescence reporter plasmids (0.9×10^{-17} mol per plasmid per embryo) were co-injected into 1-cell-stage WT zebrafish embryos. Images of embryos were taken at 8 hpf and 24 hpf to calculate MFI. C, vector control without promoters. Data are presented as mean ± SD of 3 independent experiments. *P* values were calculated using an unpaired Student *t* test. **, $P < 0.01$; ***, $P < 0.001$; ****, $P < 0.0001$. Scale bars, 2 mm.

Supplementary Figure 18

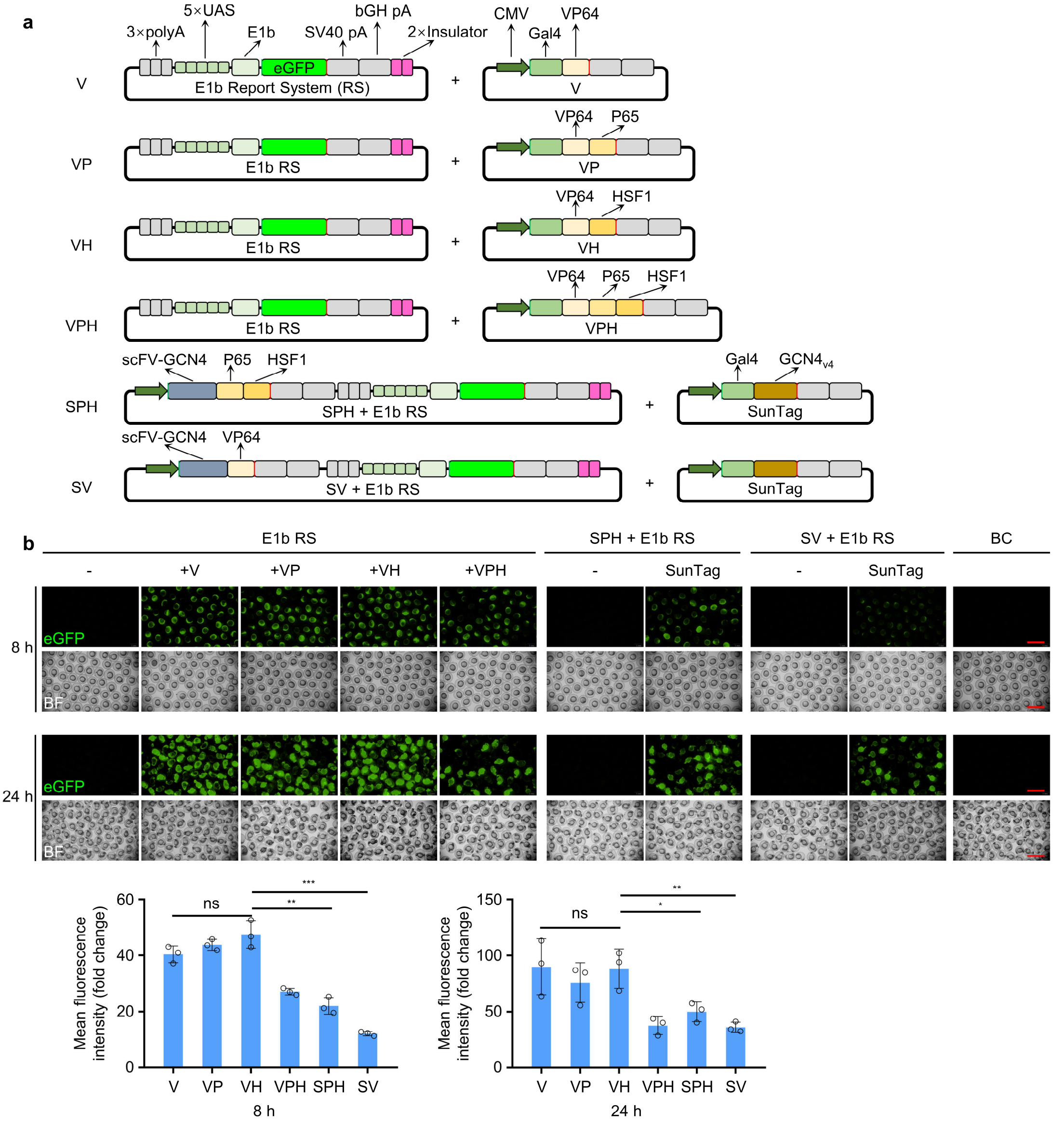

Supplementary Fig. 18. Optimization of the transcriptional activator for the fluorescence amplification system.

(a) Schematic diagrams of different transcriptional activation systems. Gal4-VP64 (V), Gal4-VP64-P65 (VP), Gal4-VP64-HSF1 (VH), or Gal4-VP64-P65-HSF1 (VPH) was co-injected with the *5×nrUAS-E1b-eGFP* plasmid into 1-cell-stage WT embryos (0.9×10^{-17} mol per plasmid per embryo). SunTag-P65-HSF1 (SPH) or SunTag-VP64 (SV) was cloned into the *5×nrUAS-E1b-eGFP* plasmid and was co-injected with the Gal4-GCN_4 plasmid into WT embryos (0.9×10^{-17}

mol per plasmid per embryo). **(b)** Comparison of fluorescence generated by different transcriptional activation systems. Images of embryos were taken at 8 hpf and 24 hpf to calculate MFI. BC, blank control without plasmids. Data are presented as mean ± SD of 3 independent experiments. P values were calculated using an unpaired Student t test. ns, not significant; *, $P < 0.05$; **, $P < 0.01$; ***, $P < 0.001$. Scale bars, 2 mm.

Supplementary Figure 19

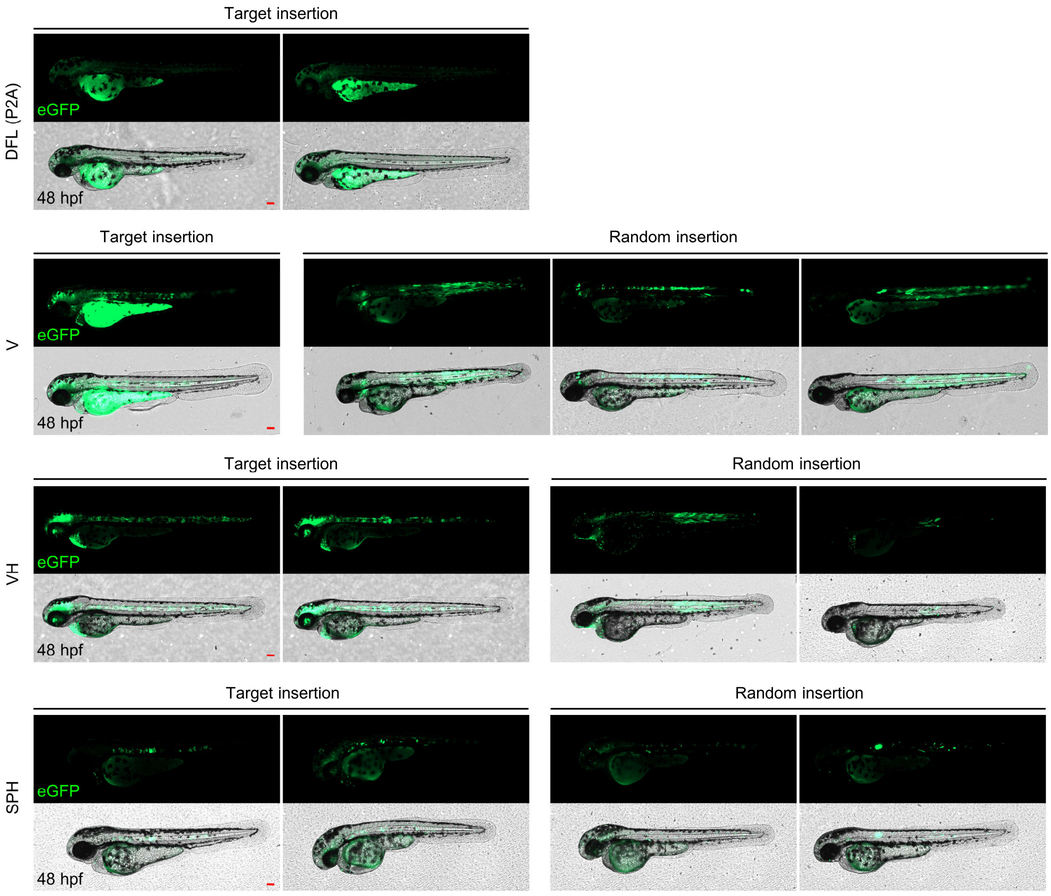

Supplementary Fig. 19. Images of *cx43.4* KI embryos using DFL, V, VH, or SPH strategy.

Different KI strategies were performed to generate *cx43.4* KI. After co-injection of the Cas9/sgRNA system and the IFL or DFL donor into 1-cell-stage WT embryos, images of microinjected GFP-positive embryos were collected at 48 hpf. IFL-labeled GFP-positive F_0 embryos were classified into target-insertion and random-insertion groups based on the expression patterns of *cx43.4* in the DFL group (see also Fig. 1d). F_0 embryos of the target-insertion groups were raised to adulthood for germline transmission screen. Scale bars, 100 μm. Images are representatives of at least 30 F_0 for each strategy.

Supplementary Figure 20

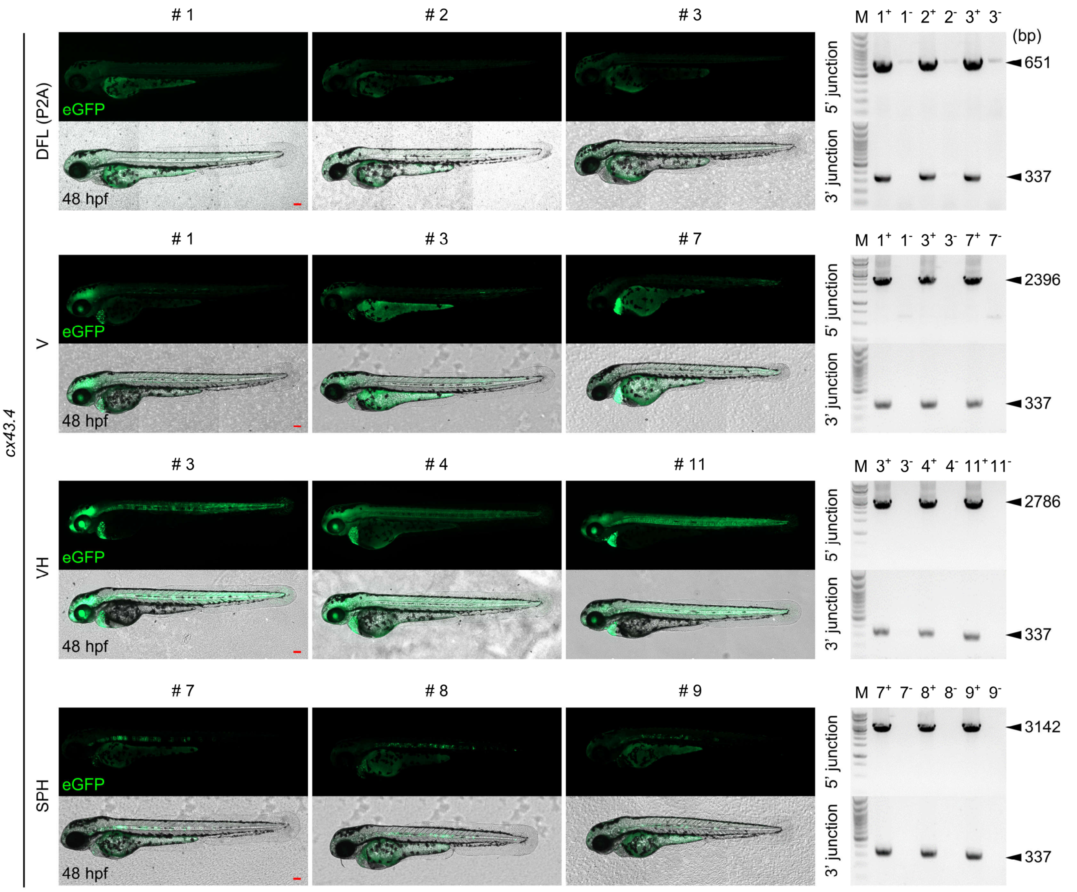

Supplementary Fig. 20. Images and junction PCR analysis of F_1 *cx43.4*-KI zebrafish generated by DFL, V, VH, or SPH strategy.

Different KI strategies were performed to fluorescence-label *cx43.4*. (Left panel) Images of GFP-expressing desired *cx43.4*-KI F_1 embryos derived from three different F_0 adults generated by using DFL, V, VH, or SPH strategy. Images were taken at 48 hpf. The number of the corresponding F_0 zebrafish was shown. Scale bars, 100 μm. (Right panel) Junction PCR tests were performed to confirm genotypes of the desired F_1. GFP-negative littermates were used as negative controls. Numbers on the top of the gels represent F_1 zebrafish generated by F_0 with different numbers shown in (Left panel). "+" at the upper right of each number means GFP-positive F_1 embryos, and "-" means GFP-negative F_1 embryos.

Supplementary Figure 21

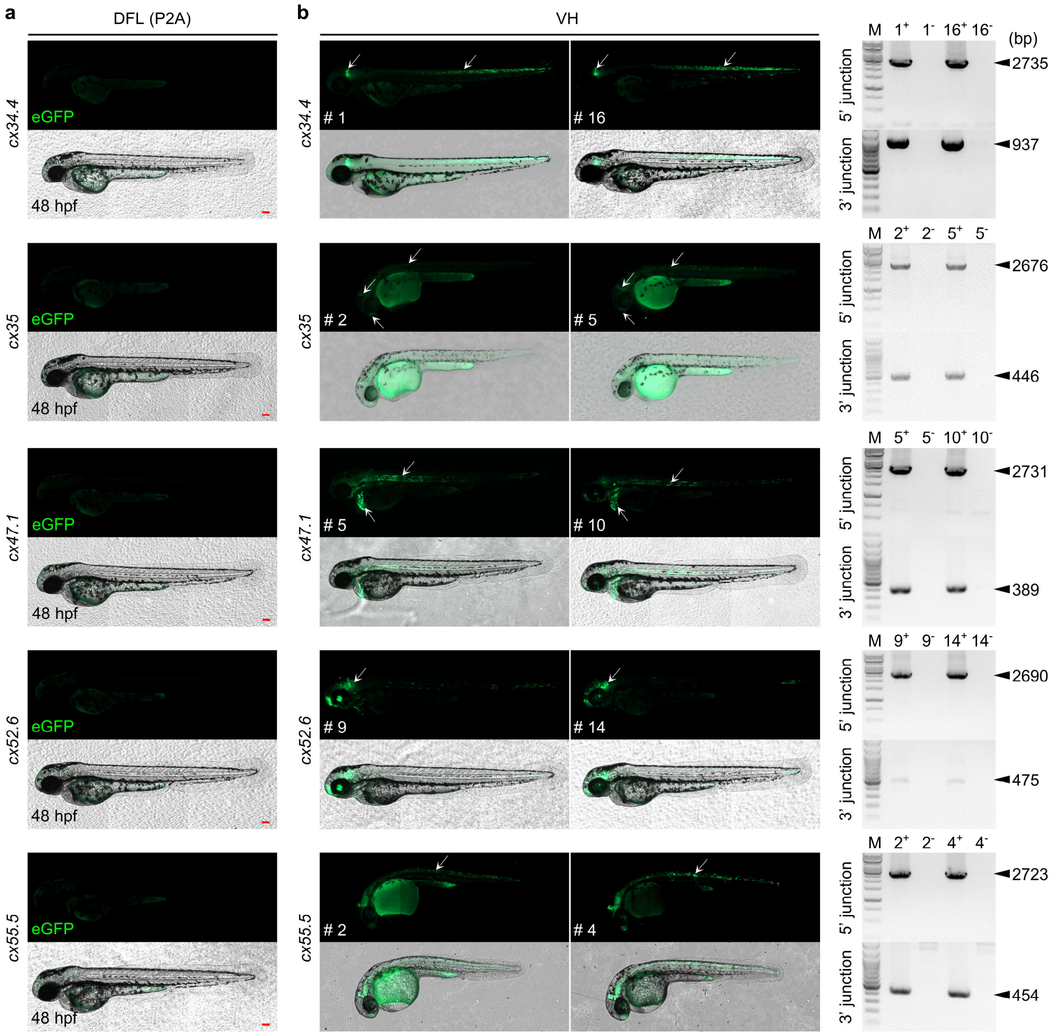

Supplementary Fig. 21. Images of F_1 embryos generated by the DFL or VH strategy for *cx34.4*, *cx35*, *cx47.1*, *cx52.6*, or *cx55.5* KI.

(a) F_1 embryos generated by the 2A-linker-mediated DFL strategy were included as controls and no fluorescence was detected in these F_1. Images are representatives of at least 10 F_1 at 48 hpf. **(b)** Left panel, for each VH-engineered *connexin*, F_1 carriers were derived from outbreeding at least two F_0 founders with WT. Images are representatives of at least 10 at 48 hpf. The number of the corresponding F_0 was labeled at the bottom left. Scale bars, 100 μm. Right panel, junction PCR was performed to confirm genotypes of the VH strategy-generated F_1. GFP-negative littermates were used as negative controls. The numbers on the top of the gels are the numbers of the

corresponding F_0 zebrafish, the same as those shown in the left panel. “+” means GFP-positive F_1, and “-“ means GFP-negative F_1.

Supplementary Figure 22

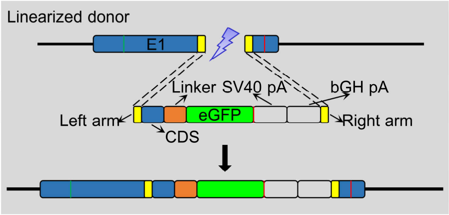

Supplementary Fig. 22. A schematic diagram of the linearized dsDNA-mediated KI strategy.

Linearized dsDNA containing functional cassettes was amplified by PCR and was used as the donor. When MMEJ-repair happens, the linearized donor would be integrated into the target gene without a redundant sequence.

Supplementary Figure 23

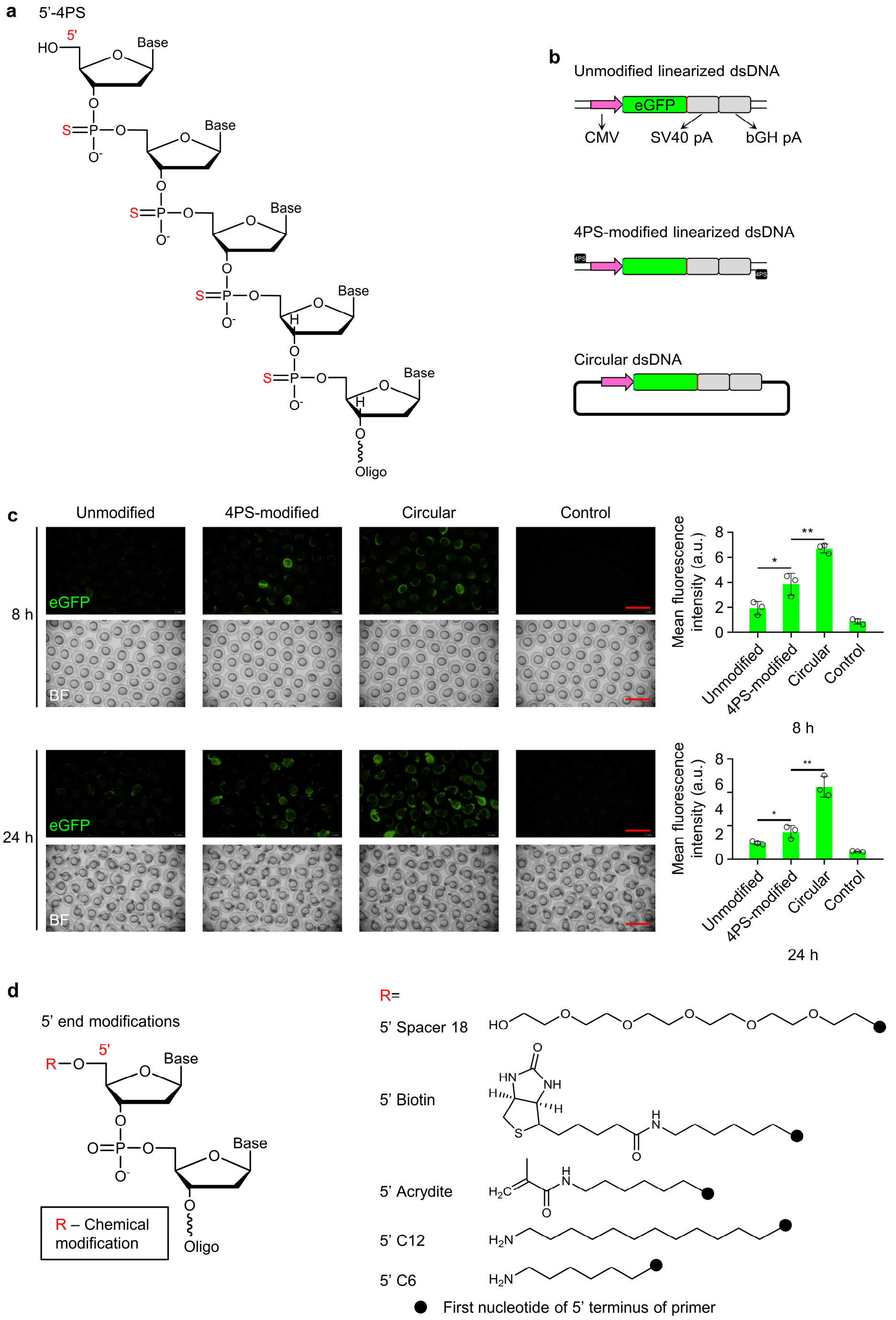

Supplementary Fig. 23. 5'-end modifications improved the stability of dsDNAs.

(a) Structure of the 5'-4PS modification. Replacing 1 P=O in 1 phosphate group between 2 deoxyribonucleotides generates 1 P=S and replacing 4 P=O in 4 phosphate groups between 5 deoxyribonucleotides generates 4 P=S, and the like. **(b)** A schematic diagram of the unmodified linearized dsDNA, 4PS-

modified linearized dsDNA, and circular dsDNA. All of the dsDNAs have the same GFP expression cassette, including a *CMV* promotor, a GFP coding sequence, and polyA signals. 5'-4PS modifications were added to the 5' terminus of the linearized dsDNAs by using modified PCR primers. **(c)** dsDNAs in (b) were microinjected into one-cell-stage WT zebrafish embryos at 0.9×10^{-17} mol/embryo. Images of embryos were taken at 8 hpf and 24 hpf to calculate MFI. Control, the vector plasmid control without functional cassettes. Data are presented as mean ± SD of 3 independent experiments. *P* values were calculated using an unpaired Student *t* test. *, $P < 0.05$; **, $P < 0.01$. Scale bars, 2 mm. **(d)** Structures of other 5'-chemical modifications (R), including spacer18, biotin, acrydite, C12, and C6. These 5'-chemical modifications were added to the first deoxyribonucleotide of the 5' terminus of each primer.

Supplementary Figure 24

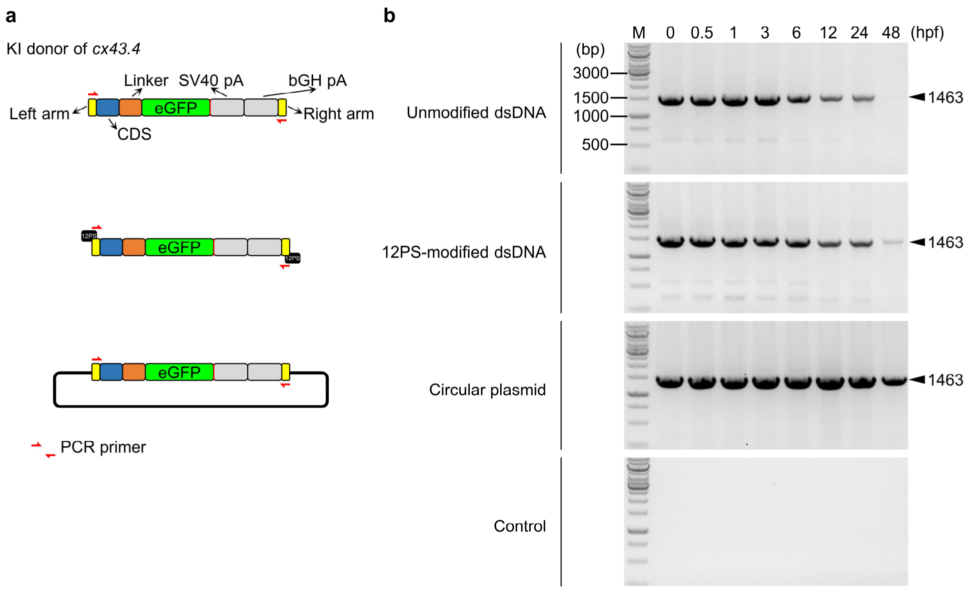

Supplementary Fig. 24. Comparison of the degradation of different forms of dsDNAs in embryos.

(a) A schematic diagram of three types of KI donors for *cx43.4*, the unmodified linearized dsDNA, the 12PS-modified linearized dsDNA, and the circular plasmid. PCR primers were designed in the left and right arms. **(b)** Time-course analysis of the degradation of different forms of dsDNA donors shown in (A). Equal amount (0.9×10^{-17} mol/embryo) of each donor was injected into one-cell-stage WT embryos. Five embryos per donor type were randomly selected and mixed to extract total DNA at different time points. DNA samples were then subjected to PCR analysis. Data are representatives of 3 independent experiments.

Supplementary Figure 25

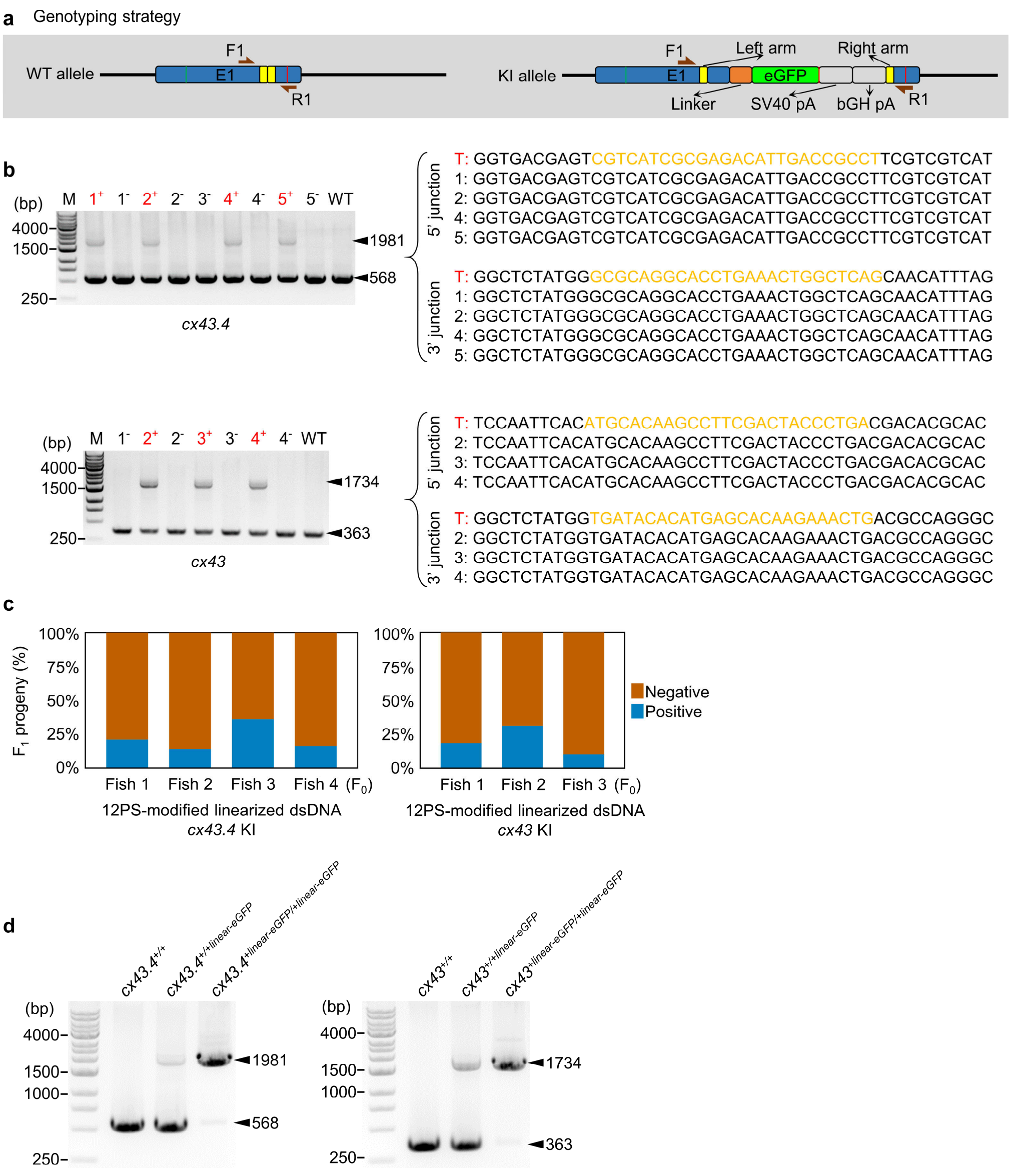

Supplementary Fig. 25. GFP-labeled *cx43* or *cx43.4* alleles were successfully generated by the 12PS-modified linearized dsDNA mediated KI.

(a) A schematic diagram of a genotyping method for the linearized dsDNA mediated KI. Primers were designed to flank the homologous arms. **(b)** Genotyping and sequencing analysis of GFP-positive *cx43*- or *cx43.4*-KI F_1 progeny from outbreeding F_0 adults generated by using the 5'-12PS donors. Numbers on the top of the gels represent F_1 zebrafish generated by F_0 labeled with different numbers. "+" at the upper right of each number means "GFP-

positive” and “-” means “GFP-negative”. Desired F_1 were indicated in red. T, the desired sequence used as the template for the alignment. **(c)** Mosaicism of the germline of F_0 founders for *cx43.4* and *cx43* KI generated by using the 12-PS modified linearized dsDNA was determined by the percentage of F_1 carrying the KI cassette. GFP-positive *cx43.4*$^{+/+eGFP}$ or *cx43*$^{+/+eGFP}$ F_1 were shown in blue and GFP-negative *cx43.4*$^{+/+}$ or *cx43*$^{+/+}$ F_1 were in orange. At least 60 F_1 were examined for each PCR-positive F_0 founder. **(d)** Genotyping of F_2 progeny obtained from inbreeding GFP-positive *cx43*- or *cx43.4*-KI F_1 zebrafish. Data are representatives of at least 10 F_2.

Supplementary Figure 26

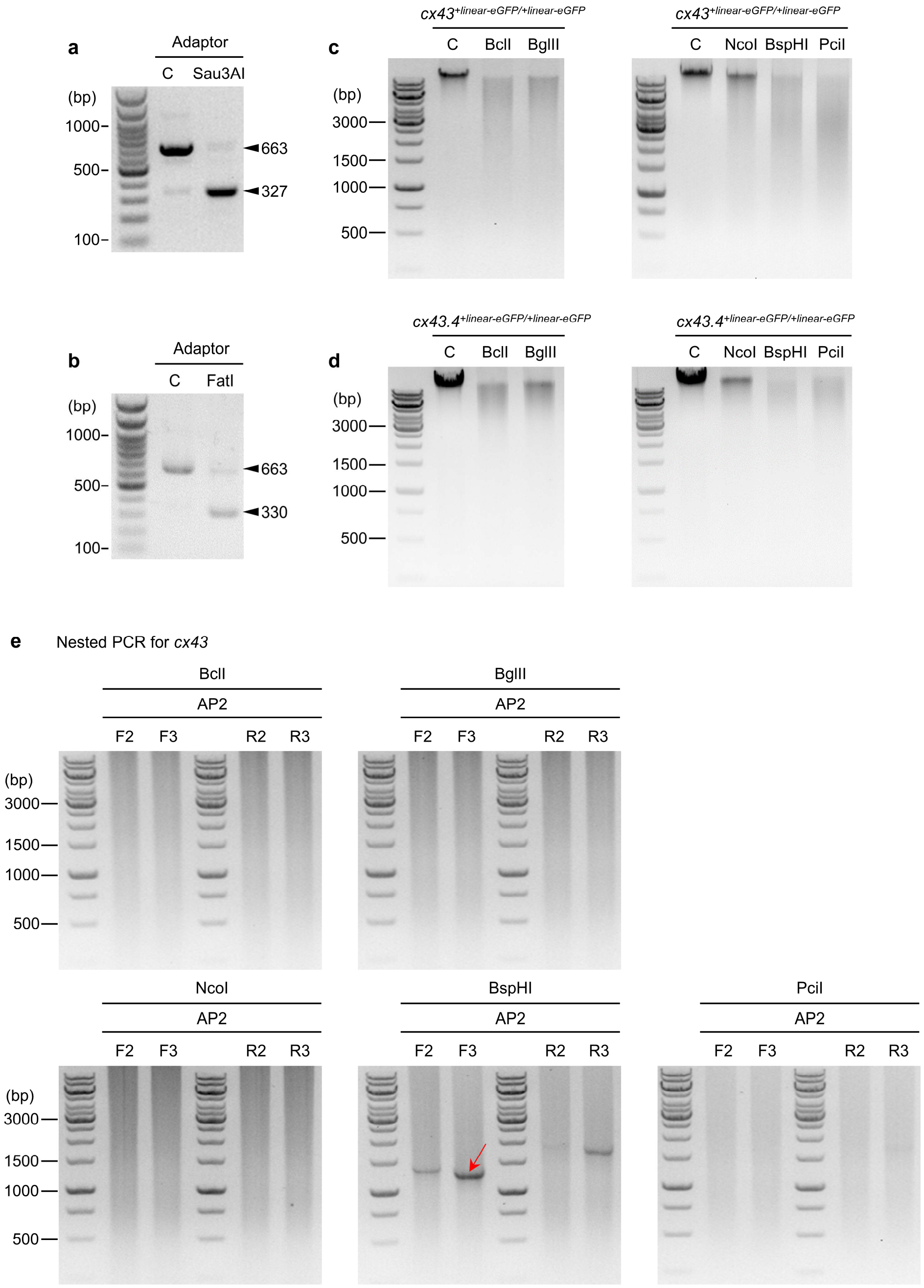

Sequence blast of BspHI-F3

Danio rerio connexin 43, mRNA (cDNA clone MGC:55225 IMAGE:5915503), complete cds

Sequence ID: BC049297.1 **Length:** 2821 **Number of Matches:** 1

Range 1: 1395 to 1675 GenBank Graphics ▼ Next Match ▲ Previous Match

Score	Expect	Identities	Gaps	Strand
520 bits(281)	1e-142	281/281(100%)	0/281(0%)	Plus/Plus

```
Query  1     GCAGAGCTAACATGCCCTAACTACCGAACAGTCAAGGGGGCTCGTCCGGGACGAACGGCA  60
             ||||||||||||||||||||||||||||||||||||||||||||||||||||||||||||
Sbjct  1395  GCAGAGCTAACATGCCCTAACTACCGAACAGTCAAGGGGGCTCGTCCGGGACGAACGGCA  1454

Query  61    CTGAACTTGCCACGTCTCTCAACTCAGCCCACCAGAACAGACCTTGGCGATGTGATTGAT  120
             ||||||||||||||||||||||||||||||||||||||||||||||||||||||||||||
Sbjct  1455  CTGAACTTGCCACGTCTCTCAACTCAGCCCACCAGAACAGACCTTGGCGATGTGATTGAT  1514

Query  121   TTTTGTTGTGCTTGTTGAAATGCCACACAATGATTCCACTTTAACACTTTGCACTCCTAC  180
             ||||||||||||||||||||||||||||||||||||||||||||||||||||||||||||
Sbjct  1515  TTTTGTTGTGCTTGTTGAAATGCCACACAATGATTCCACTTTAACACTTTGCACTCCTAC  1574

Query  181   AGTTTGTGTAGATTTGTGTTCTAAcccccccTACAGTCGATCCCGGTTATAACGTCAATC  240
             ||||||||||||||||||||||||||||||||||||||||||||||||||||||||||||
Sbjct  1575  AGTTTGTGTAGATTTGTGTTCTAACCCCCCCTACAGTCGATCCCGGTTATAACGTCAATC  1634

Query  241   GTTCGCATGATCTCAAATCCAACAGGGTGTTCACAGTCATG  281
             |||||||||||||||||||||||||||||||||||||||||
Sbjct  1635  GTTCGCATGATCTCAAATCCAACAGGGTGTTCACAGTCATG  1675
```

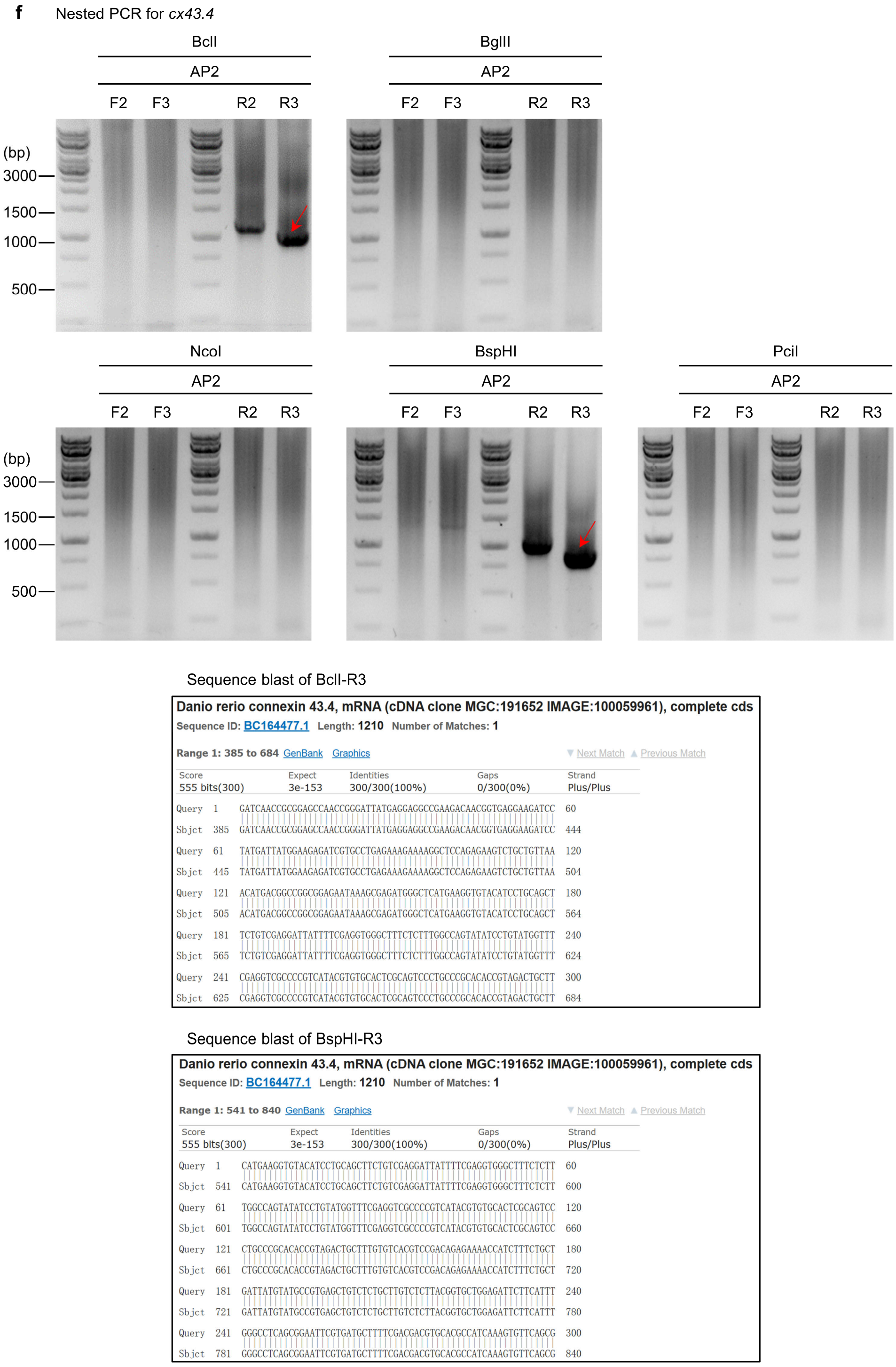

Supplementary Fig. 26. Examination of random integration by the Cyclic Digestion and Ligation-Mediated PCR (CDL-PCR) method.

(a and b) Preparation of the adaptors. Pre-adaptors were amplified by PCR from the pMD18T-APS plasmid. Purified pre-adaptors were enzymatically digested by Sau3AI and FatI, respectively. “C”, the undigested pre-adaptor

control. **(c and d)** Enzymatic digestion of the genomic DNA. Genomic DNA of *cx43.4*$^{+linear\text{-}eGFP/+linear\text{-}eGFP}$ or *cx43*$^{+linear\text{-}eGFP/+linear\text{-}eGFP}$ zebrafish was extracted from the caudal fins using the Genomic DNA Clean & Concentrator-10 Kit, and then was enzymatically digested by BclI, BglII, NcoI, BspHI, and PciI, respectively. “C”, the undigested genomic DNA control. **(e and f)** Integration was detected by nested PCR and confirmed by sequencing. BclI- or BglII-digested genomic DNA was ligated with the Sau3AI-digested adaptor by T4 DNA ligase. NcoI-, BspHI-, or PciI-digested genomic DNA was ligated with the FatI-digested adaptor by T4 DNA ligase. Nested PCR was performed to detect integration in the ligated genomic DNA. “AP2” was the primer of the adaptor, “F2”, “F3”, “R2”, and “R3” were primers of the genomic DNA. Amplified PCR products were then subjected to sequencing analysis.

Supplementary Figure 27

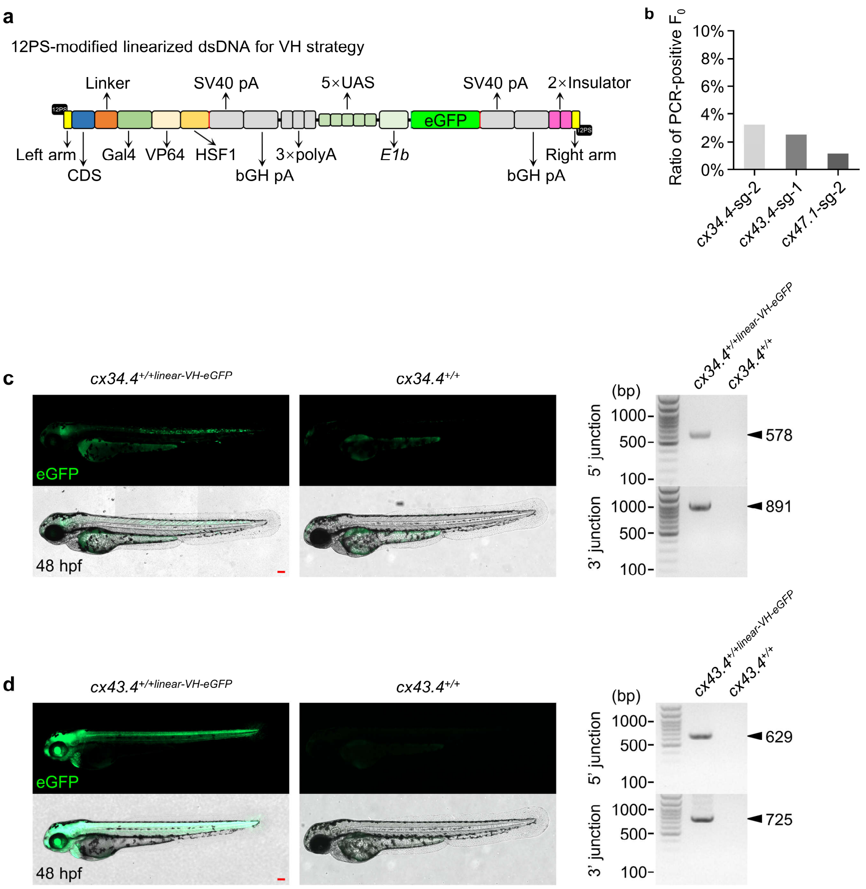

Supplementary Fig. 27. A combination of the 5'-modified linearized dsDNA with the VH-mediated KI strategy.

(a) A schematic diagram of a 12PS-modified linearized dsDNA for the VH strategy. The 12PS-modification was added to the 5'-terminus of the microhomologous arms. **(b)** Ratios of the PCR-positive F_0 after KI using the 12PS-modified linearized dsDNA-mediated VH strategy for some *connexins*. One high-efficiency sgRNA was used for each *connexin*. Genomic DNA was isolated from the caudal fins of one-month-old F_0 and used for 5'-junction PCR to identify PCR-positive F_0. At least 800 F_0 were tested for each gene. The ratio of PCR-positive F_0 in all tested F_0 zebrafish was then calculated. **(c and d)** Images of F_1 larvae for *cx34.4* or *cx43.4* KI generated by the 12PS-modified linearized dsDNA-mediated VH strategy. F_1 embryos were identified by 5'- and 3'-junction PCR analysis and were representatives of 10 PCR-positive F_1.

Images were taken at 48 hpf. Scale bars, 100 μm.

Supplementary Figure 28

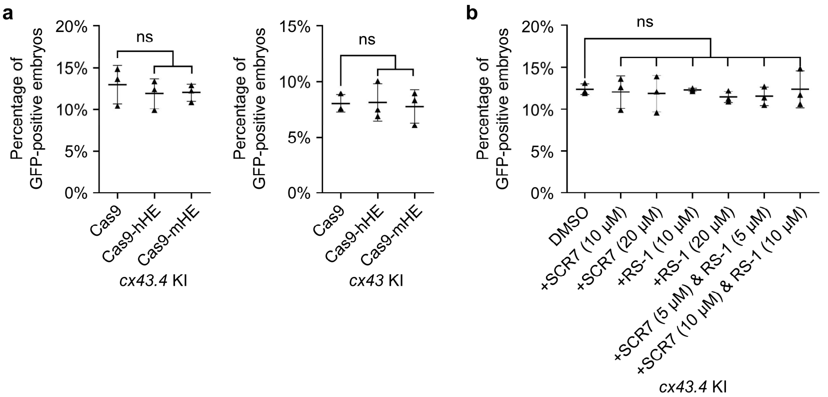

Supplementary Fig. 28. Effects of CtIP, SCR7, and RS-1 on KI efficiency.

(a) Percentages of GFP-positive embryos obtained by using Cas9, Cas9-hHE, or Cas9-mHE to perform KI for *cx43.4* or *cx43*. Cas9, Cas9-hHE, or Cas9-mHE mRNA was co-injected with the donor plasmid, sgRNA for *cx43* or *cx43.4*, and *lamGolden* sgRNA into at least 200 WT embryos at the one-cell stage. GFP-positive embryos were counted at 48 hpf. Data represent mean ± SD of 3 independent experiments. *P* values were calculated using an unpaired Student *t* test. ns, not significant. **(b)** Percentages of GFP-positive embryos obtained by treatment of SCR7 or RS-1 after microinjection of Cas9 mRNA, donor plasmid, sgRNA for *cx43.4*, and *lamGolden* sgRNA into at least 200 WT embryos at the one-cell stage. SCR7, RS-1, or SCR7 & RS-1 were used at different concentrations for 6 hours. DMSO was used as the vehicle control. GFP-positive embryos were counted at 48 hpf. Data represent mean ± SD of 3 independent experiments. *P* values were calculated using an unpaired Student *t* test. ns, not significant.

Supplementary Fig. 29. Uncropped agarose gels are shown below.

Including Supplementary Fig. 3b-c, 4, 6c, 7a, 7c, 9a-h, 10b, 11b, 12a-b, 13, 15b, 20, 21, 24, 25b, 25d, 26a-f, 27c.

Supplementary Fig. 3b

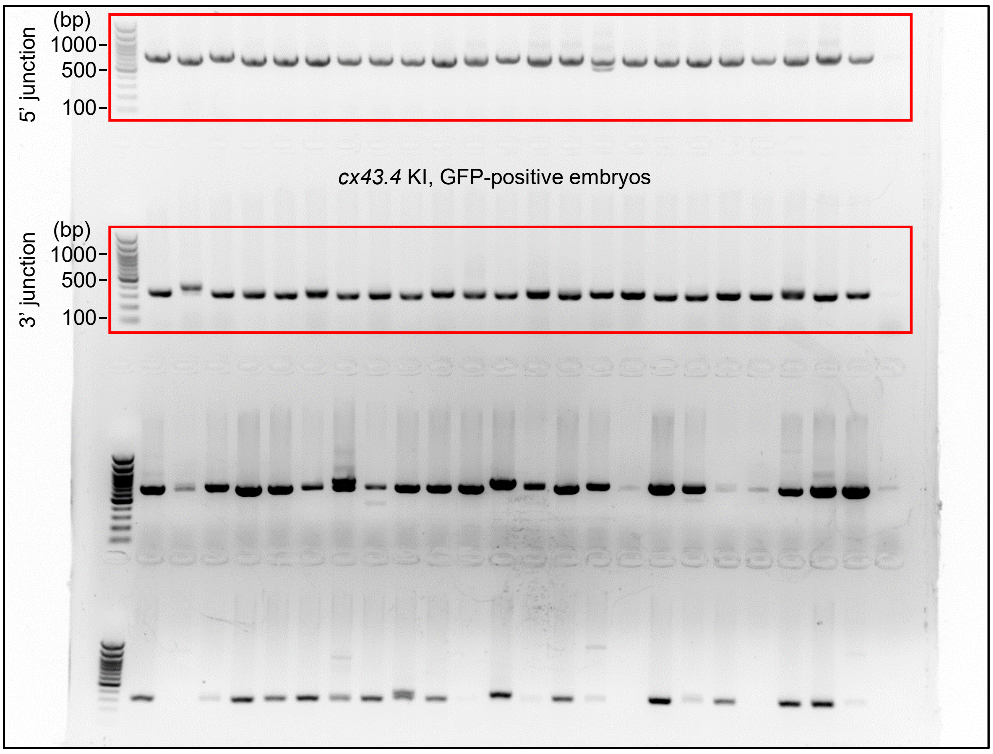

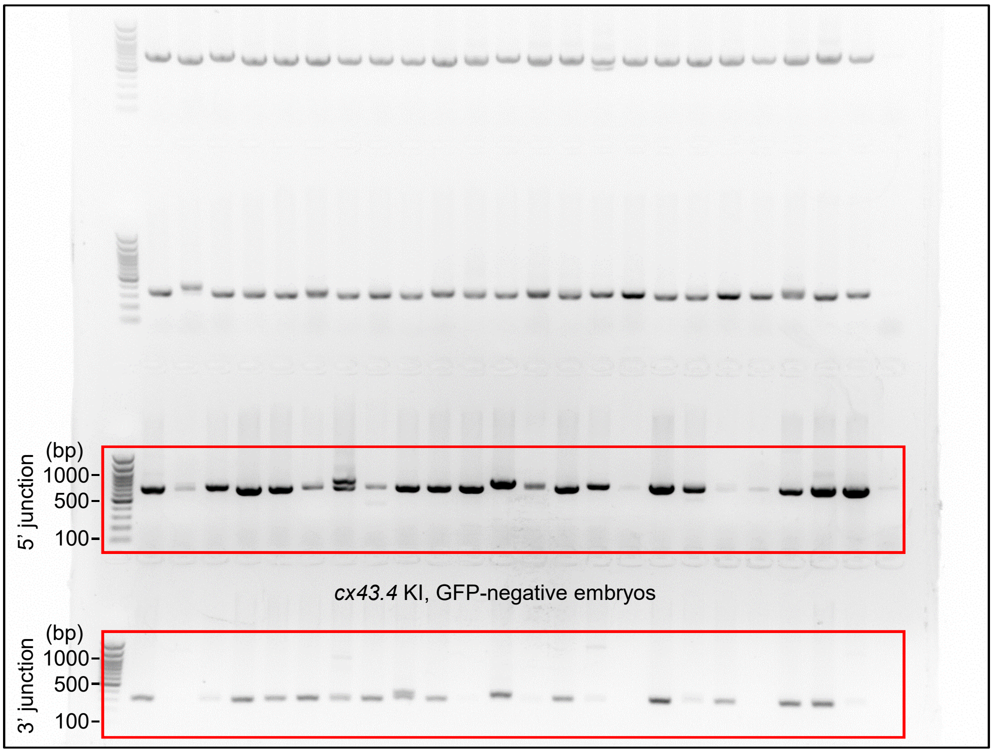

Supplementary Fig. 3c

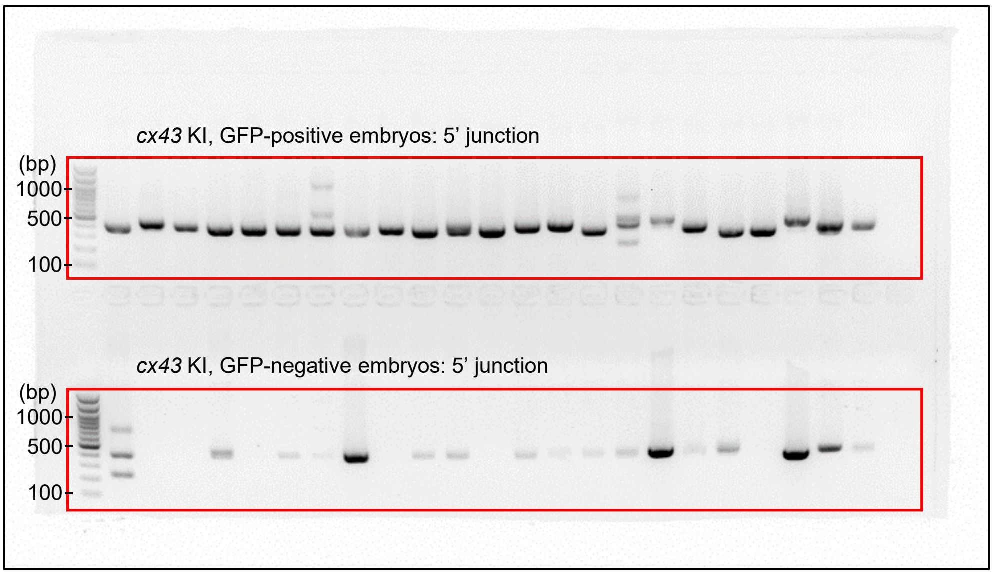

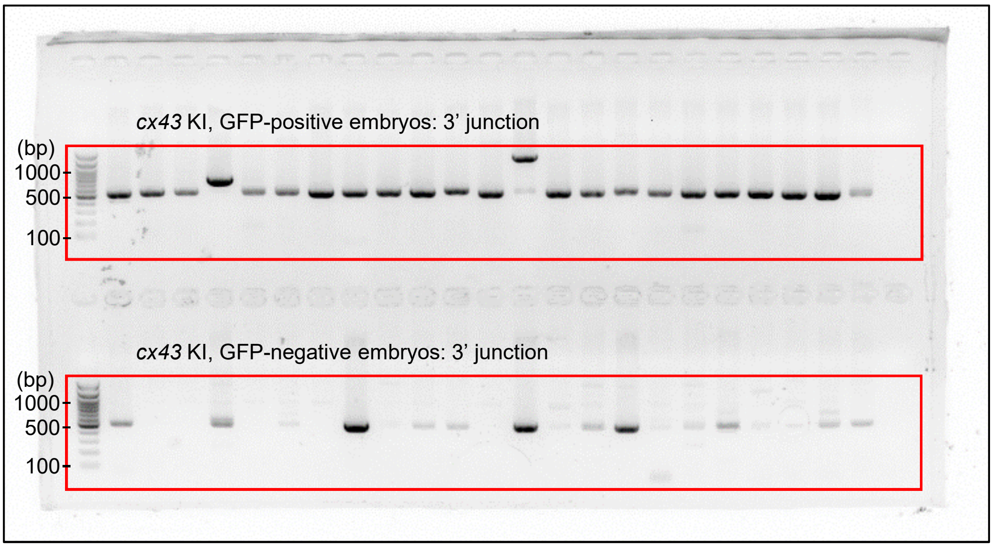

Supplementary Fig. 4

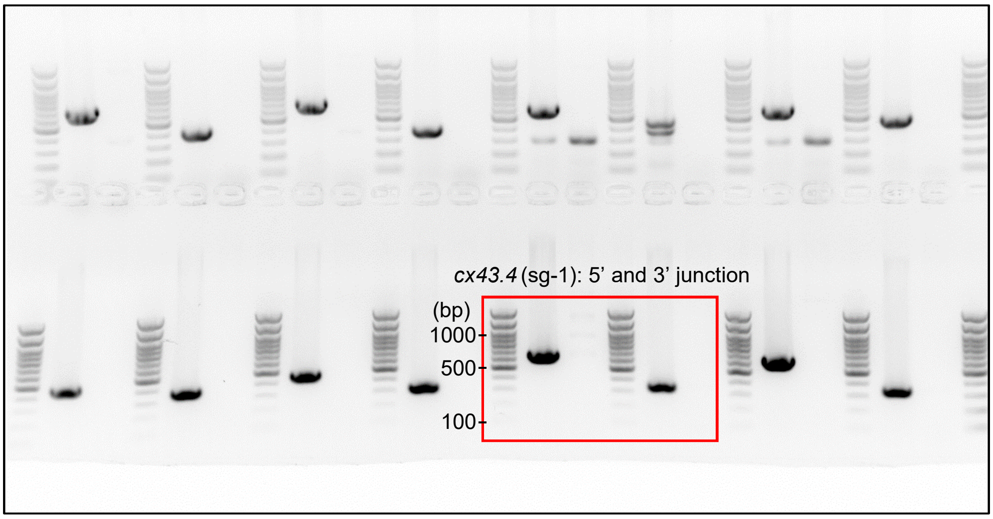

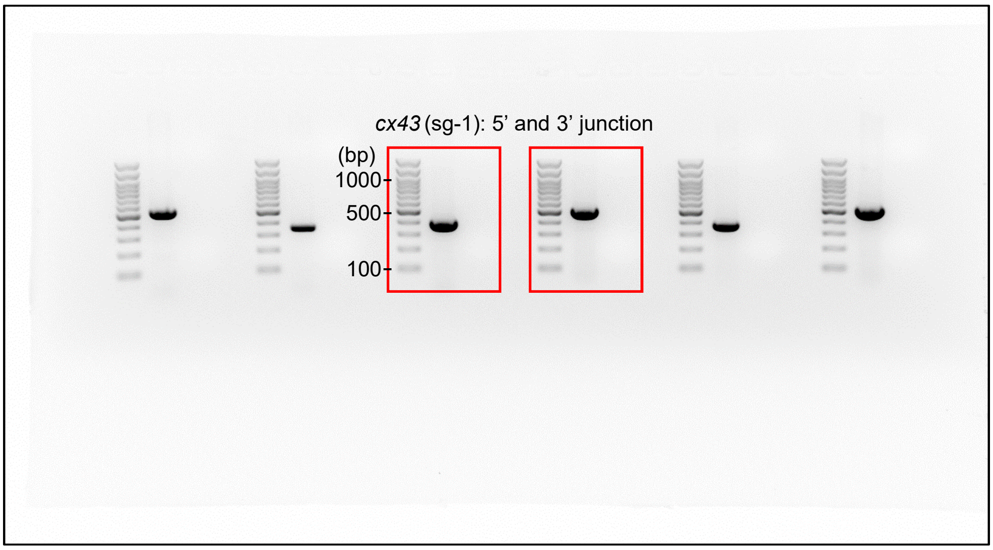

Supplementary Fig. 6c

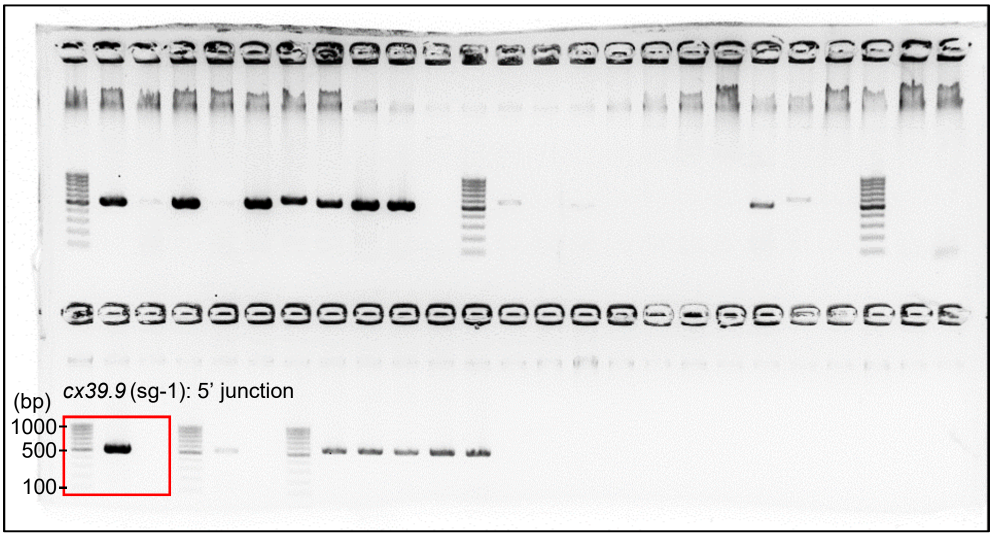

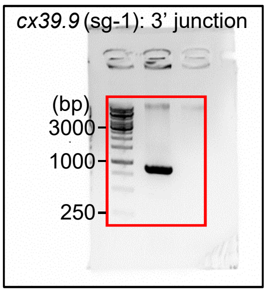

Supplementary Fig. 7a

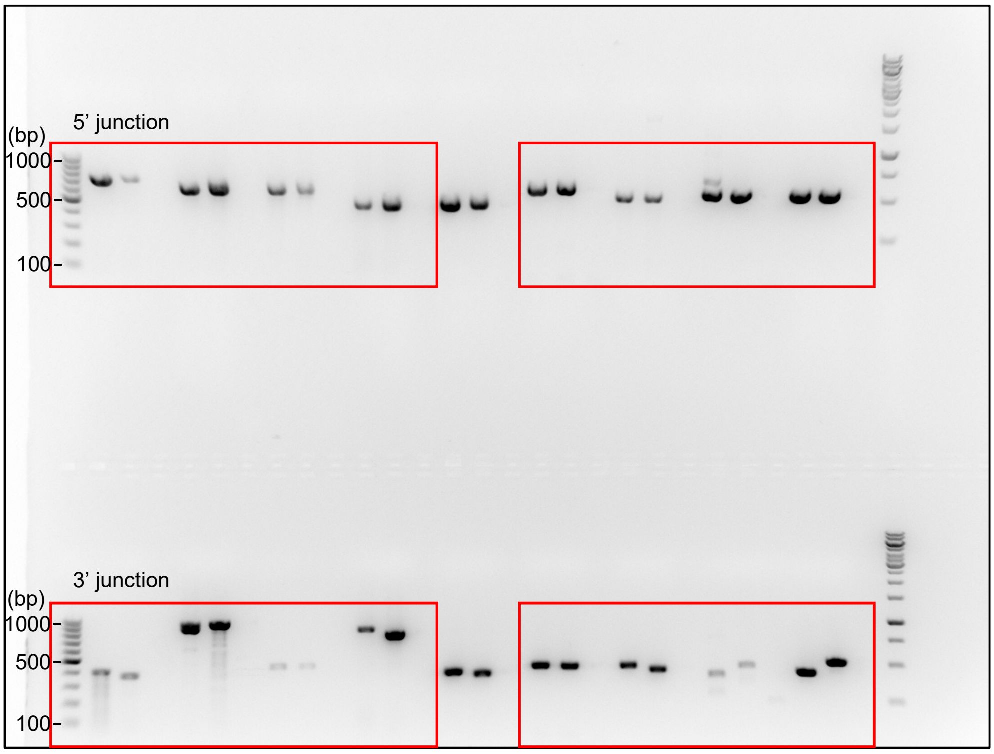

Supplementary Fig. 7c

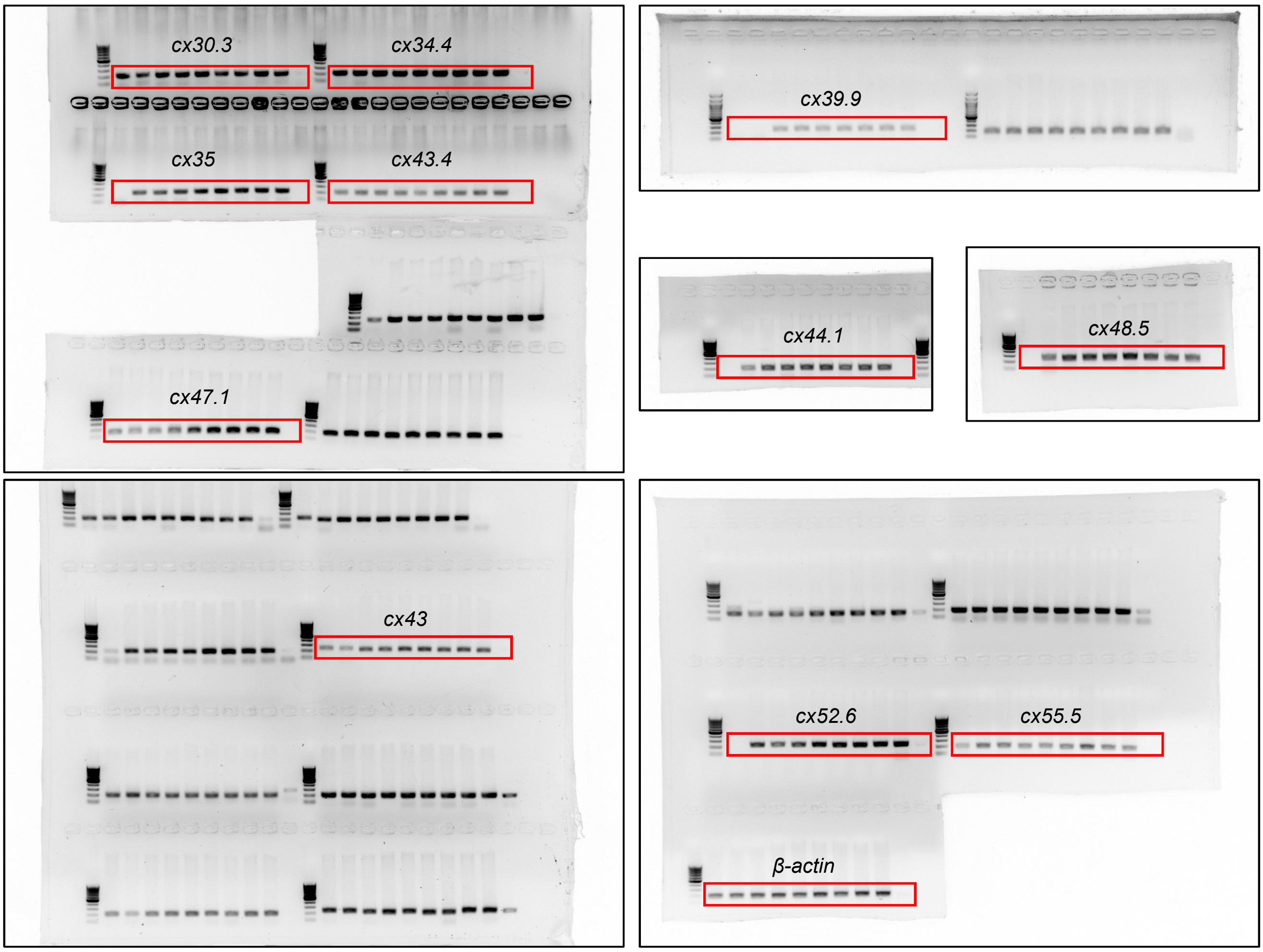

Supplementary Fig. 9a

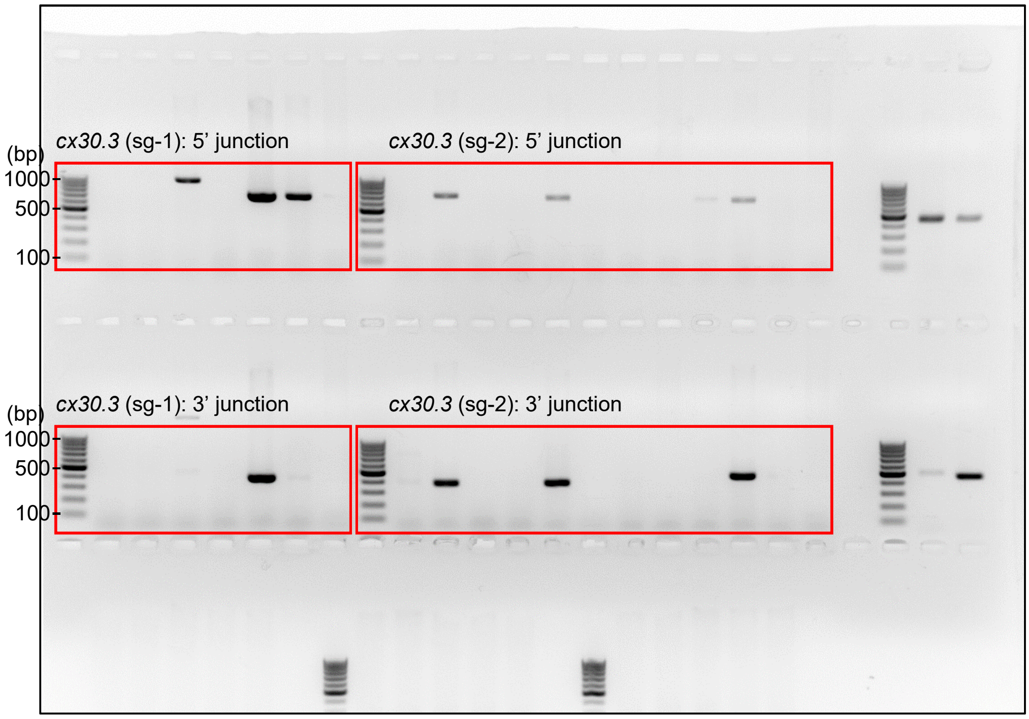

Supplementary Fig. 9b

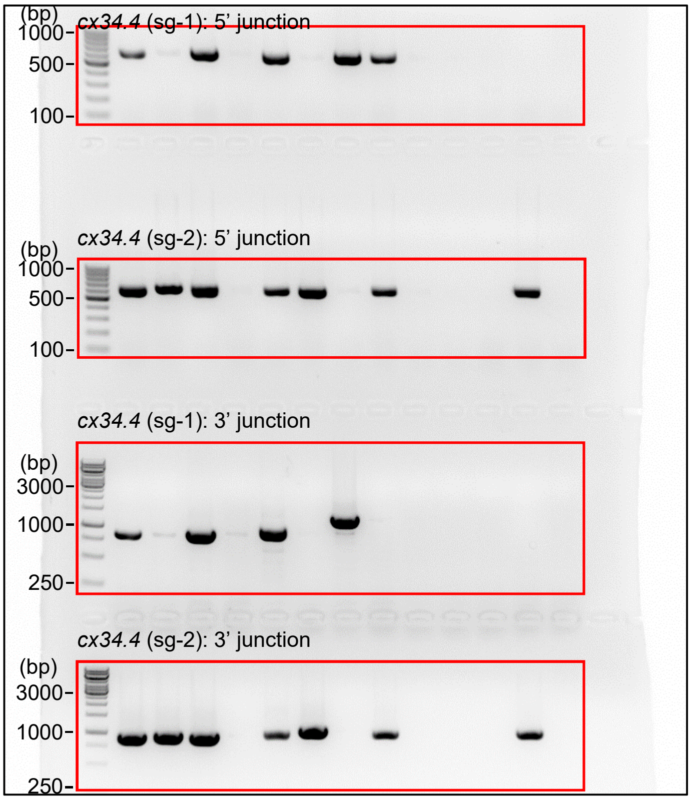

Supplementary Fig. 9c

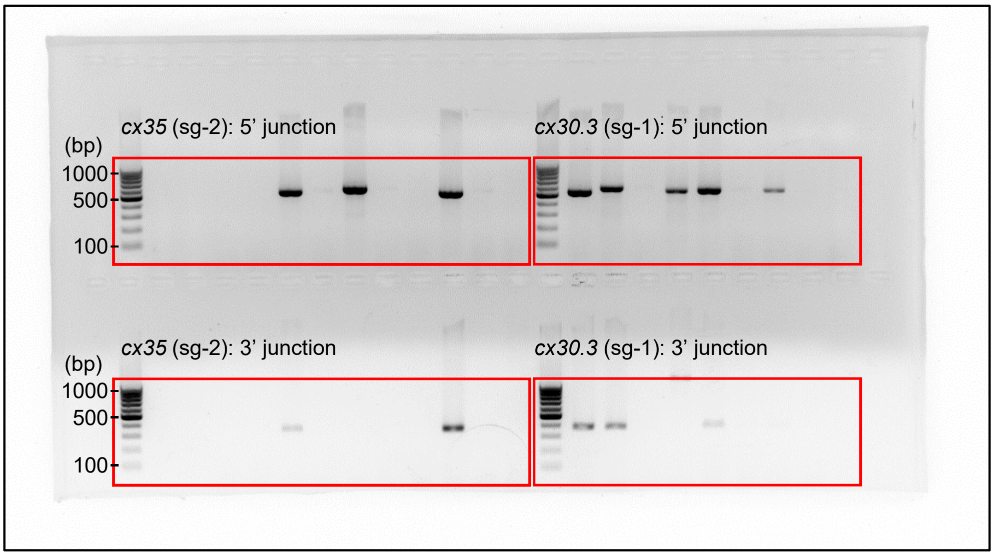

Supplementary Fig. 9d

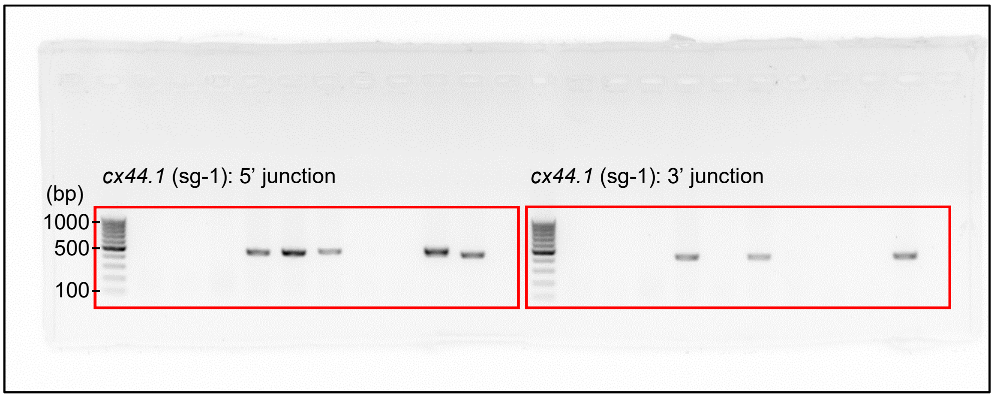

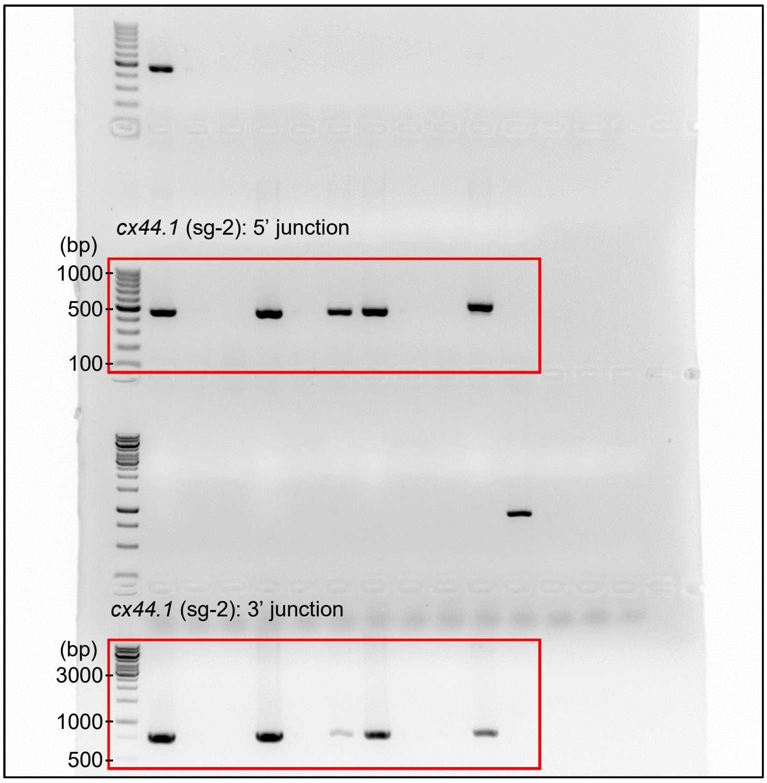

Supplementary Fig. 9e

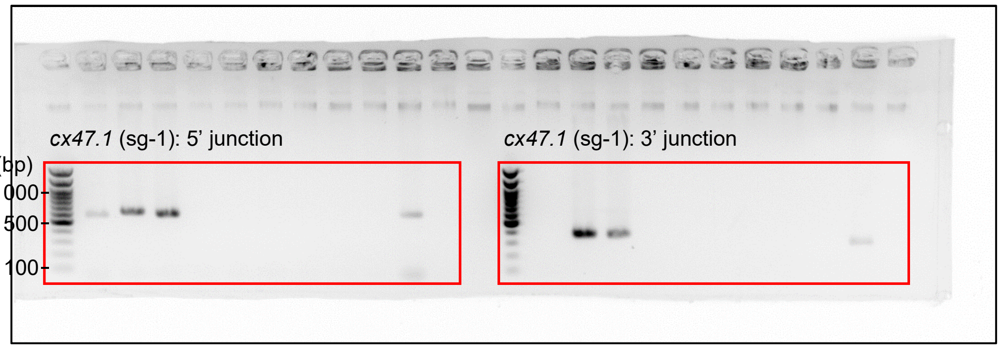

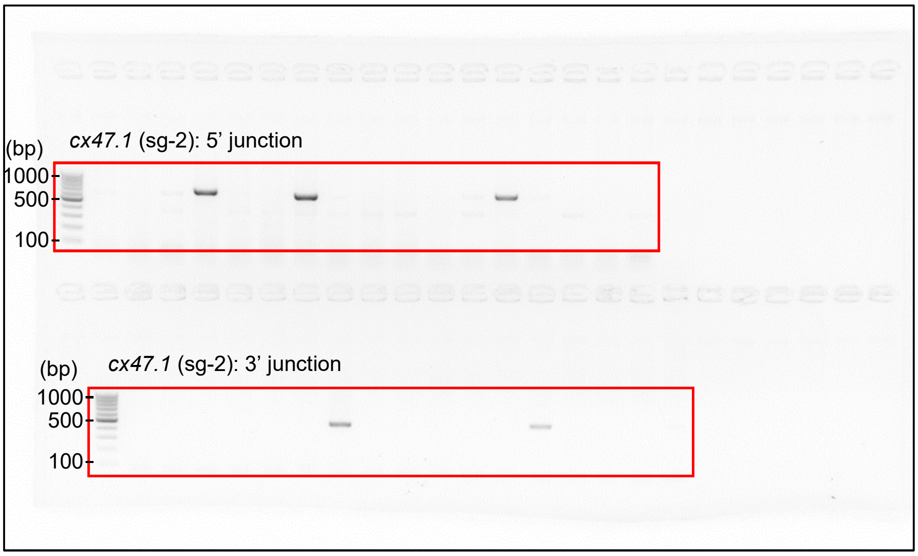

Supplementary Fig. 9f

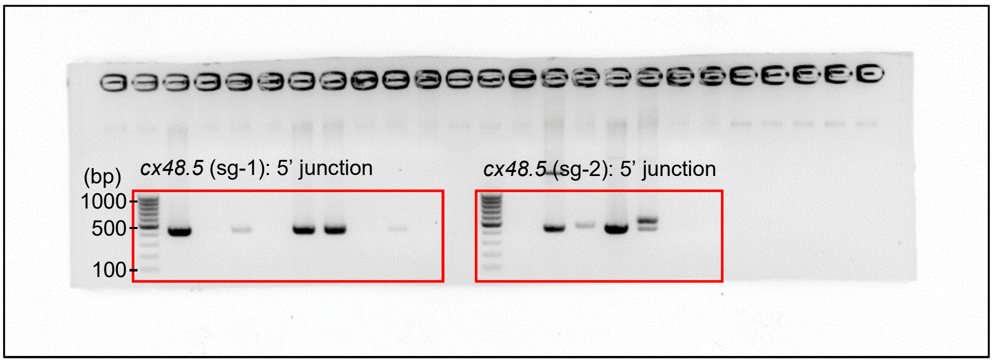

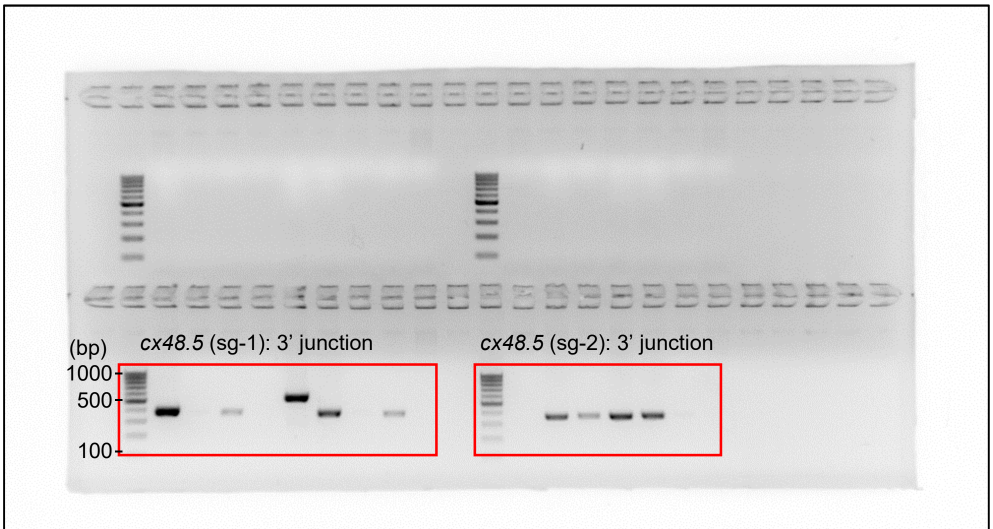

Supplementary Fig. 9g

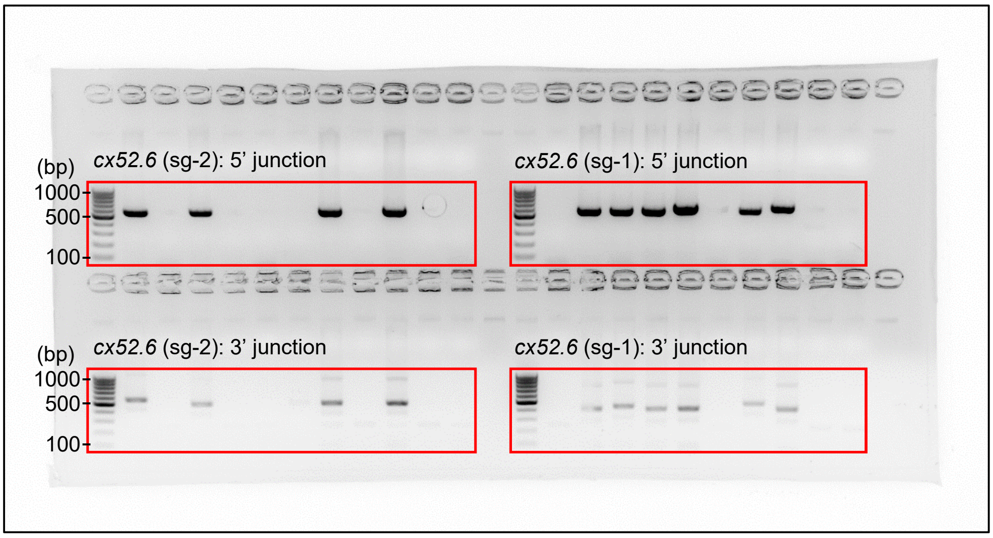

Supplementary Fig. 9h

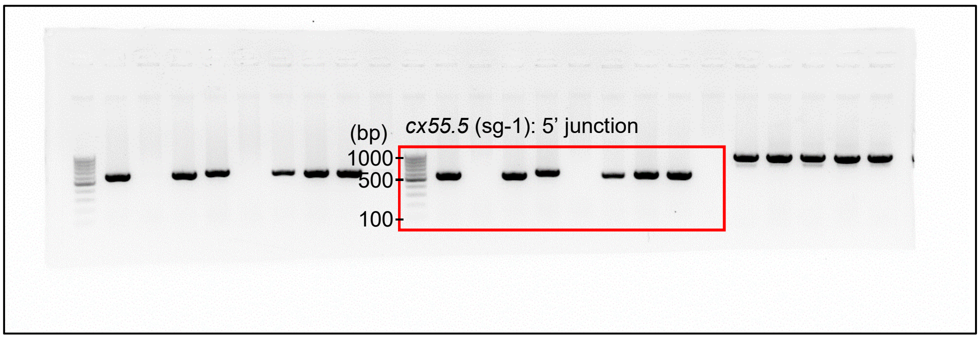

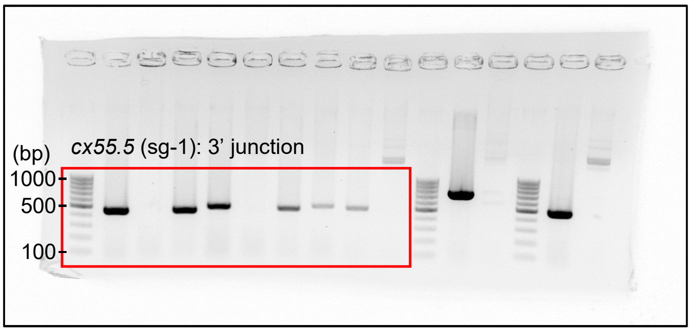

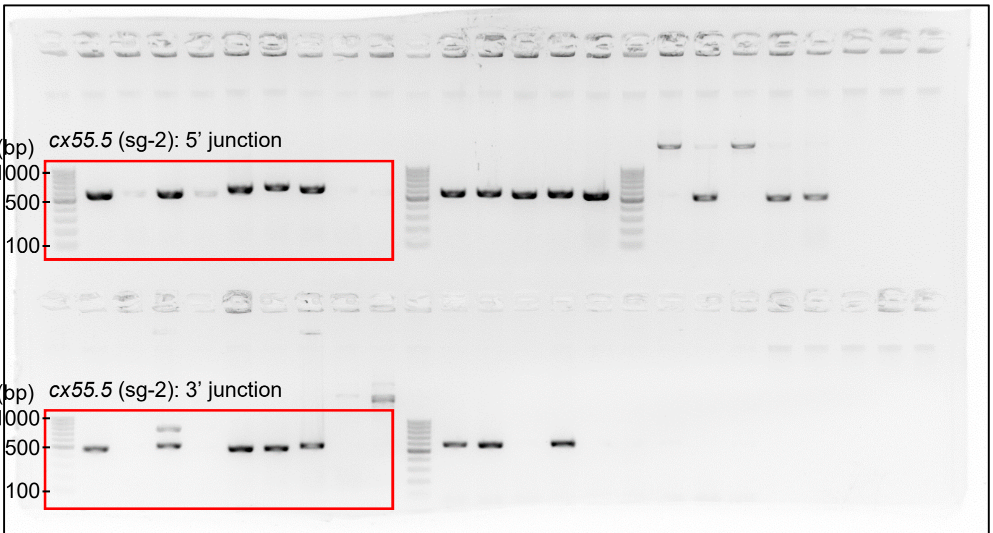

Supplementary Fig. 10b

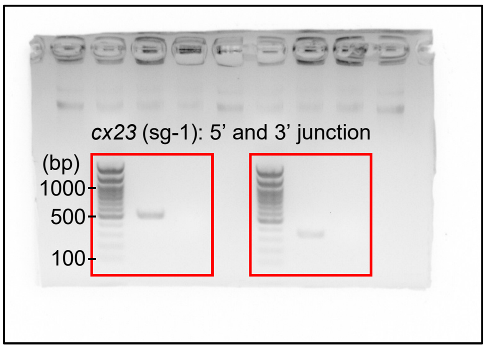

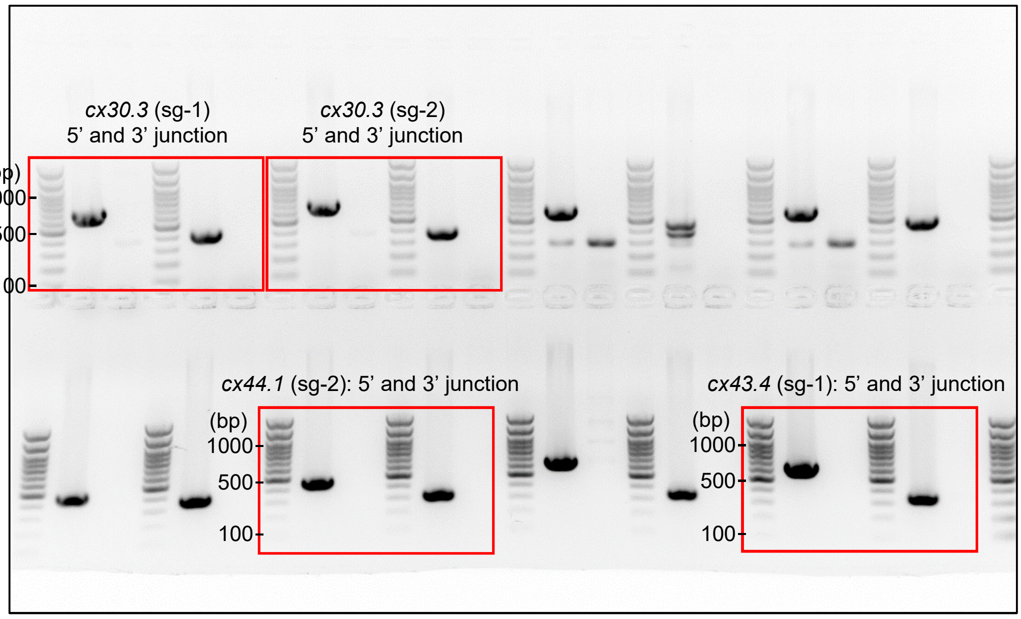

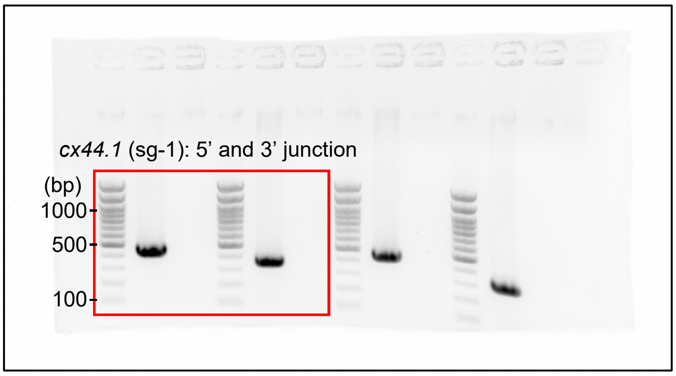

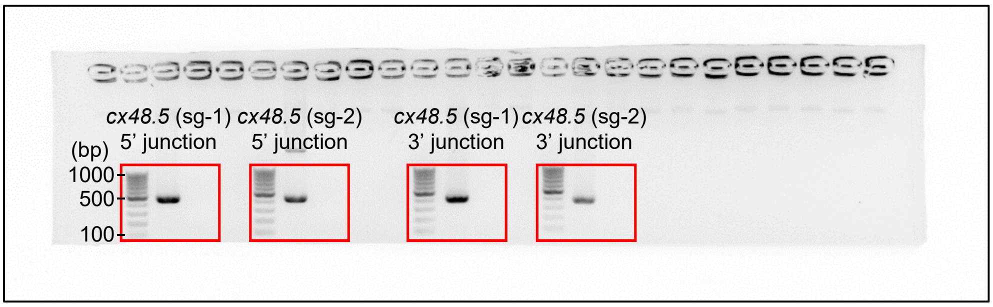

Supplementary Fig. 11b

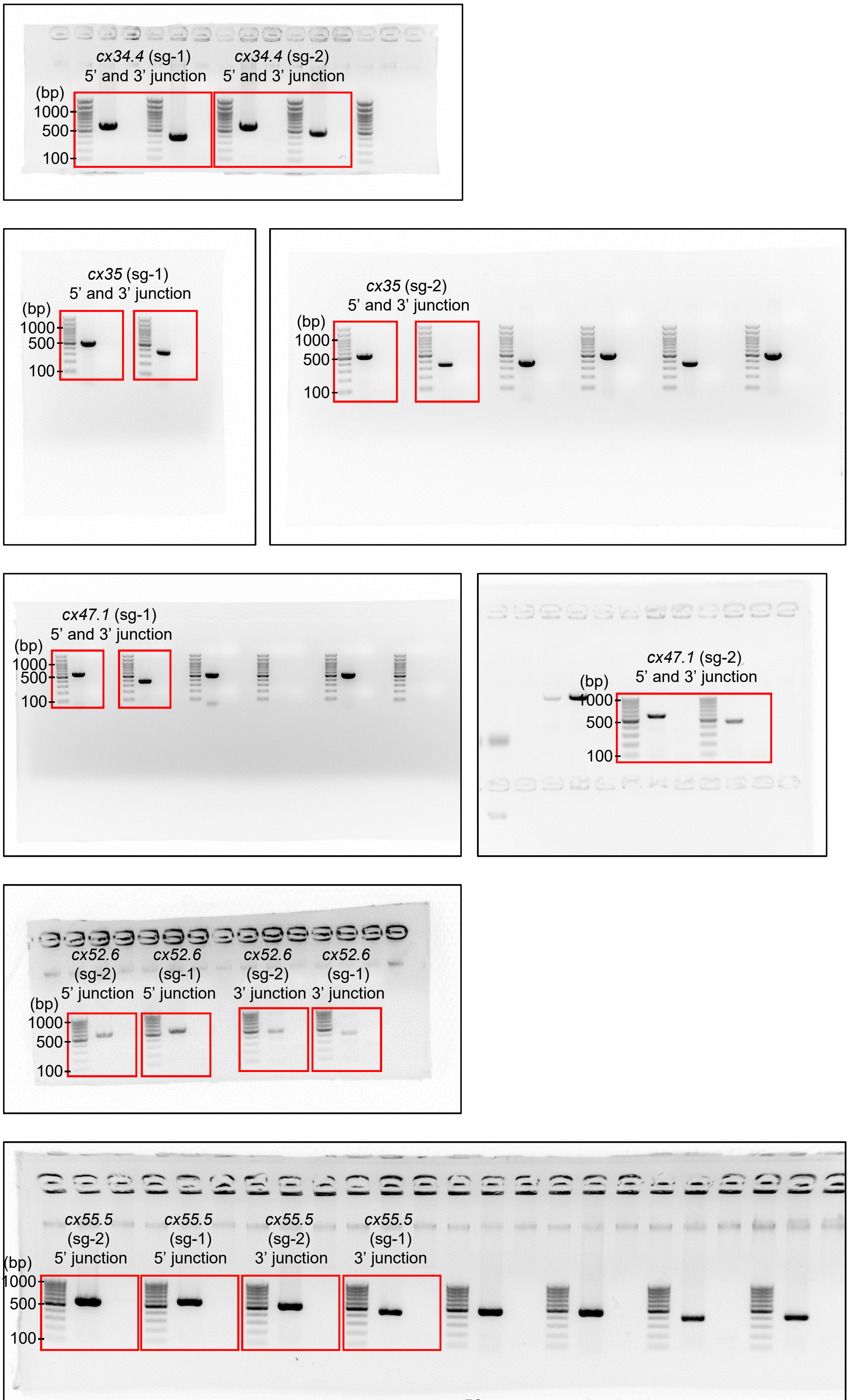

Supplementary Fig. 12a

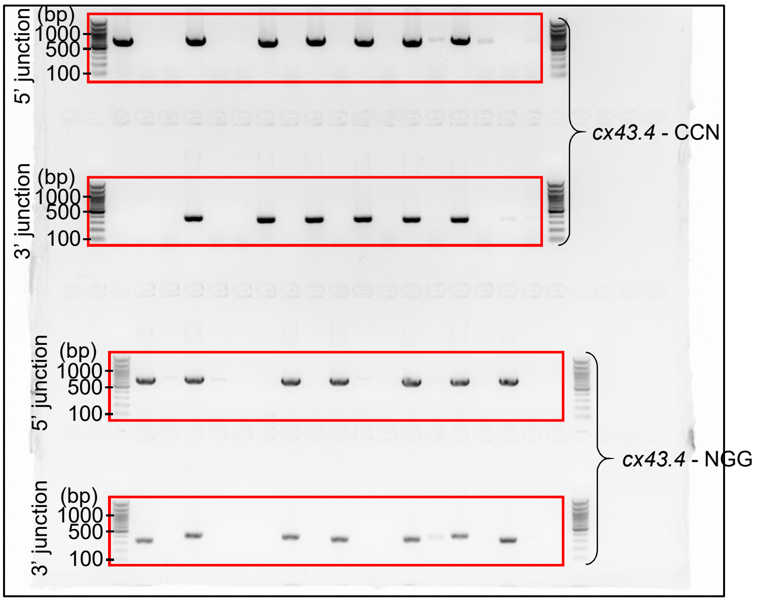

Supplementary Fig. 12b

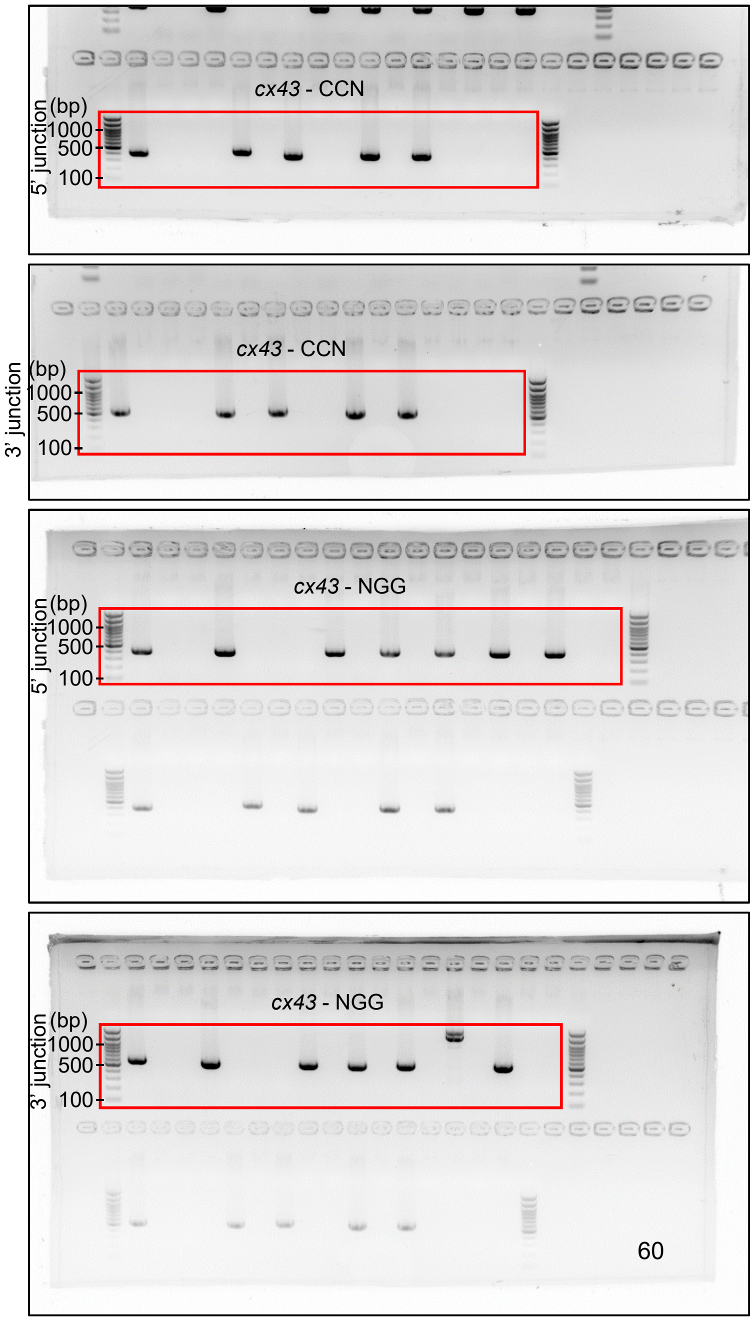

Supplementary Fig. 13

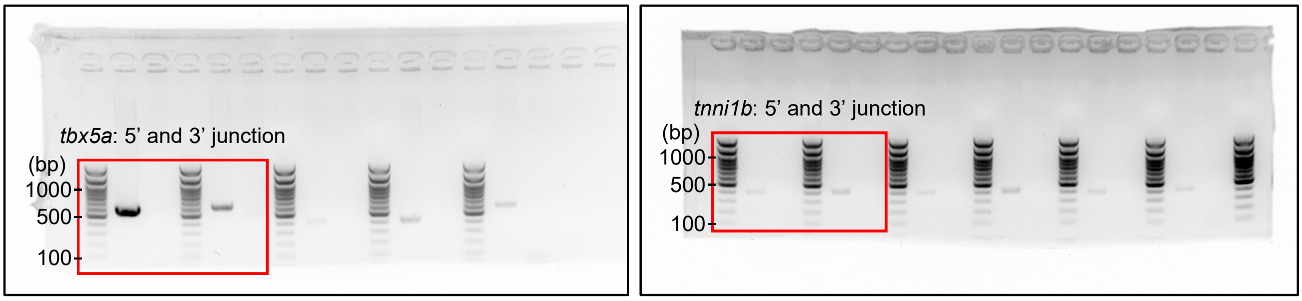

Supplementary Fig. 15b

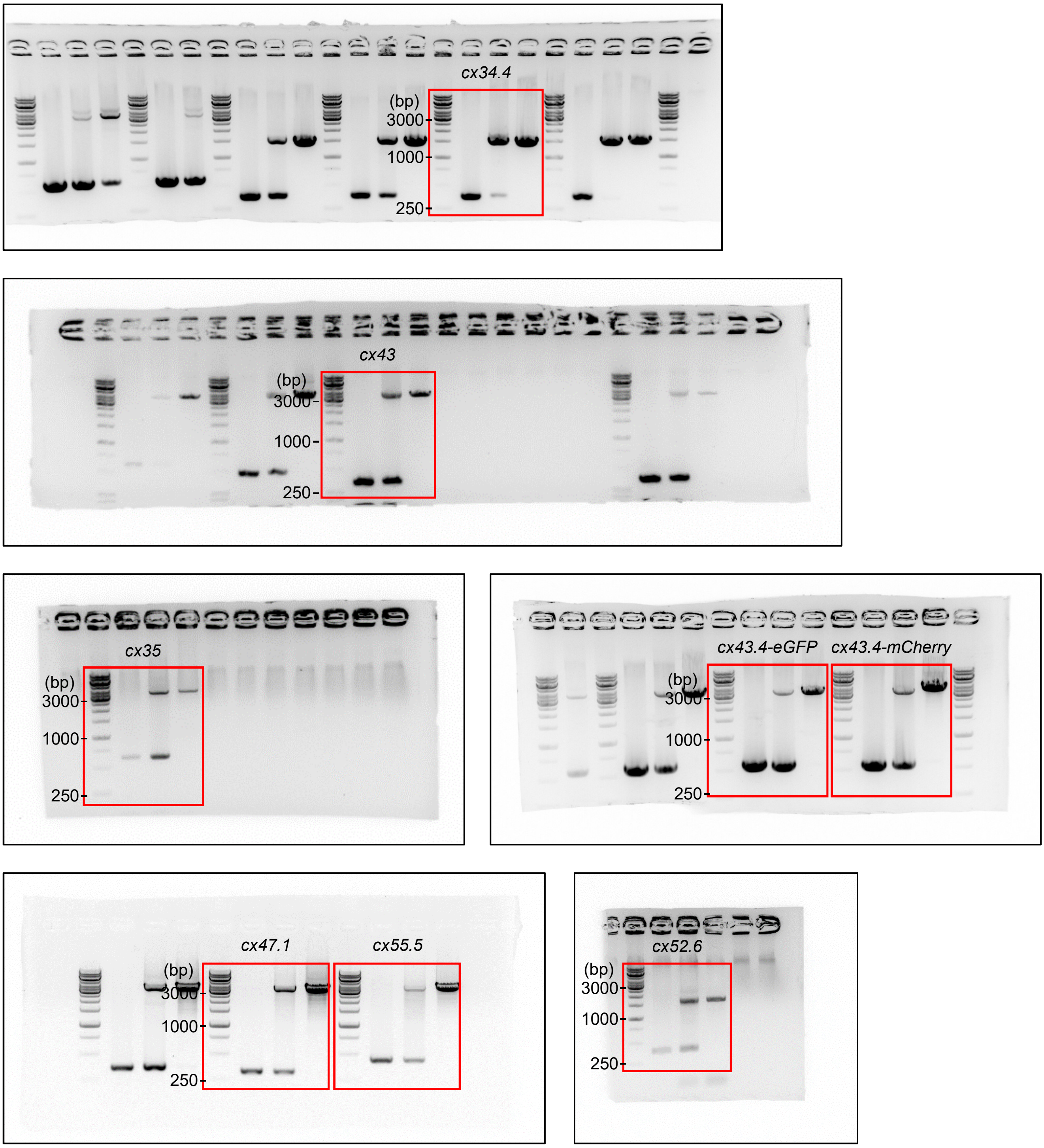

Supplementary Fig. 20

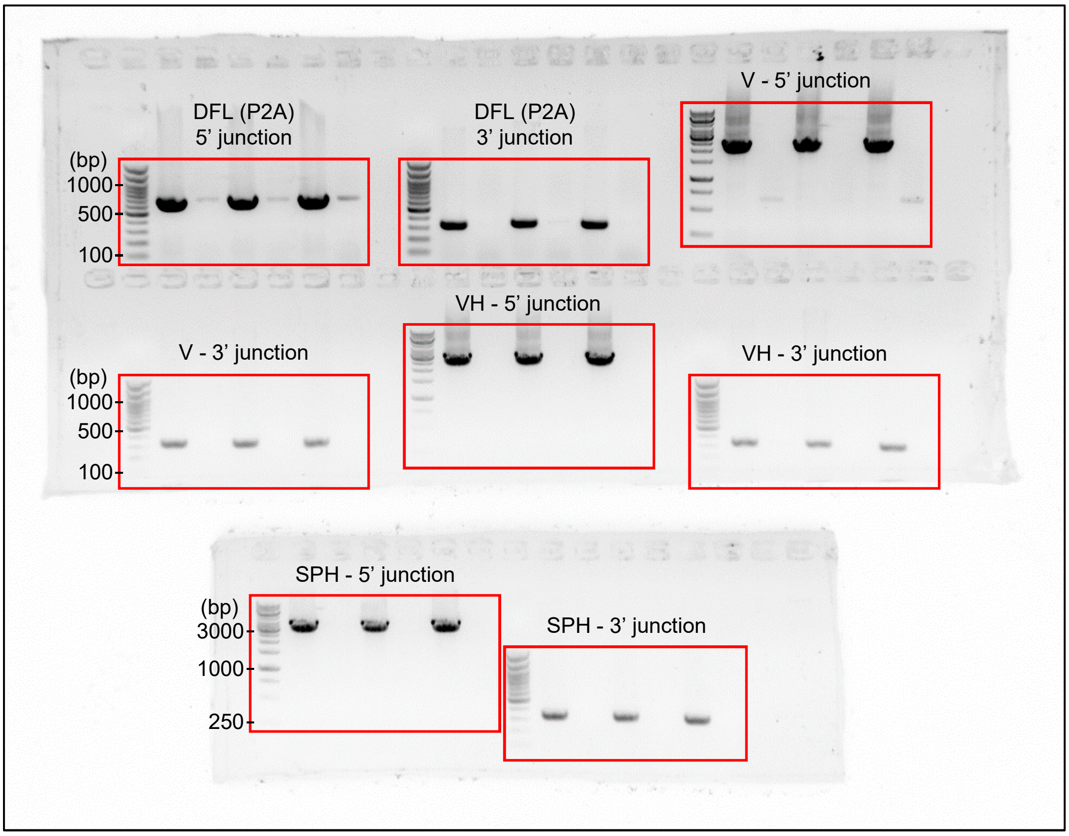

Supplementary Fig. 21

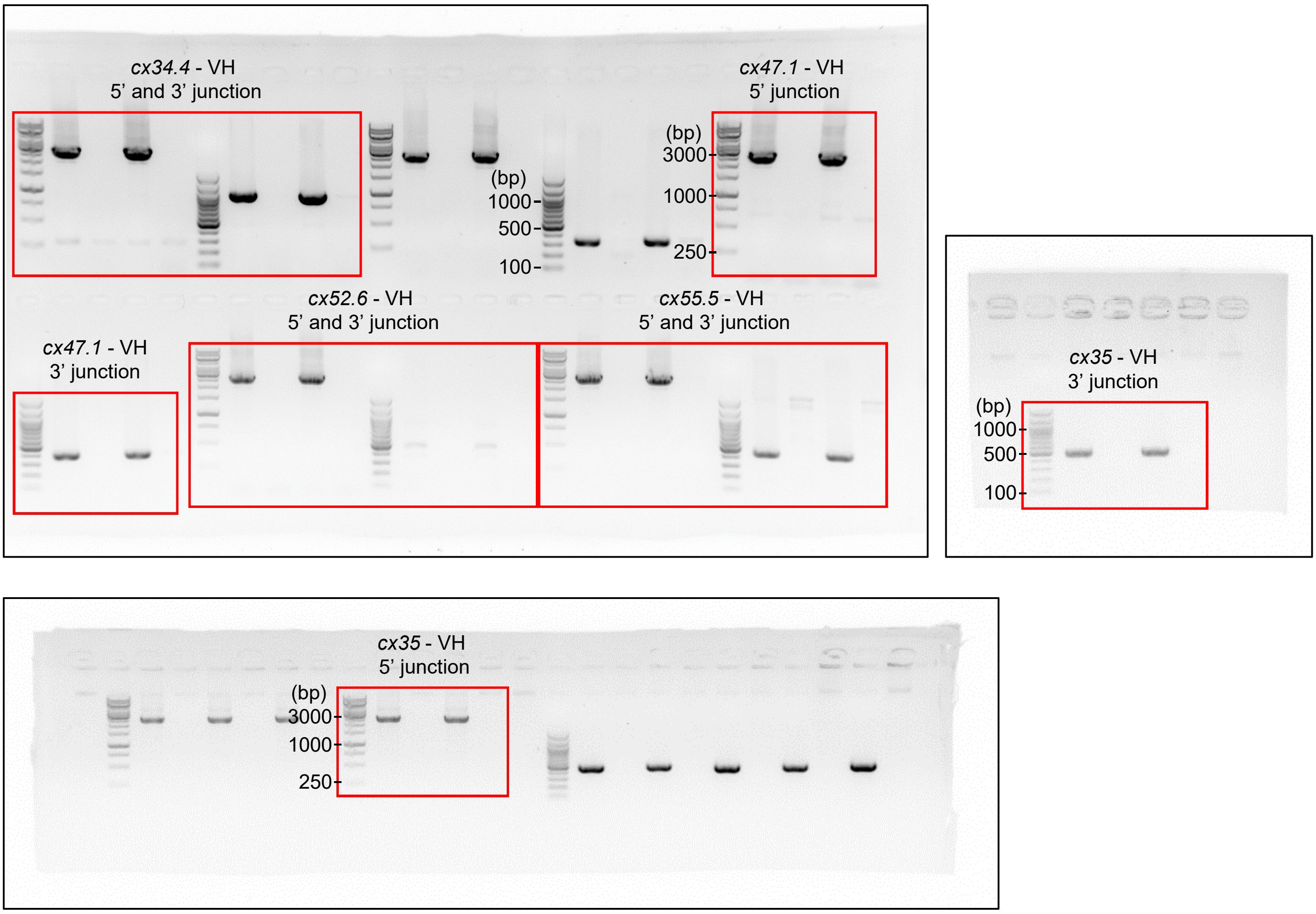

Supplementary Fig. 24

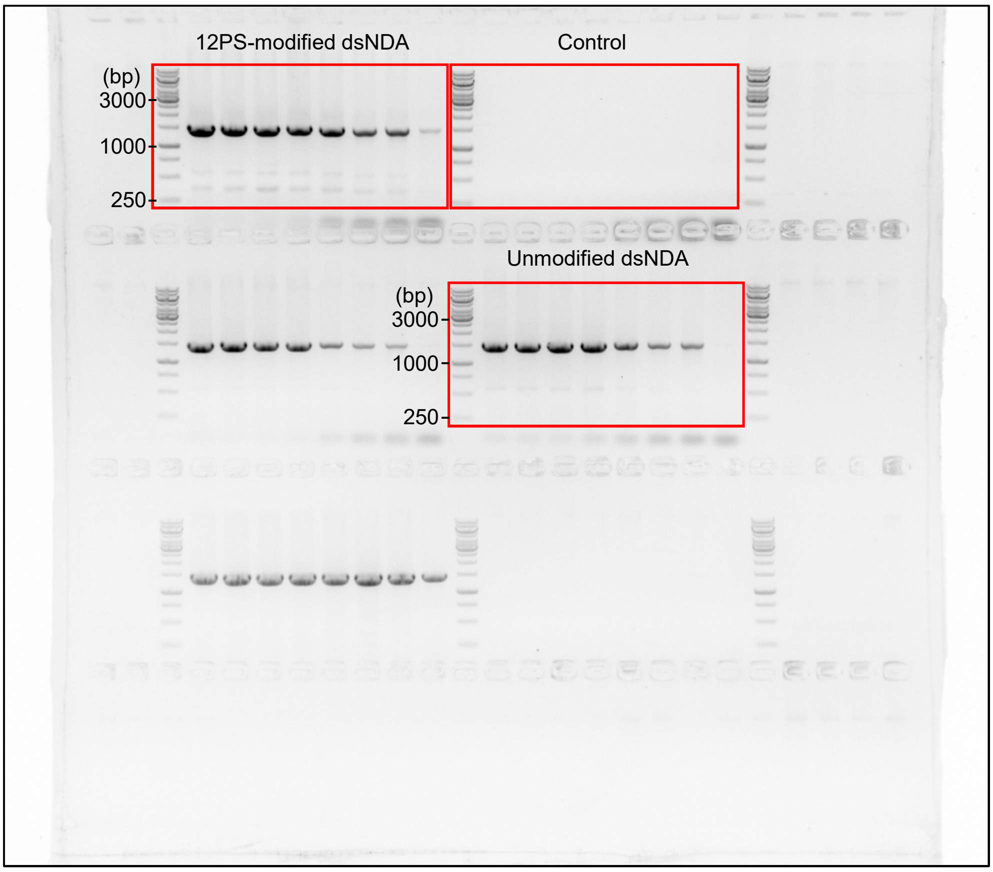

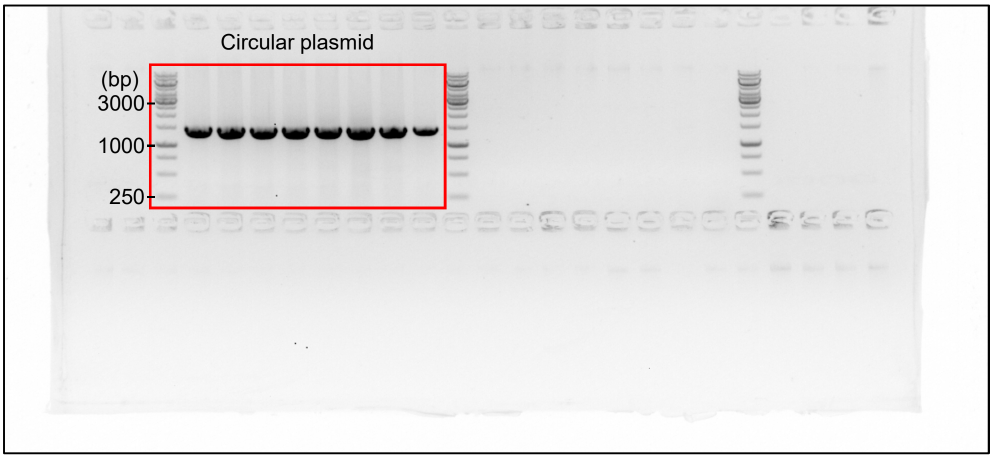

Supplementary Fig. 25b

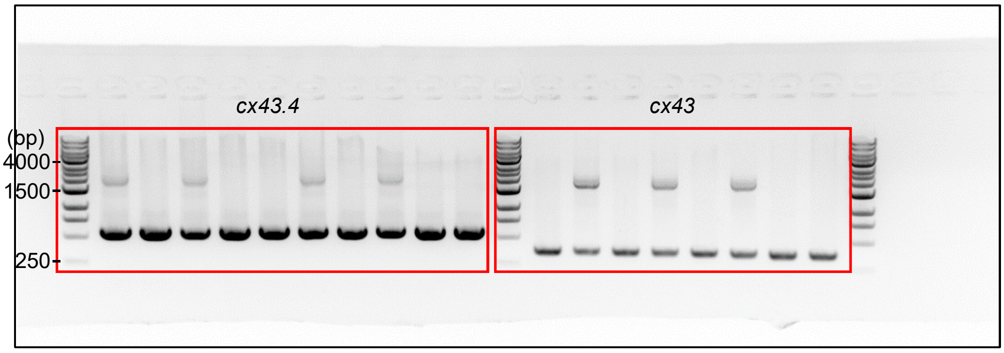

Supplementary Fig. 25d

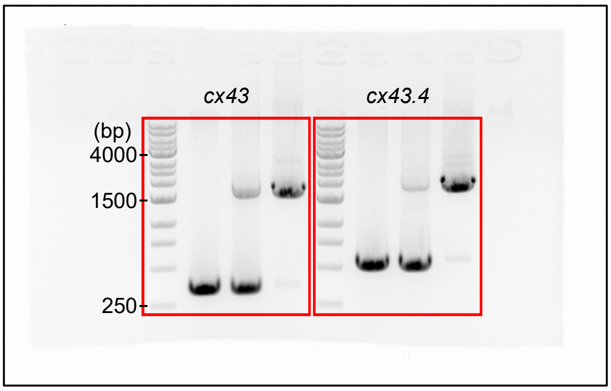

Supplementary Fig. 26a

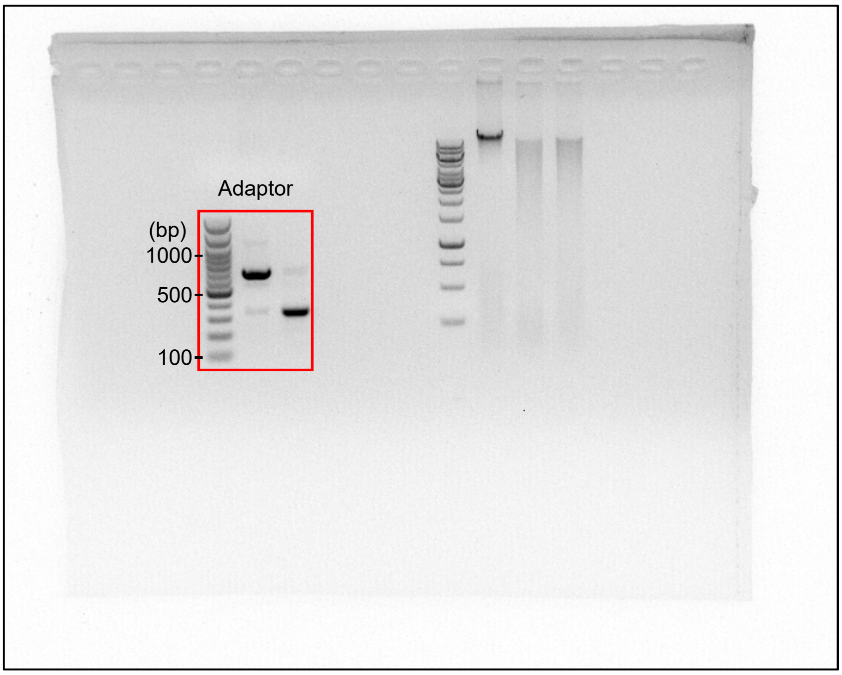

Supplementary Fig. 26b

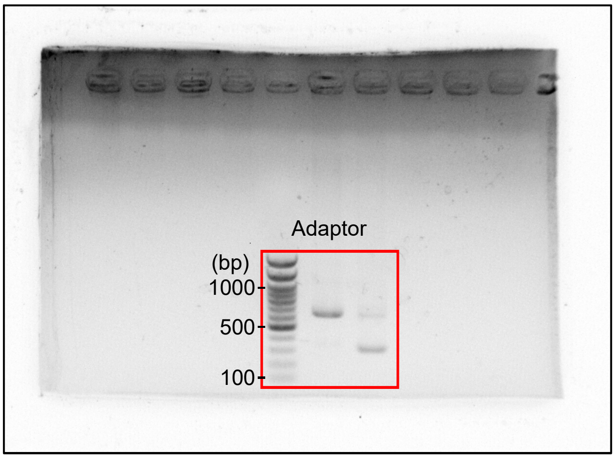

Supplementary Fig. 26c

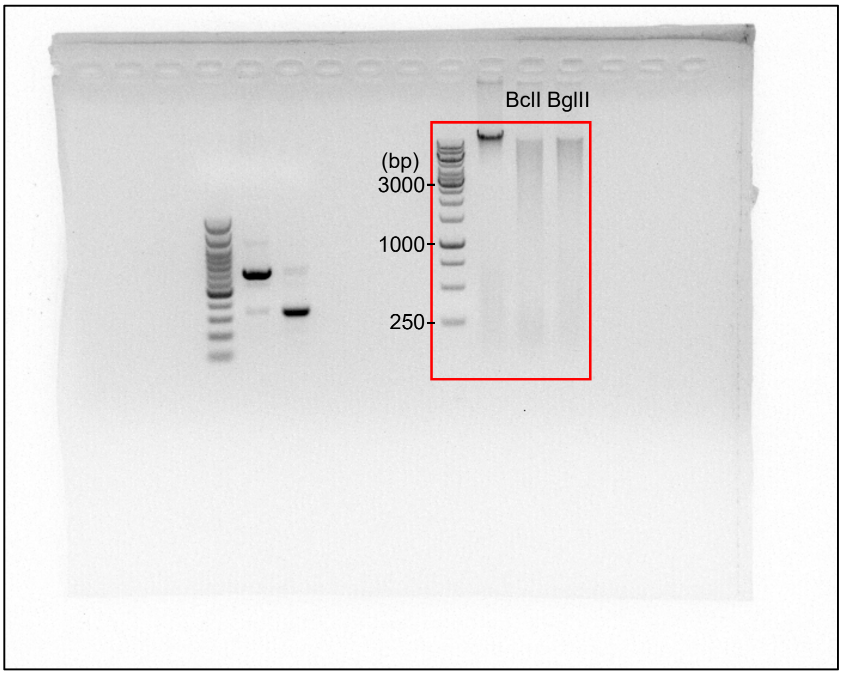

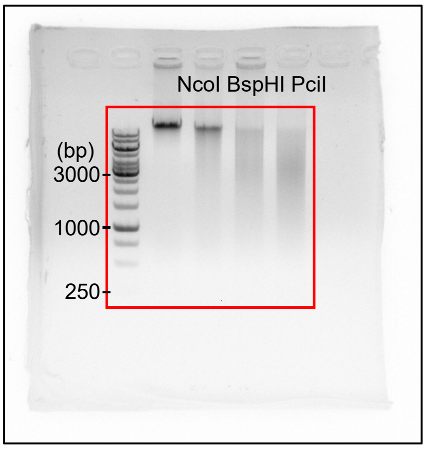

Supplementary Fig. 26d

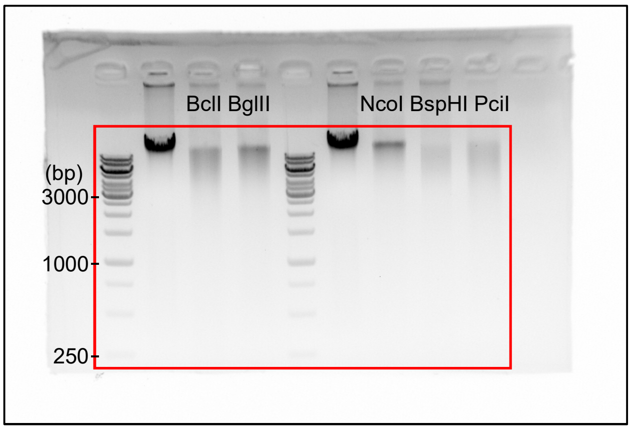

Supplementary Fig. 26e

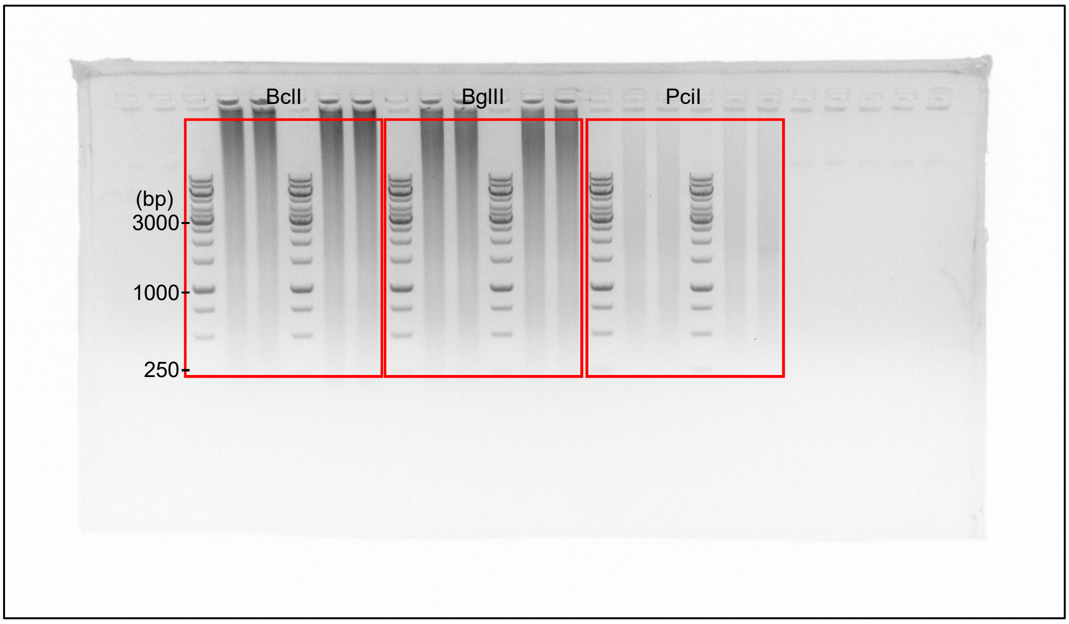

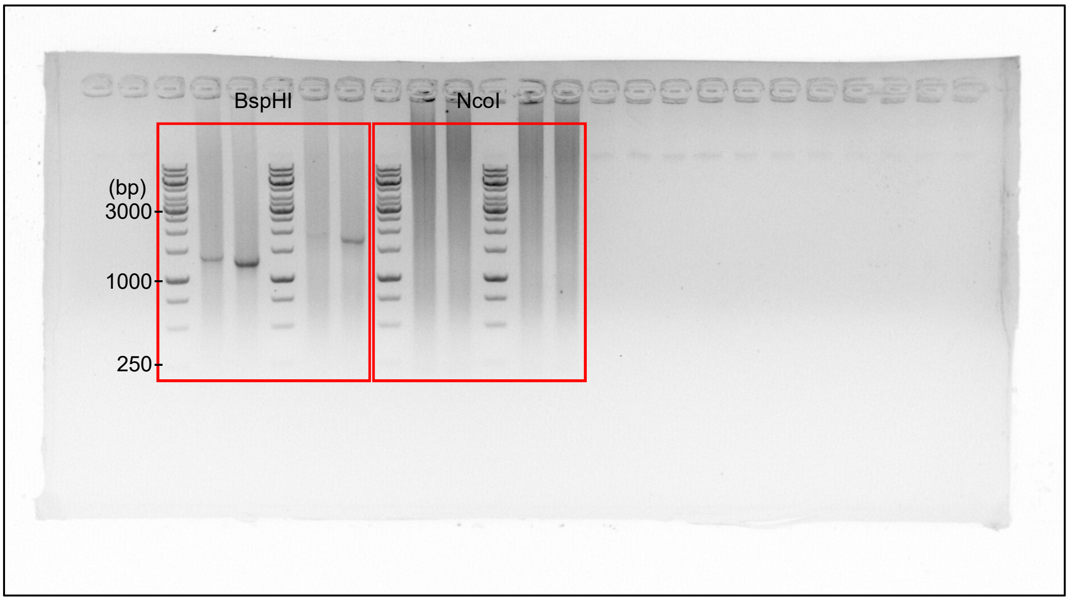

Supplementary Fig. 26f

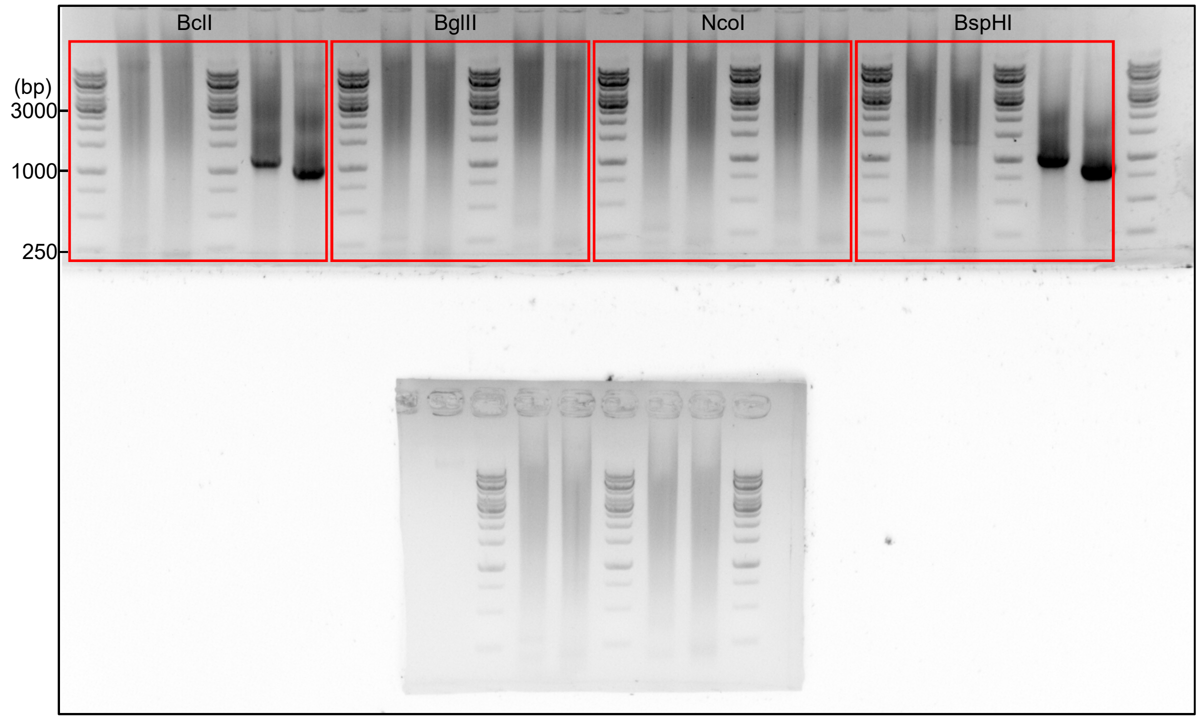

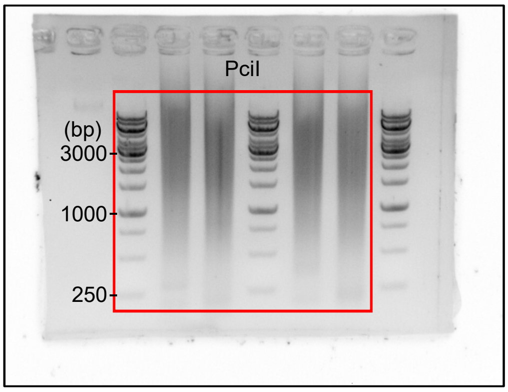

Supplementary Fig. 27c

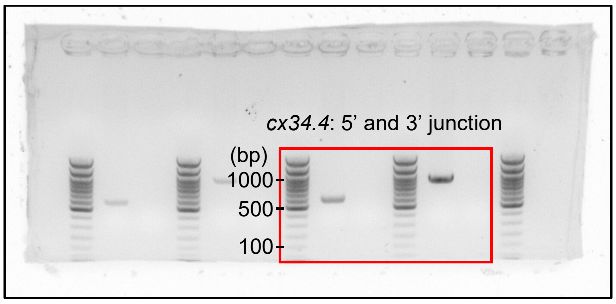

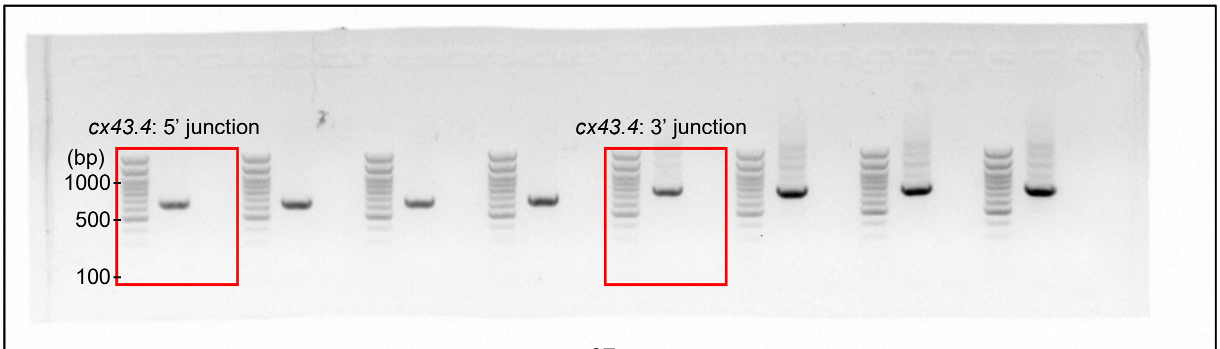

Supplement: Supplementary file 2 — Supplementary Information [file 42003_2023_5686_MOESM2_ESM.pdf]
