# Supplementary material for: Optimal tagging strategies for illuminating expression profiles of genes with different abundance in zebrafish
Source: Commun Biol. 2023 Dec 21;6:1300. doi: 10.1038/s42003-023-05686-1 (PMC10739737; doi:10.1038/s42003-023-05686-1)
Supplement: Supplementary file 3 — Description of Additional Supplementary Files [file 42003_2023_5686_MOESM3_ESM.pdf]

Description of Additional Supplementary Files

File name: Supplementary Data 1
Description: Results of the 5'-junction PCR of F_0 embryos for *cx30.3*, *cx34.4*, *cx35*, *cx44.1*, *cx47.1*, *cx48.5*, *cx52.6*, and *cx55.5*.

File name: Supplementary Data 2
Description: Plasmid sequences involved in this work.

File name: Supplementary Data 3
Description: sgRNA sequences and PCR primer sequences involved in this work.

File name: Supplementary Data 4
Description: Source Data for the graphs presented in this work.

File name: Supplementary Data 5
Description: Source Data for the tested founder fish (Fig. 1e, 2d, and 5d) and the mosaicism of each founder fish (Fig. 1g, 2e, and Supplementary Fig. 25c).

File name: Supplementary Data 6
Description: Chemical structures involved in Supplementary Fig. 23.
