# Supplementary material for: Optimal tagging strategies for illuminating expression profiles of genes with different abundance in zebrafish
Source: Commun Biol. 2023 Dec 21;6:1300. doi: 10.1038/s42003-023-05686-1 (PMC10739737; doi:10.1038/s42003-023-05686-1)
Supplement: Supplementary file 4 — Supplementary Data 1 [file 42003_2023_5686_MOESM4_ESM.pdf]

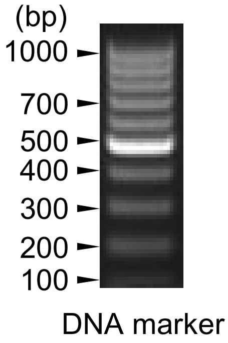

The left DNA marker was used in all figures of this experiment

Genomic DNA was isolated from caudal fins of 1-month-old F_0 zebrafish and used for 5’ junction PCR. Numbers in red represent F_0 zebrafish harboring the target band.

cx30.3-sg-1

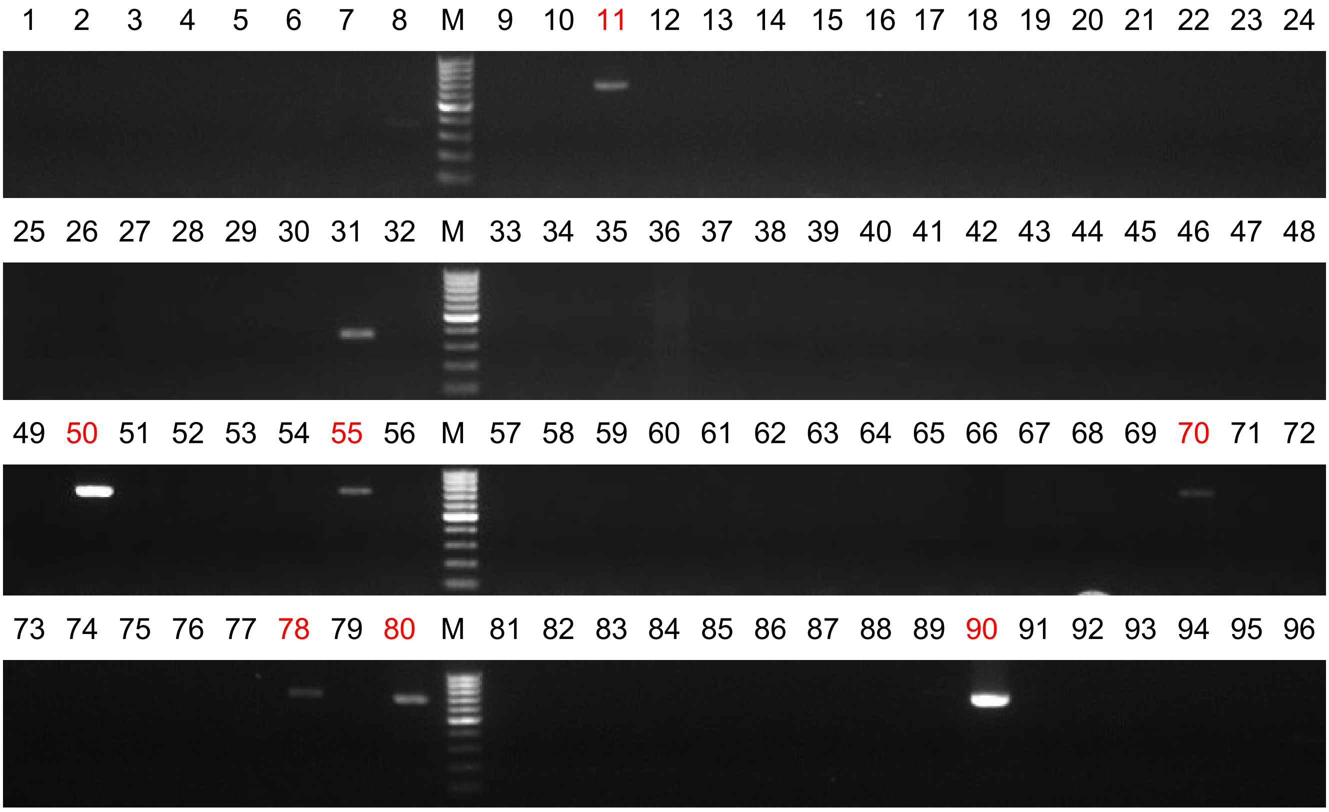

target band – 672 bp

cx30.3-sg-2

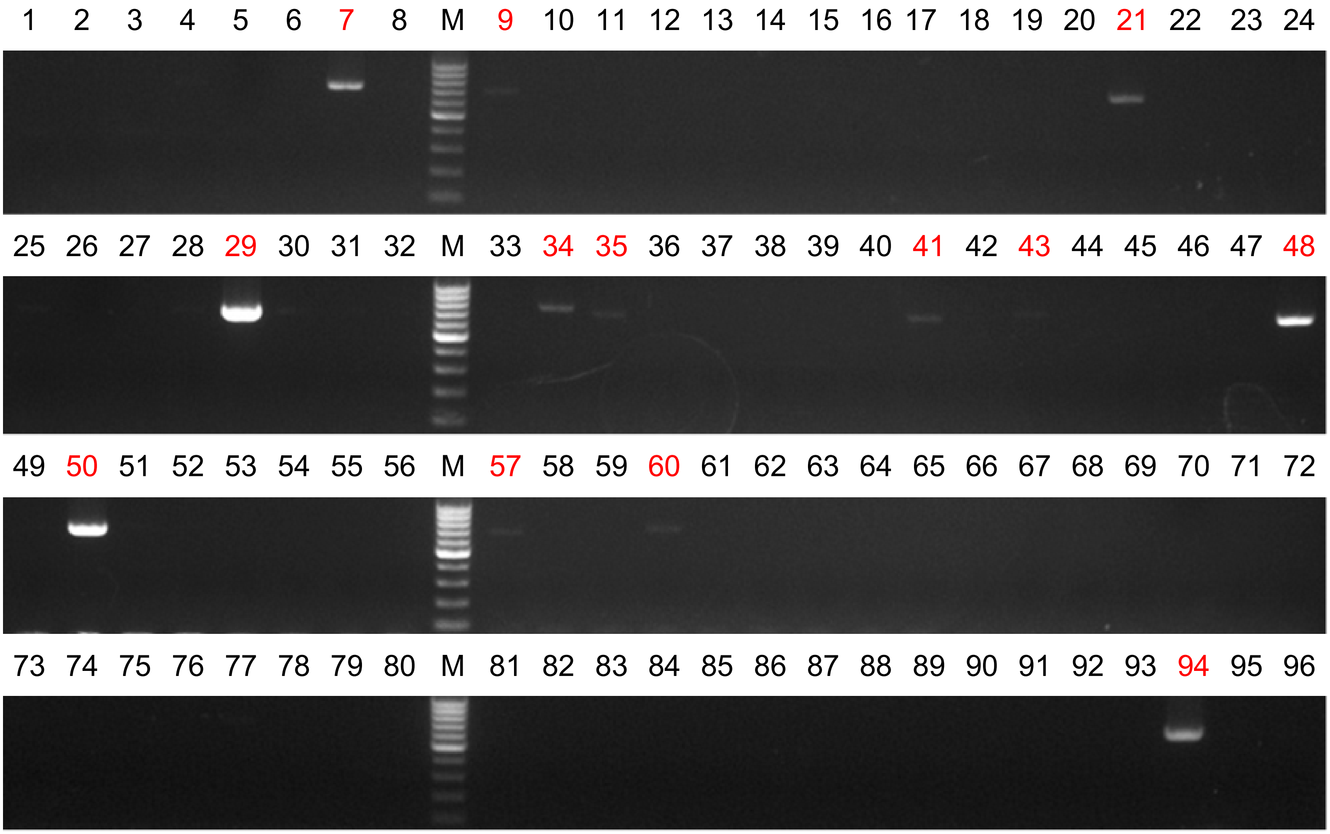

target band – 672 bp

cx34.4-sg-1

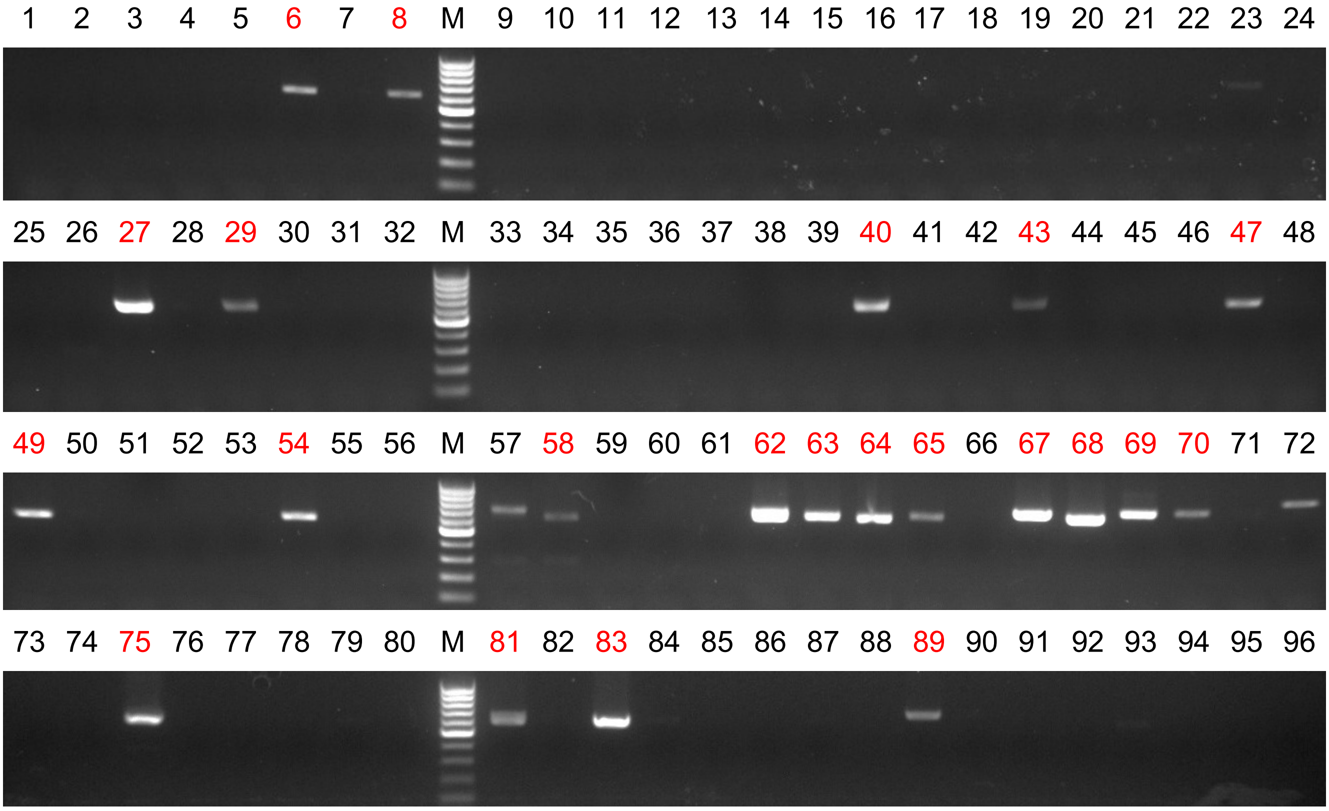

target band – 588 bp

cx34.4-sg-2

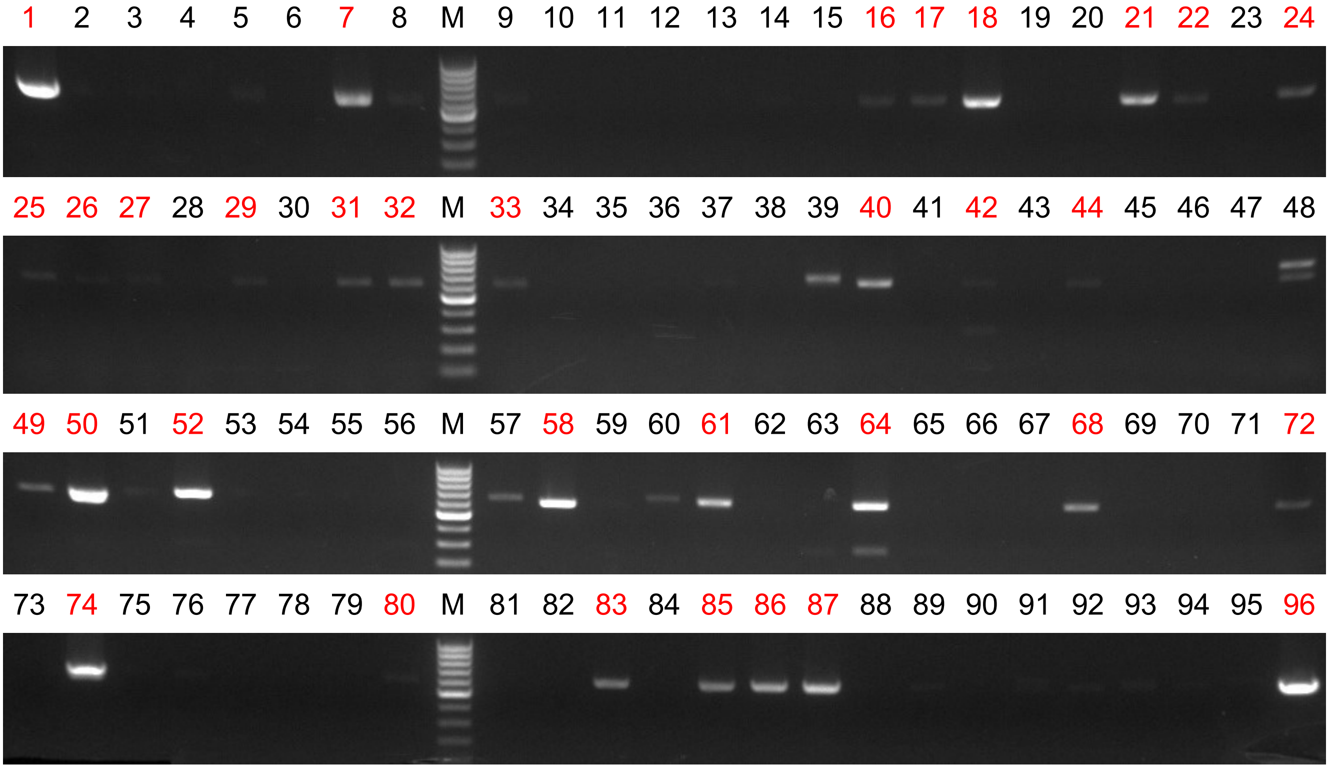

target band – 588 bp

cx35-sg-1

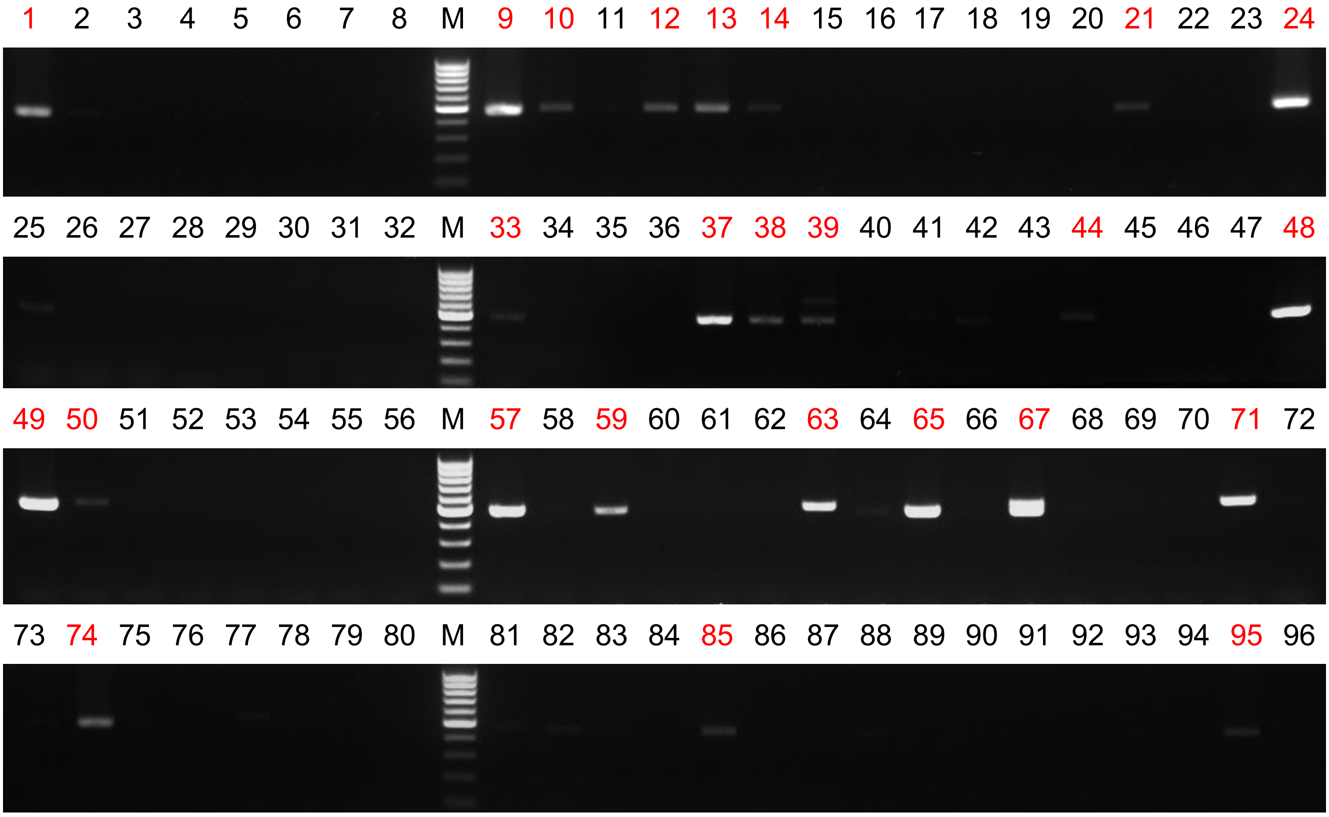

target band – 586 bp

cx35-sg-2

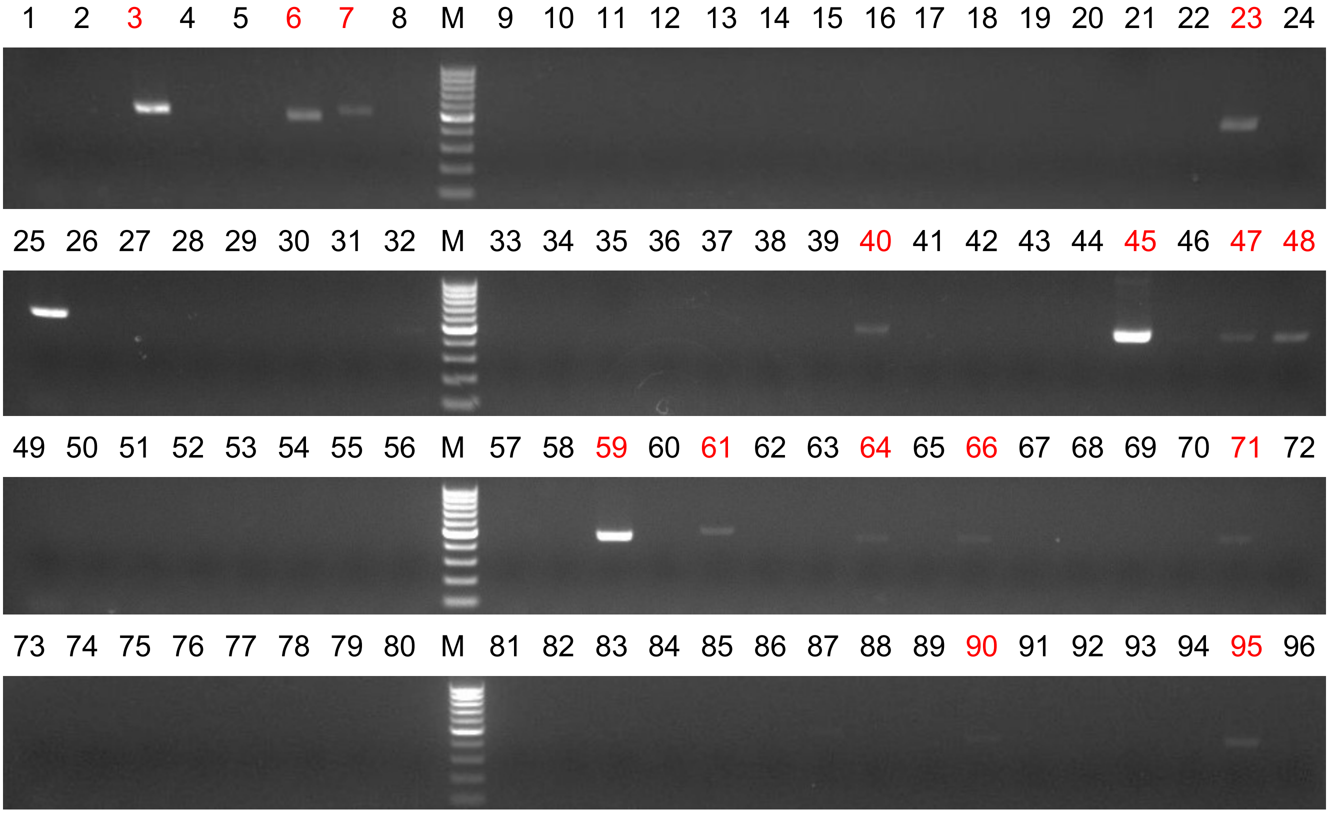

target band – 586 bp

cx44.1-sg-1

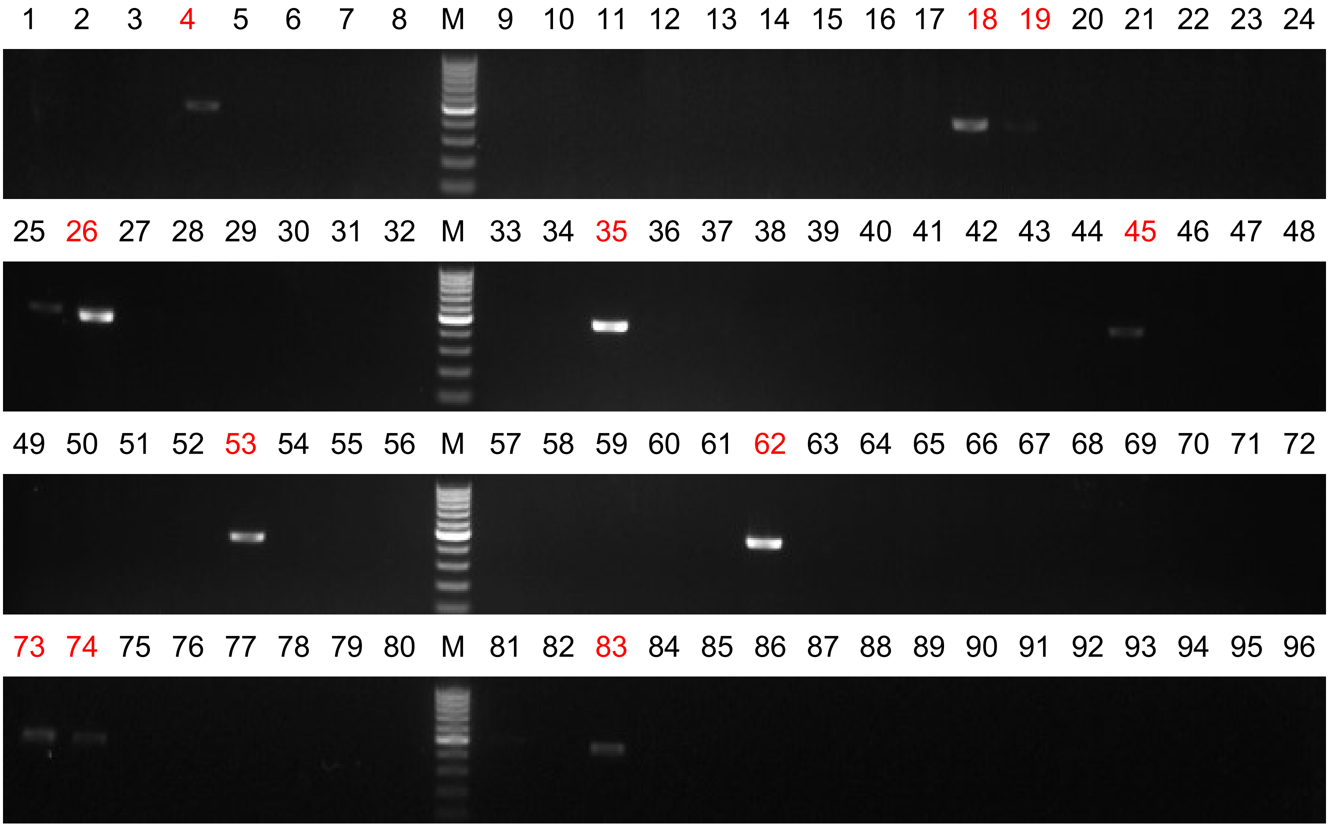

target band – 453 bp

cx44.1-sg-2

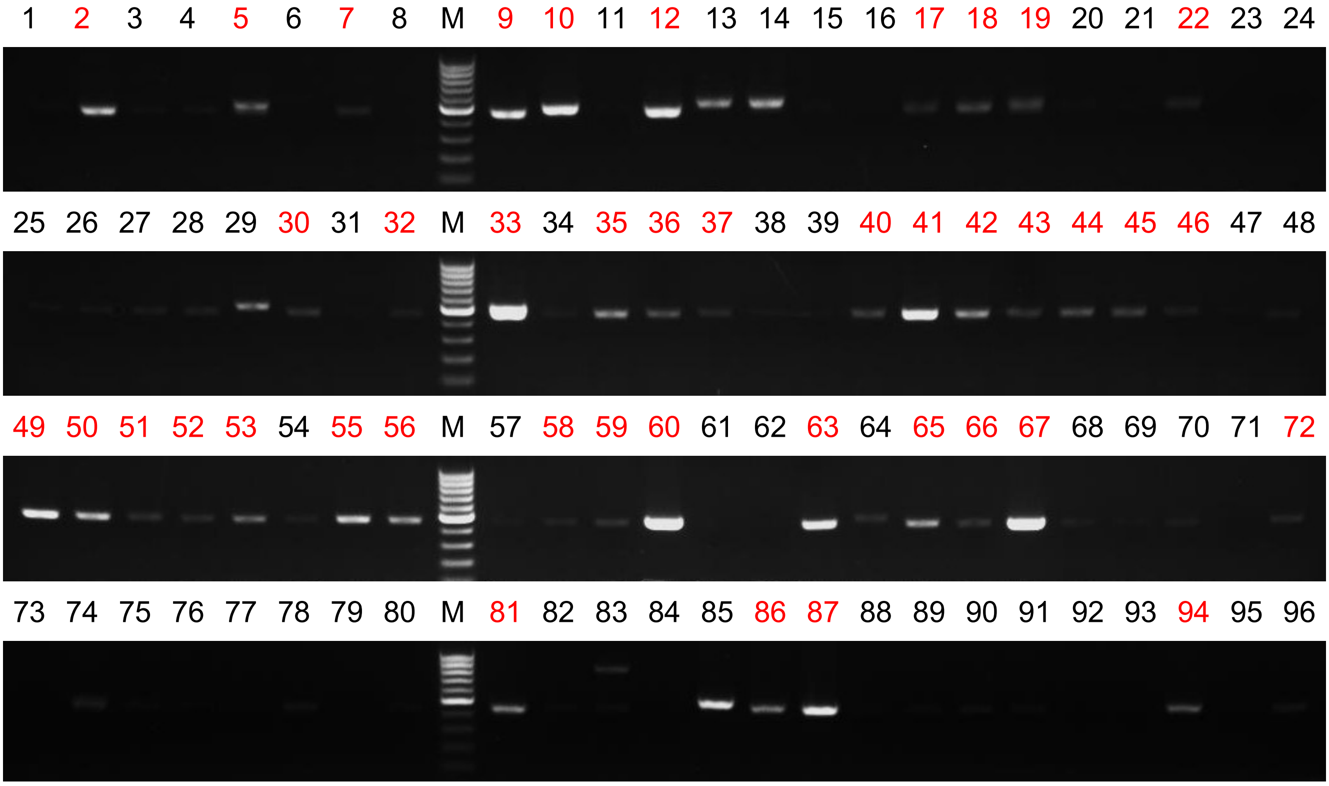

target band – 453 bp

cx47.1-sg-1

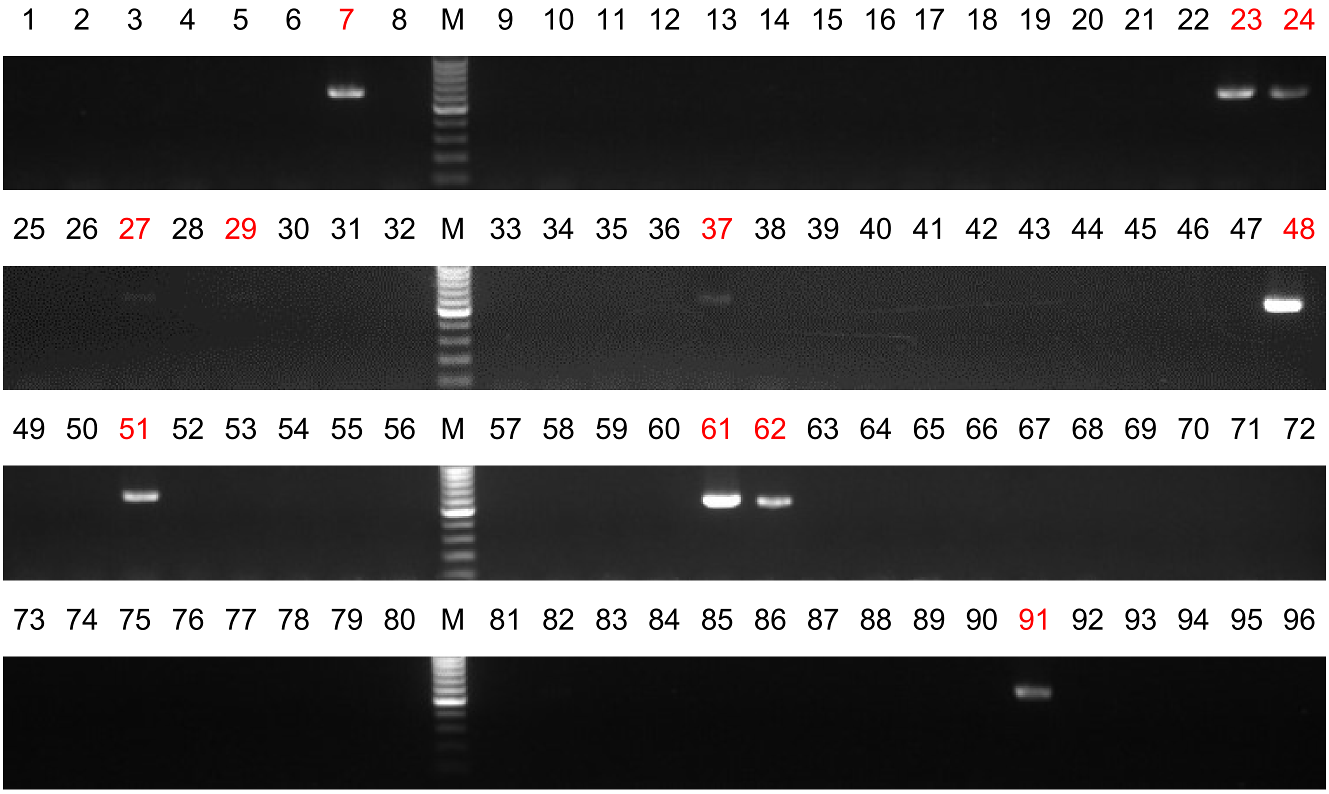

target band – 584 bp

cx47.1-sg-2

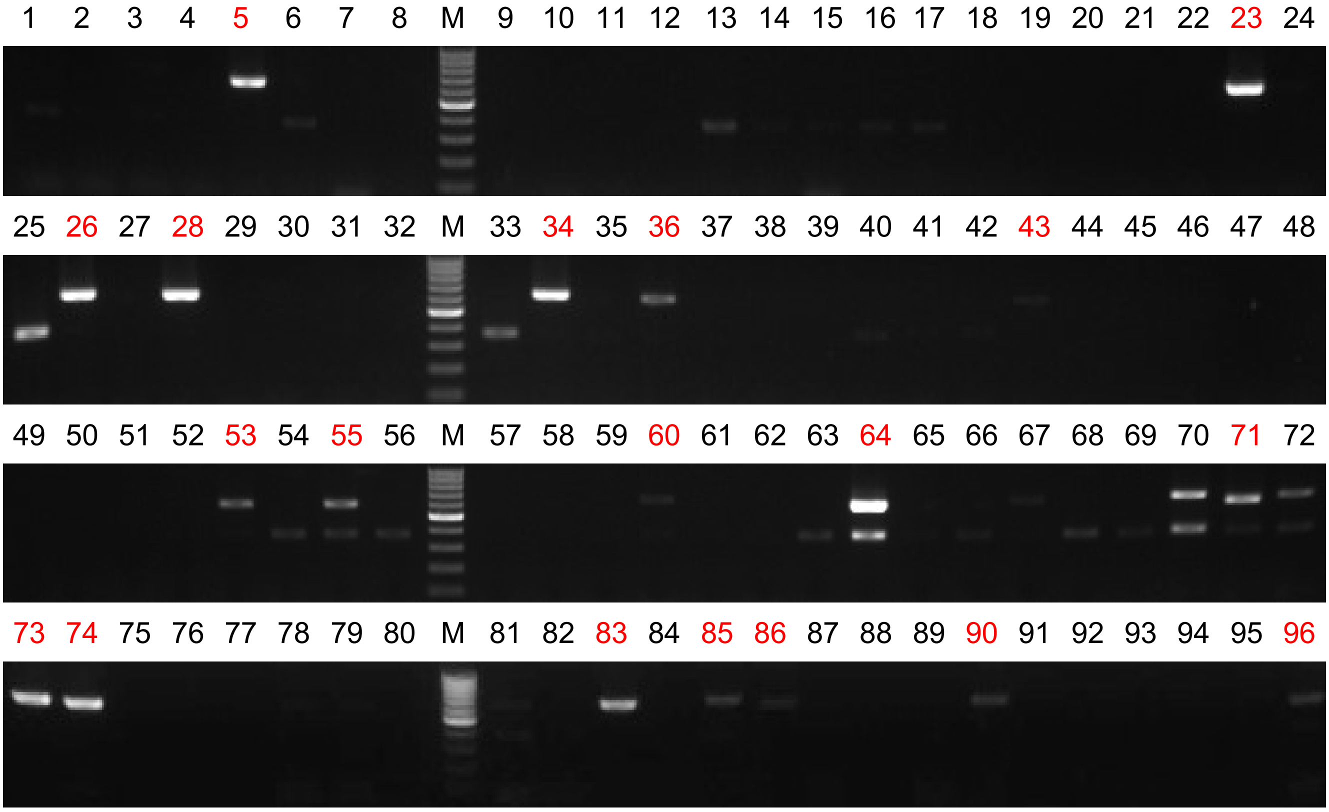

target band – 584 bp

cx48.5-sg-1

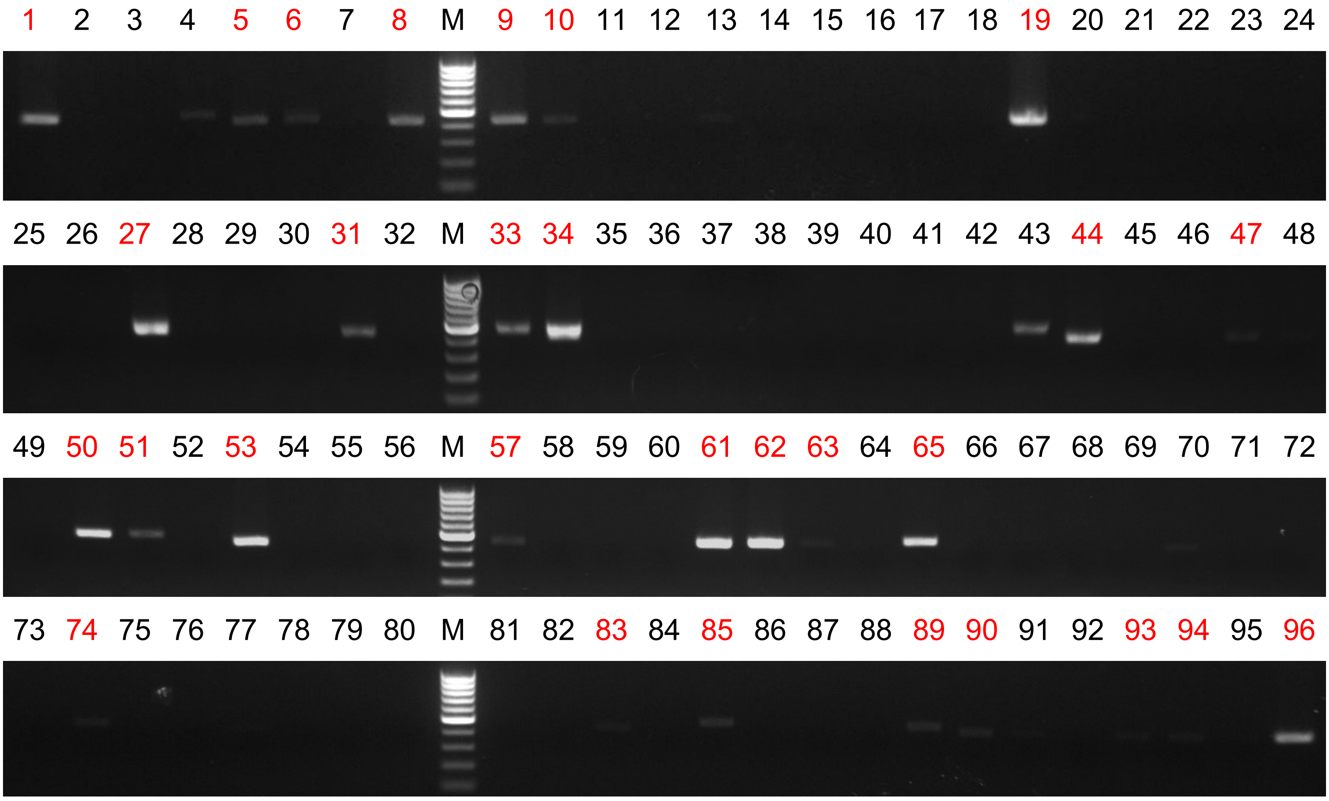

target band – 506 bp

cx48.5-sg-2

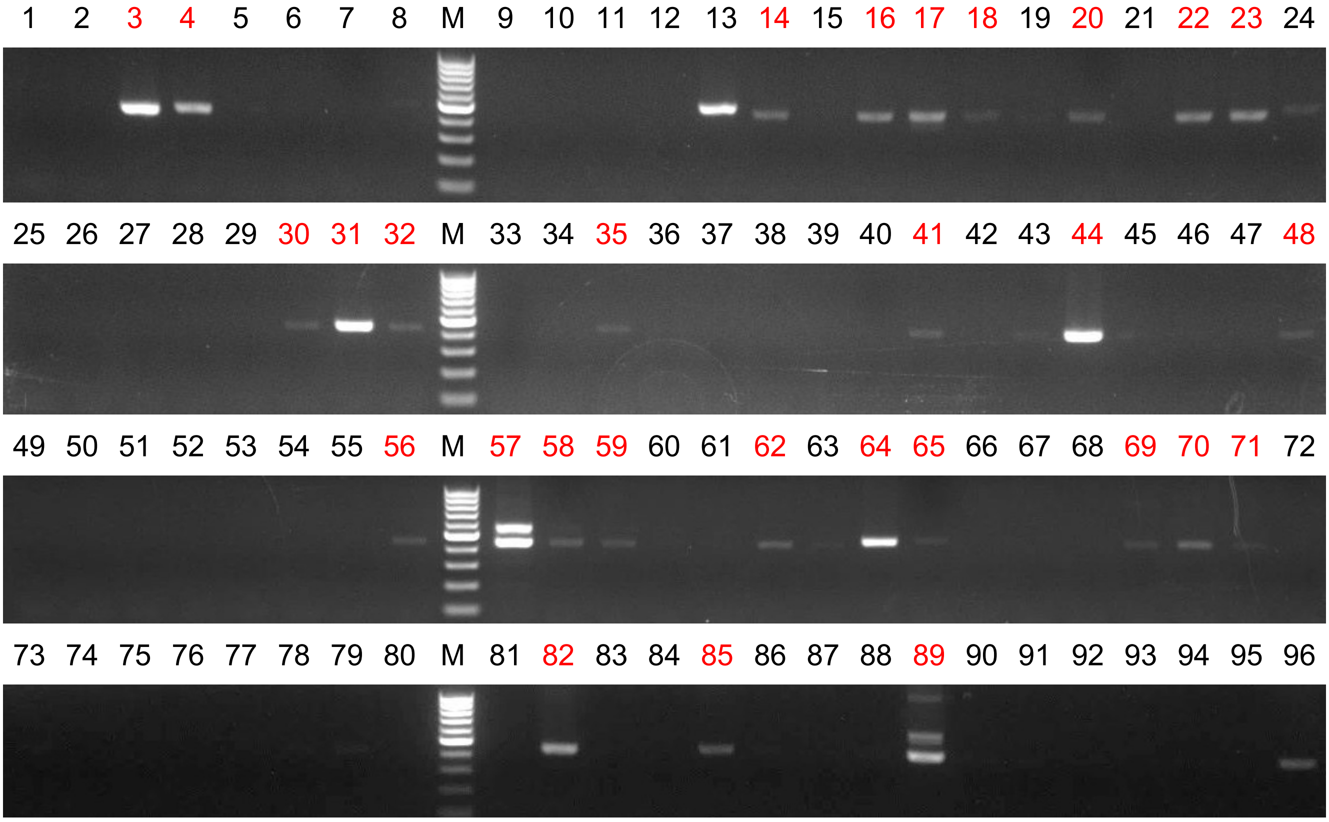

target band – 506 bp

cx52.6-sg-1

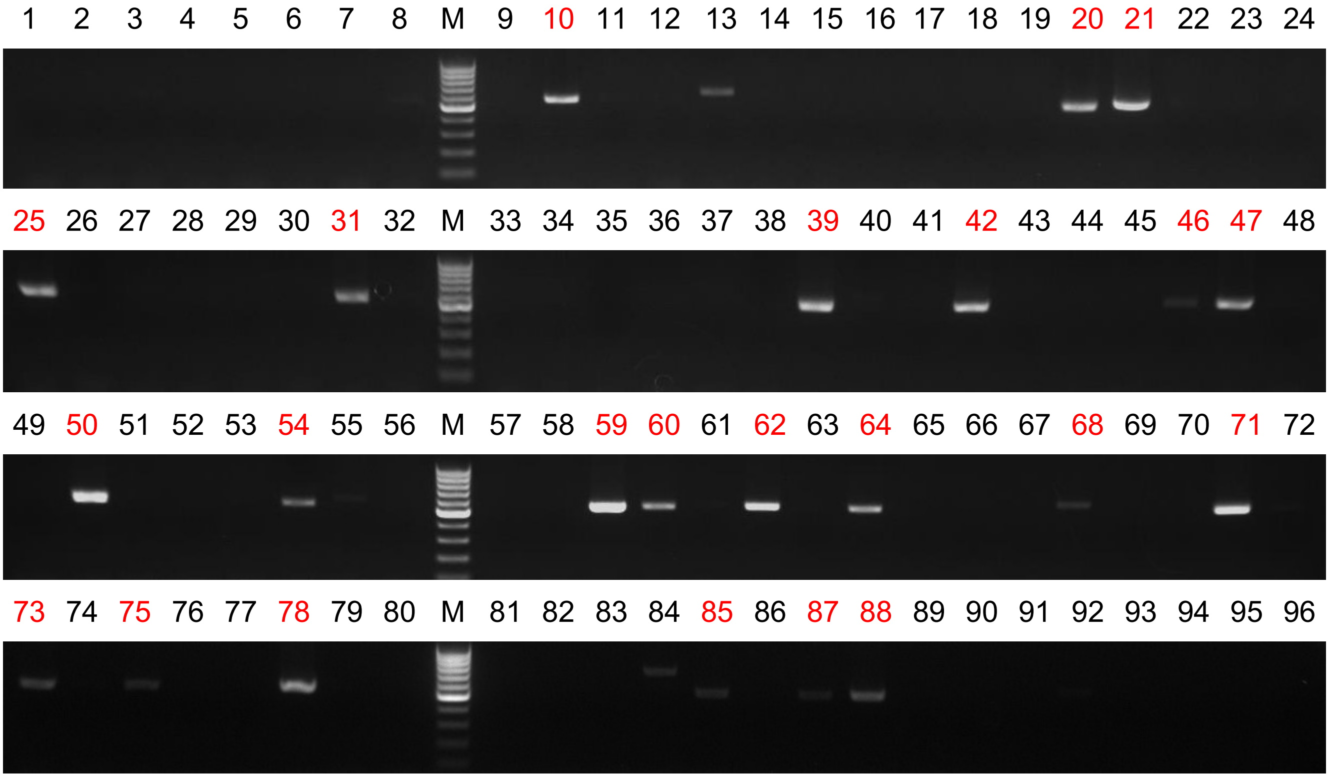

target band – 543 bp

cx52.6-sg-2

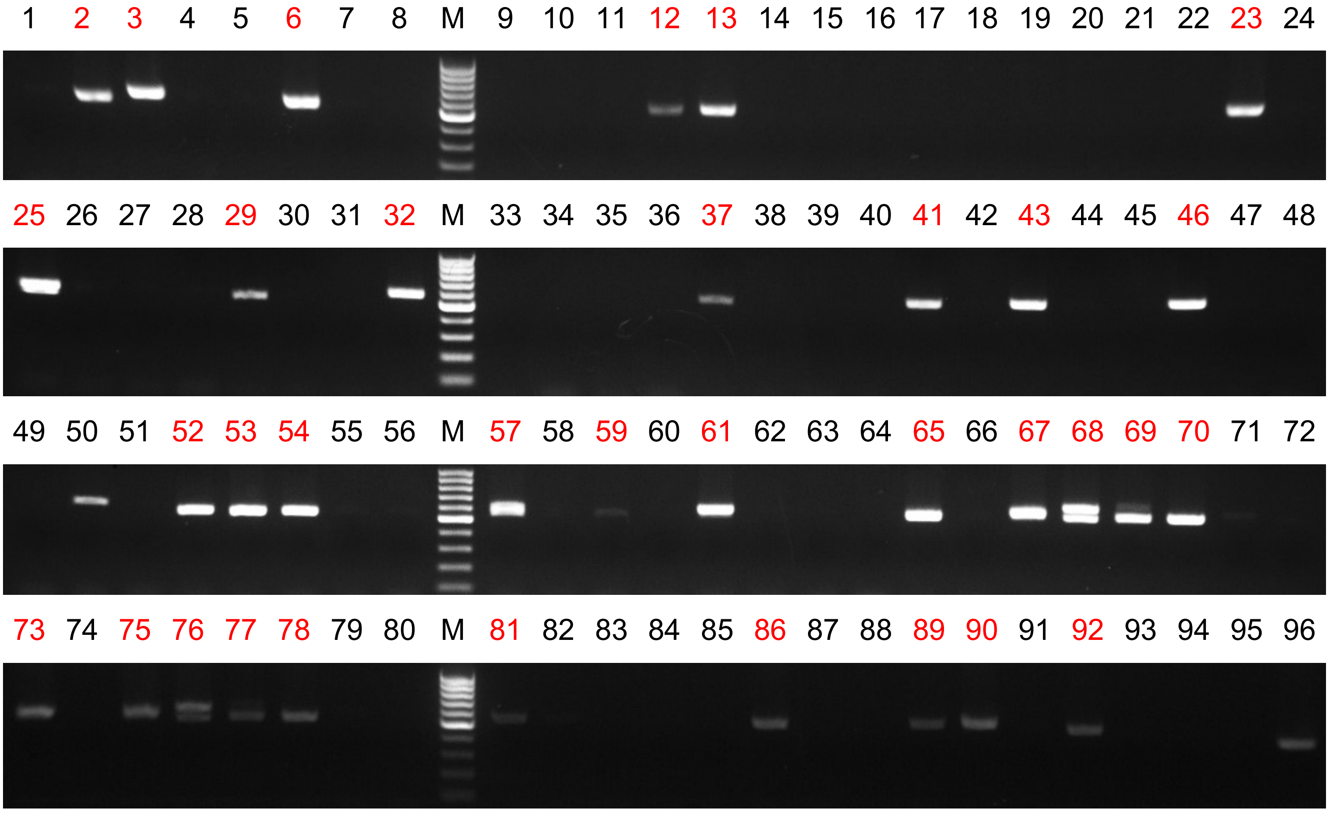

target band – 543 bp

cx55.5-sg-1

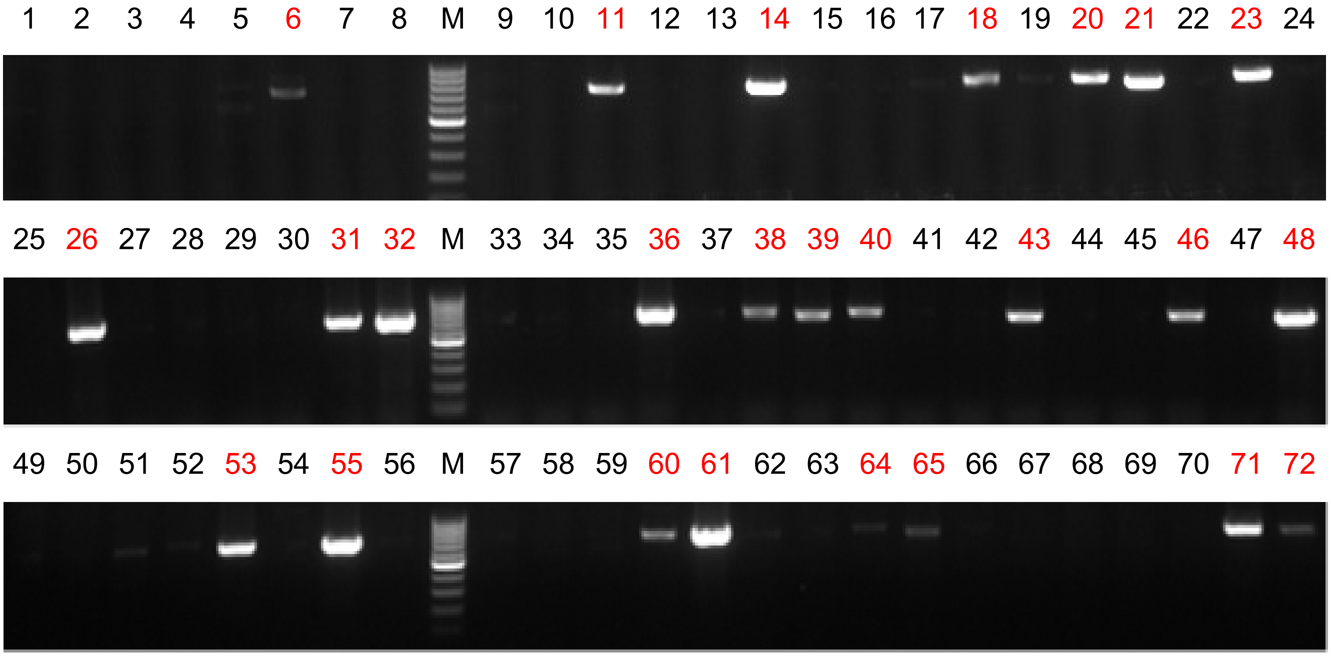

target band – 555 bp

cx55.5-sg-2

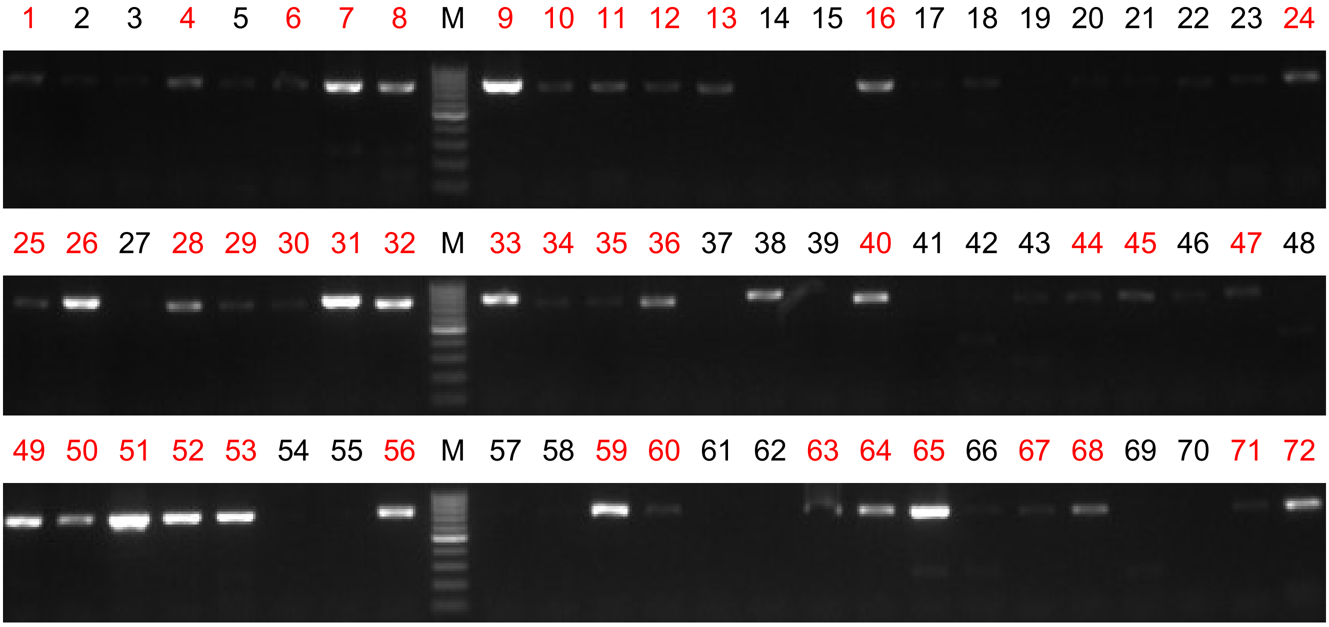

target band – 555 bp
