# Supplementary material for: Optimal tagging strategies for illuminating expression profiles of genes with different abundance in zebrafish
Source: Commun Biol. 2023 Dec 21;6:1300. doi: 10.1038/s42003-023-05686-1 (PMC10739737; doi:10.1038/s42003-023-05686-1)
Supplement: Supplementary file 5 — Supplementary Data 2 [file 42003_2023_5686_MOESM5_ESM.pdf]

***cx43*-S-25**

right arm-*lamGolden*-left arm-CDS-$GGGGS_3$-eGFP-SV40 polyA-bGH polyA

TGATACACATGAGCACAAGAAACTGCCCGTGCAGGAAGTCGTTGACCCATGCACAA
GCCTTCGACTACCCTGACGACACGCACGAACATAAAAAGCTTACACCGGGTCACGAA
TTGCAGCCATTGGCGTTGATAGATGCACGGCCGTGCAGCCGTGCCAGCAGCCGCAT
GAGCAGTCGAGCGAGGCCTGATGACCTGGACGTCGTCGACGGAGGAGGTGGTTCA
GGTGGTGGAGGATCTGGAGGTGGAGGTTCAGTGAGCAAGGGCGAGGAGCTGTTCA
CCGGGGTGGTGCCCATCCTGGTCGAGCTGGACGGCGACGTAAACGGCCACAAGTT
CAGCGTGTCCGGCGAGGGCGAGGGCGATGCCACCTACGGCAAGCTGACCCTGAAG
TTCATCTGCACCACCGGCAAGCTGCCCGTGCCCTGGCCCACCCTCGTGACCACCCT
GACCTACGGCGTGCAGTGCTTCAGCCGCTACCCCGACCACATGAAGCAGCACGACT
TCTTCAAGTCCGCCATGCCCGAAGGCTACGTCCAGGAGCGCACCATCTTCTTCAAG
GACGACGGCAACTACAAGACCCGCGCCGAGGTGAAGTTCGAGGGCGACACCCTGG
TGAACCGCATCGAGCTGAAGGGCATCGACTTCAAGGAGGACGGCAACATCCTGGG
GCACAAGCTGGAGTACAACTACAACAGCCACAACGTCTATATCATGGCCGACAAGCA
GAAGAACGGCATCAAGGTGAACTTCAAGATCCGCCACAACATCGAGGACGGCAGCG
TGCAGCTCGCCGACCACTACCAGCAGAACACCCCCATCGGCGACGGCCCCGTGCT
GCTGCCCGACAACCACTACCTGAGCACCCAGTCCGCCCTGAGCAAAGACCCCAAC
GAGAAGCGCGATCACATGGTCCTGCTGGAGTTCGTGACCGCCGCCGGGATCACTCT
CGGCATGGACGAGCTGTACAAGTAATCTAGAGATCCAGACATGATAAGATACATTGAT
GAGTTTGGACAAACCACAACTAGAATGCAGTGAAAAAAATGCTTTATTTGTGAAATTT
GTGATGCTATTGCTTTATTTGTAACCATTATAAGCTGCAATAAACAAGTTAACAACAACA
ATTGCATTCATTTTATGTTTCAGGTTCAGGGGGAGGTGTGGGAGGTTTTTTAATTCGC
GGCCGCGAATTCACTAGTGATTGCTCGAGTGGCCAGATCCTGTGCCTTCTAGTTGCC
AGCCATCTGTTGTTTGCCCCTCCCCCGTGCCTTCCTTGACCCTGGAAGGTGCCACT
CCCACTGTCCTTTCCTAATAAAATGAGGAAATTGCATCGCATTGTCTGAGTAGGTGTC
ATTCTATTCTGGGGGGTGGGGTGGGGCAGGACAGCAAGGGGGAGGATTGGGAAGA
CAATAGCAGGCATGCTGGGGATGCGGTGGGCTCTATGG

cx43-S-10, *cx43*-S-40, *cx43*-S-100 KI donors were the same as *cx43*-S-25 KI donor except for microhomologous arms

***cx43*-D-25**

***lamGolden*-left arm-CDS-$GGGGS_3$-eGFP-SV40 polyA-bGH polyA-right arm-*lampery* golden**

CCCGTGCAGGAAGTCGTTGACCCATGCACAAGCCTTCGACTACCCTGACGACACGC
ACGAACATAAAAAGCTTACACCGGGTCACGAATTGCAGCCATTGGCGTTGATAGATG
CACGGCCGTGCAGCCGTGCCAGCAGCCGCATGAGCAGTCGAGCGAGGCCTGATGA
CCTGGACGTCGTCGACGGAGGAGGTGGTTCAGGTGGTGGAGGATCTGGAGGTGGA
GGTTCAGTGAGCAAGGGCGAGGAGCTGTTCACCGGGGTGGTGCCCATCCTGGTCG
AGCTGGACGGCGACGTAAACGGCCACAAGTTCAGCGTGTCCGGCGAGGGCGAGG
GCGATGCCACCTACGGCAAGCTGACCCTGAAGTTCATCTGCACCACCGGCAAGCTG
CCCGTGCCCTGGCCCACCCTCGTGACCACCCTGACCTACGGCGTGCAGTGCTTCA
GCCGCTACCCCGACCACATGAAGCAGCACGACTTCTTCAAGTCCGCCATGCCCGAA

GGCTACGTCCAGGAGCGCACCATCTTCTTCAAGGACGACGGCAACTACAAGACCCG
CGCCGAGGTGAAGTTCGAGGGCGACACCCTGGTGAACCGCATCGAGCTGAAGGGC
ATCGACTTCAAGGAGGACGGCAACATCCTGGGGCACAAGCTGGAGTACAACTACAA
CAGCCACAACGTCTATATCATGGCCGACAAGCAGAAGAACGGCATCAAGGTGAACTT
CAAGATCCGCCACAACATCGAGGACGGCAGCGTGCAGCTCGCCGACCACTACCAG
CAGAACACCCCCATCGGCGACGGCCCCGTGCTGCTGCCCGACAACCACTACCTGA
GCACCCAGTCCGCCCTGAGCAAAGACCCCAACGAGAAGCGCGATCACATGGTCCT
GCTGGAGTTCGTGACCGCCGCCGGGATCACTCTCGGCATGGACGAGCTGTACAAGT
AATCTAGAGATCCAGACATGATAAGATACATTGATGAGTTTGGACAAACCACAACTAG
AATGCAGTGAAAAAAATGCTTTATTTGTGAAATTTGTGATGCTATTGCTTTATTTGTAAC
CATTATAAGCTGCAATAAACAAGTTAACAACAACAATTGCATTCATTTTATGTTTCAGGT
TCAGGGGGAGGTGTGGGAGGTTTTTTAATTCGCGGCCGCGAATTCACTAGTGATTGC
TCGAGTGGCCAGATCCTGTGCCTTCTAGTTGCCAGCCATCTGTTGTTTGCCCCTCCC
CCGTGCCTTCCTTGACCCTGGAAGGTGCCACTCCCACTGTCCTTTCCTAATAAAATG
AGGAAATTGCATCGCATTGTCTGAGTAGGTGTCATTCTATTCTGGGGGGTGGGGTGG
GGCAGGACAGCAAGGGGGAGGATTGGGAAGACAATAGCAGGCATGCTGGGGATGC
GGTGGGCTCTATGGTGATACACATGAGCACAAGAAACTGGTCGACCCCGTGCAGGA
AGTCGTTGACCC

cx43-D-10, *cx43*-D-40, *cx43*-D-100 KI donors were the same as *cx43*-D-25 KI donor except for microhomologous arms

***cx43*-HR**

lamGolden*-left arm-CDS-$GGGGS_3$-eGFP-right arm-*lamGolden

CCCGTGCAGGAAGTCGTTGACCCGTGCGTTGGGAAGGCTTCTTGACAAGGTGCAG
GCCTACTCCACGGCCGGAGGGAAGGTCTGGCTCTCTGTGCTCTTCATCTTCCGGAT
CCTTGTTCTGGGAACAGCAGTGGAATCGGCCTGGGGTGACGAGCAGTCAGCTTTCA
AGTGCAATACCCAGCAGCCTGGTTGCGAGAATGTCTGCTATGACAAATCGTTCCCCA
TCTCGCACGTGCGCTTCTGGGTGCTTCAGATCATCTTCGTGTCCACGCCGACGCTC
CTGTACCTGGCGCATGTCTTCTACCTGATGCGAAAGGAGGAGAAACTCAACCGTAAA
GAAGAGGAGCTGAAGGCCGTGCAGAACGACGGCGGCGACGTTGAGCTCCATCTCA
AGAAAATCGAGCTCAAGAAGTTTAAGCATGGCCTAGAGGAGCACGGCAAGGTGAAG
ATGAAGGGTAGCCTGCTGCGCACCTACATCTTCAGCATCATTTTCAAGTCCATCTGTG
AGGTGGTCTTCCTGGTCATCCAATGGTACCTCTACGGCTTCAGCCTCTCTGCCGTGT
ACACATGCGAACGCACGCCTTGCCCTCATAGGGTGGACTGTTTCCTTTCTCGGCCC
ACCGAGAAGACCATCTTCATCATCTTCATGCTAGTGGTTTCGCTCTTCTCGCTTTTGC
TCAACATCATCGAGCTCTTCTACGTGCTCTTCAAACGAATCAAGGACCGCGTCAAAA
GCCGACAAAACACACAGTTTCCCACTGGCACTTTGAGCCCCACGCCGAAGGAACTG
TCTACGACCAAATACGCGTACTACAATGGTTGCTCCTCACCAACTGCACCGCTCTCA
CCAATGTCACCTCCAGGCTACAAACTGGCCACCGGCGAAAGGACCAACTCTTGCCG
CAATTACAACAAGCAGGCTAATGAGCAGAATTGGGCCAACTACAGCACAGAACAGAA
TCGCTTGGGCCAGAATGGCAGCACCATCTCCAATTCACATGCACAAGCCTTCGACTA
CCCTGACGACACGCACGAACATAAAAAGCTTACACCGGGTCACGAATTGCAGCCATT
GGCGTTGATAGATGCACGGCCGTGCAGCCGTGCCAGCAGCCGCATGAGCAGTCGA

GCGAGGCCTGATGACCTGGACGTCGTCGACGGAGGAGGTGGTTCAGGTGGTGGAG
GATCTGGAGGTGGAGGTTCAGTGAGCAAGGGCGAGGAGCTGTTCACCGGGGTGGT
GCCCATCCTGGTCGAGCTGGACGGCGACGTAAACGGCCACAAGTTCAGCGTGTCC
GGCGAGGGCGAGGGCGATGCCACCTACGGCAAGCTGACCCTGAAGTTCATCTGCA
CCACCGGCAAGCTGCCCGTGCCCTGGCCCACCCTCGTGACCACCCTGACCTACGG
CGTGCAGTGCTTCAGCCGCTACCCCGACCACATGAAGCAGCACGACTTCTTCAAGT
CCGCCATGCCCGAAGGCTACGTCCAGGAGCGCACCATCTTCTTCAAGGACGACGGC
AACTACAAGACCCGCGCCGAGGTGAAGTTCGAGGGCGACACCCTGGTGAACCGCA
TCGAGCTGAAGGGCATCGACTTCAAGGAGGACGGCAACATCCTGGGGCACAAGCT
GGAGTACAACTACAACAGCCACAACGTCTATATCATGGCCGACAAGCAGAAGAACGG
CATCAAGGTGAACTTCAAGATCCGCCACAACATCGAGGACGGCAGCGTGCAGCTCG
CCGACCACTACCAGCAGAACACCCCCATCGGCGACGGCCCCGTGCTGCTGCCCGA
CAACCACTACCTGAGCACCCAGTCCGCCCTGAGCAAAGACCCCAACGAGAAGCGC
GATCACATGGTCCTGCTGGAGTTCGTGACCGCCGCCGGGATCACTCTCGGCATGGA
CGAGCTGTACAAGTAACCATGCCACATCCCAACGCTAGAGGACTGACACAGGCAATG
AGACATGCTCATCTCCTGTAACGAGGAGGCGAGGCTGAATCCTCGCAAGTTCCTCAC
TCTAGACTCTAGTCGATACAGAGACATAAAATTACTCACACTGCTGGAGGTACTATTCA
TTAACAATGATGTTGAAACTAGCAGAGCTAACATGCCCTAACTACCGAACAGTCAAGG
GGGCTCGTCCGGGACGAACGGCACTGAACTTGCCACGTCTCTCAACTCAGCCCAC
CAGAACAGACCTTGGCGATGTGATTGATTTTTGTTGTGCTTGTTGAAATGCCACACAA
TGATTCCACTTTAACACTTTGCACTCCTACAGTTTGTGTAGATTTGTGTTCTAACCCCC
CCTACAGTCGATCCCGGTTATAACGTCAATCGTTCGCATGATCTCAAATCCAACAGGG
TGTTCACAGTCATGAATTAATATTTATTTATTCATAAAGATTAACATGATATCCACCACTA
GATAAAGATTGACTACTACTCAAGGTCATGTCTACACAATTAATACCATCCAAGTGCAT
ATTTATTGACAATAATCCAAATTCCAGCAATACGTTCATCAGCAAATGTGTTTTTTTTTT
TTTTTGTTATTAATCACAGTGGACTGTCATTTTCCTGATAAACTTTGAATTTATGTGGTG
TTGGTTTTTGTTATTCGATGATATTTGTTGTATTTTGGGTGGTGGTATGAAACGGAAAT
GTCTTATATGTGGACTGAGCTGCTGTAACTTACACGGCTTGATTCTTTCTTTTTTCTGC
ATCTTGATTTTTACTGAATAGTATGAGAGAGAAAGGCTTTATTTTTGTTCTCTTTTTTATT
TCTTATTTTCCTTTATTTTTGGAGGGCAGACAGAATGATTTAGTTTTTTTTTTTATTAGAA
TGAATAGAAATTAACGTCTTACCTTTGGAGTGAATGACCTTTTTAACTCAATGTAATCG
TTGCTGCTTGAGAGATCCCGTGCAGGAAGTCGTTGACCC

***cx43.4*-S-25**

right arm-*lamGolden*-left arm-CDS-GGGGS$_3$-eGFP-SV40 polyA-bGH polyA

GCGCAGGCACCTGAAACTGGCTCAGCCCGTGCAGGAAGTCGTTGACCCCGTCATC
GCGAGACATTGACCGCCTTCGTCGTCATTTAAAGTTAGCCCAACAGCACCTGGACCT
CGCCTATCAGAATGGCGAGAGCAGTCCTTCACGCAGCAGCAGCCCAGAGTCCAACG
GCACTGCTGTCGAGCAGAACAGACTTAACTTTGCTCAGGAGAAGCAGGGGAGCAAA
TGTGAAAAAGGGATCCATGCTGTCGACGGAGGAGGTGGTTCAGGTGGTGGAGGATC
TGGAGGTGGAGGTTCAGTGAGCAAGGGCGAGGAGCTGTTCACCGGGGTGGTGCC
CATCCTGGTCGAGCTGGACGGCGACGTAAACGGCCACAAGTTCAGCGTGTCCGGC
GAGGGCGAGGGCGATGCCACCTACGGCAAGCTGACCCTGAAGTTCATCTGCACCA
CCGGCAAGCTGCCCGTGCCCTGGCCCACCCTCGTGACCACCCTGACCTACGGCGT

GCAGTGCTTCAGCCGCTACCCCGACCACATGAAGCAGCACGACTTCTTCAAGTCCG
CCATGCCCGAAGGCTACGTCCAGGAGCGCACCATCTTCTTCAAGGACGACGGCAAC
TACAAGACCCGCGCCGAGGTGAAGTTCGAGGGCGACACCCTGGTGAACCGCATCG
AGCTGAAGGGCATCGACTTCAAGGAGGACGGCAACATCCTGGGGCACAAGCTGGA
GTACAACTACAACAGCCACAACGTCTATATCATGGCCGACAAGCAGAAGAACGGCAT
CAAGGTGAACTTCAAGATCCGCCACAACATCGAGGACGGCAGCGTGCAGCTCGCC
GACCACTACCAGCAGAACACCCCCATCGGCGACGGCCCCGTGCTGCTGCCCGACA
ACCACTACCTGAGCACCCAGTCCGCCCTGAGCAAAGACCCCAACGAGAAGCGCGAT
CACATGGTCCTGCTGGAGTTCGTGACCGCCGCCGGGATCACTCTCGGCATGGACGA
GCTGTACAAGTAATCTAGAGATCCAGACATGATAAGATACATTGATGAGTTTGGACAAA
CCACAACTAGAATGCAGTGAAAAAAATGCTTTATTTGTGAAATTTGTGATGCTATTGCT
TTATTTGTAACCATTATAAGCTGCAATAAACAAGTTAACAACAACAATTGCATTCATTTT
ATGTTTCAGGTTCAGGGGGAGGTGTGGGAGGTTTTTTAATTCGCGGCCGCGAATTCA
CTAGTGATTGCTCGAGTGGCCAGATCCTGTGCCTTCTAGTTGCCAGCCATCTGTTGT
TTGCCCCTCCCCCGTGCCTTCCTTGACCCTGGAAGGTGCCACTCCCACTGTCCTTT
CCTAATAAAATGAGGAAATTGCATCGCATTGTCTGAGTAGGTGTCATTCTATTCTGGG
GGGTGGGGTGGGGCAGGACAGCAAGGGGGAGGATTGGGAAGACAATAGCAGGCAT
GCTGGGGATGCGGTGGGCTCTATGG

cx43.4-S-10, *cx43.4*-S-40, *cx43.4*-S-100 KI donors were the same as *cx43.4*-S-25 KI donor except for microhomologous arms

***cx43.4*-D-25**

lamGolden*-left arm-CDS-GGGGS$_3$-eGFP-SV40 polyA-bGH polyA-right arm-*lampery golden

CCCGTGCAGGAAGTCGTTGACCCCGTCATCGCGAGACATTGACCGCCTTCGTCGTC
ATTTAAAGTTAGCCCAACAGCACCTGGACCTCGCCTATCAGAATGGCGAGAGCAGTC
CTTCACGCAGCAGCAGCCCAGAGTCCAACGGCACTGCTGTCGAGCAGAACAGACTT
AACTTTGCTCAGGAGAAGCAGGGGAGCAAATGTGAAAAAGGATCCATGCTGTCGA
CGGAGGAGGTGGTTCAGGTGGTGGAGGATCTGGAGGTGGAGGTTCAGTGAGCAAG
GGCGAGGAGCTGTTCACCGGGGTGGTGCCCATCCTGGTCGAGCTGGACGGCGAC
GTAAACGGCCACAAGTTCAGCGTGTCCGGCGAGGGCGAGGGCGATGCCACCTACG
GCAAGCTGACCCTGAAGTTCATCTGCACCACCGGCAAGCTGCCCGTGCCCTGGCC
CACCCTCGTGACCACCCTGACCTACGGCGTGCAGTGCTTCAGCCGCTACCCCGACC
ACATGAAGCAGCACGACTTCTTCAAGTCCGCCATGCCCGAAGGCTACGTCCAGGAG
CGCACCATCTTCTTCAAGGACGACGGCAACTACAAGACCCGCGCCGAGGTGAAGTT
CGAGGGCGACACCCTGGTGAACCGCATCGAGCTGAAGGGCATCGACTTCAAGGAG
GACGGCAACATCCTGGGGCACAAGCTGGAGTACAACTACAACAGCCACAACGTCTA
TATCATGGCCGACAAGCAGAAGAACGGCATCAAGGTGAACTTCAAGATCCGCCACAA
CATCGAGGACGGCAGCGTGCAGCTCGCCGACCACTACCAGCAGAACACCCCCATC
GGCGACGGCCCCGTGCTGCTGCCCGACAACCACTACCTGAGCACCCAGTCCGCCC
TGAGCAAAGACCCCAACGAGAAGCGCGATCACATGGTCCTGCTGGAGTTCGTGACC
GCCGCCGGGATCACTCTCGGCATGGACGAGCTGTACAAGTAATCTAGAGATCCAGA
CATGATAAGATACATTGATGAGTTTGGACAAACCACAACTAGAATGCAGTGAAAAAAAT

GCTTTATTTGTGAAATTTGTGATGCTATTGCTTTATTTGTAACCATTATAAGCTGCAATAA
ACAAGTTAACAACAACAATTGCATTCATTTTATGTTTCAGGTTCAGGGGGAGGTGTGG
GAGGTTTTTTAATTCGCGGCCGCGAATTCACTAGTGATTGCTCGAGTGGCCAGATCC
TGTGCCTTCTAGTTGCCAGCCATCTGTTGTTTGCCCCTCCCCCGTGCCTTCCTTGAC
CCTGGAAGGTGCCACTCCCACTGTCCTTTCCTAATAAAATGAGGAAATTGCATCGCAT
TGTCTGAGTAGGTGTCATTCTATTCTGGGGGGTGGGGTGGGGCAGGACAGCAAGGG
GGAGGATTGGGAAGACAATAGCAGGCATGCTGGGGATGCGGTGGGCTCTATGGGC
GCAGGCACCTGAAACTGGCTCAGGTCGACCCCGTGCAGGAAGTCGTTGACCC

cx43.4-D-10, *cx43.4*-D-40, *cx43.4*-D-100 KI donors were the same as *cx43.4*-D-25 KI donor except for microhomologous arms

***cx43.4*-HR**

lamGolden*-left arm-CDS-GGGGS$_3$-eGFP-right arm-*lamGolden

CCCGTGCAGGAAGTCGTTGACCCAGGACTGAGACGGTGGTACTTCTTGAAGCACCA
TGAGCTGGAGTTTTCTTACGCGGTTGTTGGATGAAATCTCCAACCACTCCACCTTCG
TGGGCAAGATATGGCTCACGTTATTCATCATCTTCCGCATTGTTTTGACTGTTGTGGG
GGGAGAATCGATATACTACGATGAACAGAGCAAATTTGTGTGTAATACCCAGCAACCT
GGTTGTGAGAACGTTTGCTACGATGCATTTGCACCACTCTCTCATGTCCGGTTCTGG
GTTTTCCAGATCATTTTGATCACAACCCCCACTATCATGTACTTGGGATTTGCTATGCA
CAAGATCGCTCGGTCAAATGATGTGGAGTACAGGCCAGTCAACAGGAAACGCATGC
CAATGATCAACCGCGGAGCCAACCGGGATTATGAGGAGGCCGAAGACAACGGTGAG
GAAGATCCTATGATTATGGAAGAGATCGTGCCTGAGAAAGAAAAGGCTCCAGAGAAG
TCTGCTGTTAAACATGACGGCCGGCGGAGAATAAAGCGAGATGGGCTCATGAAGGT
GTACATCCTGCAGCTTCTGTCGAGGATTATTTTCGAGGTGGGCTTTCTCTTTGGCCA
GTATATCCTGTATGGTTTCGAGGTCGCCCCGTCATACGTGTGCACTCGCAGTCCCTG
CCCGCACACCGTAGACTGCTTTGTGTCACGTCCGACAGAGAAAACCATCTTTCTGCT
GATTATGTATGCCGTGAGCTGTCTCTGCTTGTCTCTTACGGTGCTGGAGATACTTCAT
TTGGGCCTCAGCGGAATTCGTGATGCTTTTCGACGACGTGCACGCCATCAAAGTGTT
CAGCGCCCACGTGCCCCCATATGCAGACAGGTGCCCACTGCCCCGCCAGGGTACC
ACACTGCCCTGAAAAAAGACAAGCTGTCTTTGGGAATGAAACCGGAGTATAACTTGG
ACTCCGGTCGGGAGTCTTTTGGTGACGAGTCGTCATCGCGAGACATTGACCGCCTT
CGTCGTCATTTAAAGTTAGCCCAACAGCACCTGGACCTCGCCTATCAGAATGGCGAG
AGCAGTCCTTCACGCAGCAGCAGCCCAGAGTCCAACGGCACTGCTGTCGAGCAGA
ACAGACTTAACTTTGCTCAGGAGAAGCAGGGGAGCAAATGTGAAAAAGGGATCCATG
CTGTCGACGGAGGAGGTGGTTCAGGTGGTGGAGGATCTGGAGGTGGAGGTTCAGT
GAGCAAGGGCGAGGAGCTGTTCACCGGGGTGGTGCCCATCCTGGTCGAGCTGGAC
GGCGACGTAAACGGCCACAAGTTCAGCGTGTCCGGCGAGGGCGAGGGCGATGCCA
CCTACGGCAAGCTGACCCTGAAGTTCATCTGCACCACCGGCAAGCTGCCCGTGCCC
TGGCCCACCCTCGTGACCACCCTGACCTACGGCGTGCAGTGCTTCAGCCGCTACCC
CGACCACATGAAGCAGCACGACTTCTTCAAGTCCGCCATGCCCGAAGGCTACGTCC
AGGAGCGCACCATCTTCTTCAAGGACGACGGCAACTACAAGACCCGCGCCGAGGT
GAAGTTCGAGGGCGACACCCTGGTGAACCGCATCGAGCTGAAGGGCATCGACTTCA
AGGAGGACGGCAACATCCTGGGGCACAAGCTGGAGTACAACTACAACAGCCACAAC

GTCTATATCATGGCCGACAAGCAGAAGAACGGCATCAAGGTGAACTTCAAGATCCGC
CACAACATCGAGGACGGCAGCGTGCAGCTCGCCGACCACTACCAGCAGAACACCC
CCATCGGCGACGGCCCCGTGCTGCTGCCCGACAACCACTACCTGAGCACCCAGTC
CGCCCTGAGCAAAGACCCCAACGAGAAGCGCGATCACATGGTCCTGCTGGAGTTCG
TGACCGCCGCCGGGATCACTCTCGGCATGGACGAGCTGTACAAGTAATTGAGACTC
ATAGACTATCATTCGTCTGCTTTTCTTTGCAACGGAGCGCCTCACCTGGGATTAGCAA
TGCTCATCTTGCAAAGTGGAAAGAGTCCAGTTTGTGTGTGTGTGTGTTGTGTGTTTT
GTGTTTGGCTGCTGAACATTGAGCGAAGCATCAAGAGACTGAGTGTGGAACCTGTG
TCCTACGCACACTGGTGAATCTGTGAGAATGTGTCATGCAGTATTATGGCAAGTGAAC
GCCATGTAGAAAATCTCAGAAGCAGCATTTCACCAGCCAGTTATACTTGACATCGGCC
AGTCTGCCTGATACTCCAAAGTGCCCAAAAGGTAGTTAGGAAAATGTTTGTTATACTC
CTCTCTTACTAGGCGACTGTGCAATGTCTCGTTTTTTTATCATCTCTTTTTATTACGTAG
GGGGGAAAAAAATCAGTGGTTCTTCCATTTGTTTACCACGCTTCTAATGAAGTGCCAA
GGGTTGCATCTCAAAAATGCCTTAAACTGGAGTTTACATATTATCTTTGCCATTCCAAA
ATGAGATGGACGAGCCAGTGTAAAACCACATCAGAAATGTAGTTTAGTGTAGTTAGAG
CATTTCTGTAGTTCTGTGTTTGGAGTAGTGTGTTTGTAACGGAAACCTGGAAAATGCT
TGAATACTTGTGAATCAATTATTGATCTGCAAAGAAGCTTTTGATTATTTTATTTGCATAT
TGTGTACTTGGTTGTATATGAGAATTTTTGCTACTACCCCTATTGGAACTGAAAGTCAT
GGAGGCGAATGTTTTATATGGTACCAATGCAACTTTGTTTTTCTTTTTGTCTTGAAACT
GTTTAAAGTATAAGAGTTTAATGTCTGTCAACACCCAGGTTTTCCAACTTTTGATACTT
TTTTCAACAATACTTTTTCAACTTTTGATACTTTTTCAACTTTGATACTTTTTTTTTTTTAT
ATCTTCCAAAACATTTTGTACGATTTTTTGTAAGGTATTTTGTACACTGTCCACCCGTG
CAGGAAGTCGTTGACCC

***cx43.4*-NHEJ**

***lamGolden*-intron and CDS-GGGGS$_3$-eGFP-SV40 polyA-bGH polyA**

CCCGTGCAGGAAGTCGTTGACCCTTATGGACACCAATAATCCAATTTTAATATGATTGA
CTACTCTGATTAGGAGTCTACCATGTAAACAGCTGTTTTTGATTTATTAAATCCGATTAA
AGTTTTATAATCGAACTAAACAGAAATCAGATTAAGACATGTGGAGTATGCTGATTTTAT
TGCCATTGAAGTGCATTTTAGTTTTATTAAAGTGCAGTACAGACATGTAAACATTGCAA
ACAAACTATTATCATTGTGTAGGATTTTCTCAGCATTTTACAGGATACAGGATAGTCAAT
ACACACATTTCTGTTTGACACTATTTTCTGCACCTACCAAGTCCGTAAAGGACCACAG
ACACCTGCATCACAAAATGCGAAGGTTTTCTTTCCCCATACGGTGTGCGGTATCAAAT
TCCATGAAAACAACACTAGCAGTTCAGACTGACTTCCAATATCTCGTTTATCACGGGG
GCATGCATGAAATGTTCCTGAATGAAAGTAAAAGTGCCAAATTGCAGTTAAAGTCCAC
AAATTAAAAATGAAACACCCAAAAGTAAATGAAACTCAAAAGAAAATGTTGATAGCGTG
GTGACAATGGTGTTAACCAATTATGTGCTATAACATGTAAAACGAGATCATTCAAAAAG
CAACTCATGTAAACGCCTTAATCATATTACTGTCTTTTTCAGATTAAGGCATATAATTAG
ATTACTGATGTCCATGTAAACGTAGTCACTCTGTCACTAAGGTTCAGCTAACATTAAGC
TGGACTATGATGATAACAGGTCTGACCAGGGTTACTCATTTTTTAGCTTGCCAGCAAT
TTGCCACTTCTAGTAGAAGTATAAAACCTATTTCATCCAATTGCTTATGTTGTCTAATTG
CTTGTATGTCTCCTAATTGGCACCTATACTCAATGAGCGCAATCATTGTATGACCAAAT
TAAGGTGTTTGCTTTATTGAATTGTACAAAAATACACACATACCTATCAAATTAGTAGTT
TTTAAGTTTTTTAATCAAGTAAAGCTGCGGTCACACTAGAGTTTGATAATGCGATATTC

TGTCGTGCGGCTCTGCGAAAAGGGGCGGGAATGAACAAGATTATCAGGCATTAAAAA
AAGCAAGCAATTGCTCCATGTTTTAAAGTTCTGTCCATTGAAGTCATGTTTTGATACTA
GGTTGGTCTCACGCAGTCAAGTGTTGCGATTTCGCAGATCAGAGTTCACCAAGCTTG
AACTTTGCATCGCAGCAACCTGCAAAACTTGACGCATGACCCTGCGTTTTCGGTCTG
ACGCATTTGCGTGCGTATGAATGGAAGTCTTATGTGGAGAAAAGCCCAGTGTGACCG
CAGCTTGAGAGAGTCTTATTTGCCACTTGGGTTTCACCAAAATCCTAAGTTGAAAACC
TGTCAATATCCGCTGGAAACTAATGTATCTGAGCTAACCACTTTTGATTGAATCAATGA
AGCGCCAAGTAGAGATGGCCTAATATTCAAAAGTTTTGGATAAGTTCATCCAAAAGTT
CAACTAATCAAATACGACAGCAGATAATTGCATTTTTTTGTATCTTTTTTTTTTAACCAA
AAATAAATCGTAGTAAAAAAAAAATGTTTCCATTCTCCATGTCTTTGTGTTCACTCTAAG
GATAGTTCACTCAAATAAAAATGGTCATTTACTCAATCTTCATTTGATCGACCAATTTGA
GTTTCTTTCTCCAAAGAAGATATTTTAGTAATTAAACCAAGTCAATGGGTCCCAGCTAT
CAGCATACTCAACAGCAGAAAGAAACTCATAAAGGCTTAACTACATGAGGGAGAATGT
ATAATTAGGTTGTTTTTAATTTGTGAGAAGTTAACATATTTGTTGCTTGATAATGTAAATA
AATGATTTCCATGTTTTAATATGATGTTGTCTTTTCTCTTTCTCAGGGATCCATGCTGTC
GACGGAGGAGGTGGTTCAGGTGGTGGAGGATCTGGAGGTGGAGGTTCAGTGAGCA
AGGGCGAGGAGCTGTTCACCGGGGTGGTGCCCATCCTGGTCGAGCTGGACGGCGA
CGTAAACGGCCACAAGTTCAGCGTGTCCGGCGAGGGCGAGGGCGATGCCACCTAC
GGCAAGCTGACCCTGAAGTTCATCTGCACCACCGGCAAGCTGCCCGTGCCCTGGC
CCACCCTCGTGACCACCCTGACCTACGGCGTGCAGTGCTTCAGCCGCTACCCCGAC
CACATGAAGCAGCACGACTTCTTCAAGTCCGCCATGCCCGAAGGCTACGTCCAGGA
GCGCACCATCTTCTTCAAGGACGACGGCAACTACAAGACCCGCGCCGAGGTGAAGT
TCGAGGGCGACACCCTGGTGAACCGCATCGAGCTGAAGGGCATCGACTTCAAGGA
GGACGGCAACATCCTGGGGCACAAGCTGGAGTACAACTACAACAGCCACAACGTCT
ATATCATGGCCGACAAGCAGAAGAACGGCATCAAGGTGAACTTCAAGATCCGCCACA
ACATCGAGGACGGCAGCGTGCAGCTCGCCGACCACTACCAGCAGAACACCCCCAT
CGGCGACGGCCCCGTGCTGCTGCCCGACAACCACTACCTGAGCACCCAGTCCGCC
CTGAGCAAAGACCCCAACGAGAAGCGCGATCACATGGTCCTGCTGGAGTTCGTGAC
CGCCGCCGGGATCACTCTCGGCATGGACGAGCTGTACAAGTAATCTAGAGATCCAG
ACATGATAAGATACATTGATGAGTTTGGACAAACCACAACTAGAATGCAGTGAAAAAA
ATGCTTTATTTGTGAAATTTGTGATGCTATTGCTTTATTTGTAACCATTATAAGCTGCAAT
AAACAAGTTAACAACAACAATTGCATTCATTTTATGTTTCAGGTTCAGGGGGAGGTGT
GGGAGGTTTTTTAATTCGCGGCCGCGAATTCACTAGTGATTGCTCGAGTGGCCAGAT
CCTGTGCCTTCTAGTTGCCAGCCATCTGTTGTTTGCCCCTCCCCCGTGCCTTCCTTG
ACCCTGGAAGGTGCCACTCCCACTGTCCTTTCCTAATAAAATGAGGAAATTGCATCG
CATTGTCTGAGTAGGTGTCATTCTATTCTGGGGGGTGGGGTGGGGCAGGACAGCAA
GGGGGAGGATTGGGAAGACAATAGCAGGCATGCTGGGGATGCGGTGGGCTCTATG
G

***cx30.3*, *cx34.4*, *cx35*, *cx44.1*, *cx47.1*, *cx48.5*, *cx52.6*, *cx55.5* KI donors were designed based on the S-25 KI strategy**

***cx30.3*-sg-1**

right arm-*lamGolden*-left arm-CDS-$GGGGS_3$-eGFP-SV40 polyA-bGH polyA

AACTGGCCTATCTGATTGTCAAAGCCCCGTGCAGGAAGTCGTTGACCCATTTGCATT

GTGCTCAATGTGGCTGAGTTAGCATACTTAATCGTAAAGGCGTTGCTCAGGTGCTCA
GCCAGAGCCAAAGGGAGGTGCTCATTTGTACACCAAGAGAAAATGTCCACAGAAAA
GGCGCACCTACAGAATGAAAAAAACGCAAGGTTGCTGTCATCGGCTTCGGACTCATC
GAGCAATAAGACTGTTGTCGACGGAGGAGGTGGTTCAGGTGGTGGAGGATCTGGA
GGTGGAGGTTCAGTGAGCAAGGGCGAGGAGCTGTTCACCGGGGTGGTGCCCATCC
TGGTCGAGCTGGACGGCGACGTAAACGGCCACAAGTTCAGCGTGTCCGGCGAGGG
CGAGGGCGATGCCACCTACGGCAAGCTGACCCTGAAGTTCATCTGCACCACCGGCA
AGCTGCCCGTGCCCTGGCCCACCCTCGTGACCACCCTGACCTACGGCGTGCAGTG
CTTCAGCCGCTACCCCGACCACATGAAGCAGCACGACTTCTTCAAGTCCGCCATGC
CCGAAGGCTACGTCCAGGAGCGCACCATCTTCTTCAAGGACGACGGCAACTACAAG
ACCCGCGCCGAGGTGAAGTTCGAGGGCGACACCCTGGTGAACCGCATCGAGCTGA
AGGGCATCGACTTCAAGGAGGACGGCAACATCCTGGGGCACAAGCTGGAGTACAAC
TACAACAGCCACAACGTCTATATCATGGCCGACAAGCAGAAGAACGGCATCAAGGTG
AACTTCAAGATCCGCCACAACATCGAGGACGGCAGCGTGCAGCTCGCCGACCACTA
CCAGCAGAACACCCCCATCGGCGACGGCCCCGTGCTGCTGCCCGACAACCACTAC
CTGAGCACCCAGTCCGCCCTGAGCAAAGACCCCAACGAGAAGCGCGATCACATGGT
CCTGCTGGAGTTCGTGACCGCCGCCGGGATCACTCTCGGCATGGACGAGCTGTACA
AGTAATCTAGAGATCCAGACATGATAAGATACATTGATGAGTTTGGACAAACCACAACT
AGAATGCAGTGAAAAAAATGCTTTATTTGTGAAATTTGTGATGCTATTGCTTTATTTGTA
ACCATTATAAGCTGCAATAAACAAGTTAACAACAACAATTGCATTCATTTTATGTTTCAG
GTTCAGGGGGAGGTGTGGGAGGTTTTTTAATTCGCGGCCGCGAATTCACTAGTGATT
GCTCGAGTGGCCAGATCCTGTGCCTTCTAGTTGCCAGCCATCTGTTGTTTGCCCCTC
CCCCGTGCCTTCCTTGACCCTGGAAGGTGCCACTCCCACTGTCCTTTCCTAATAAAA
TGAGGAAATTGCATCGCATTGTCTGAGTAGGTGTCATTCTATTCTGGGGGGTGGGGT
GGGGCAGGACAGCAAGGGGGAGGATTGGGAAGACAATAGCAGGCATGCTGGGGAT
GCGGTGGGCTCTATGG

***cx30.3*, *cx34.4*, *cx35*, *cx47.1*, *cx52.6* KI donors were the same as cx30.3-sg-1 KI donor except for microhomologous arms and complementary CDS**

***cx30.3*-sg-2**

right arm-*lamGolden*-left arm-CDS

CTCAGGTGCTCAGCCAGAGCCAAAGCCCGTGCAGGAAGTCGTTGACCCGGCCTATC
TGATTGTCAAAGCATTGTTGCGTTGTAGCGCACGCGCTAAGGGGAGGTGCTCATTTG
TACACCAAGAGAAAATGTCCACAGAAAAGGCGCACCTACAGAATGAAAAAAACGCAA
GGTTGCTGTCATCGGCTTCGGACTCATCGAGCAATAAGACTGTT

***cx34.4*-sg-1**

right arm-*lamGolden*-left arm-CDS

GTGTGGTCATGTCAGCCAAAAAGAACCCGTGCAGGAAGTCGTTGACCCGAAGACCA
GCCGGCACCAGCATACAGCGTAGTTATGAGCGCTAAGAAAAAGACCAGCTTGGAAAA
TACTTTCAATCCGAGTTGGACCTTGAGAGATGACAAATGTTCAACTTCACATTCACTT
GGAGGTGAATGCGAACGTGAC

***cx34.4*-sg-2**

right arm-*lamGolden*-left arm-CDS
CCATGGCTAATATGACAAACTTGAACCCGTGCAGGAAGTCGTTGACCCTCACGACAT
GTGAACAGAGAGAGATCTATGGCCAACATGACGAATCTCAACGCTCATTTAGAGTCA
AACAACAAAAAACTGGCAAGCGAAGACCAGCCGGCACCAGCATACAGTGTGGTCAT
GTCAGCCAAAAAGAAAACCAGCTTGGAAAATACTTTCAATCCGAGTTGGACCTTGAG
AGATGACAAATGTTCAACTTCACATTCACTTGGAGGTGAATGCGAACGTGAC

***cx35*-sg-1**
right arm-*lamGolden*-left arm-CDS
TTTCGGAAGAACCCAGTCCAGTGACCCCGTGCAGGAAGTCGTTGACCCACTTACCG
CGGATGAGCATGCCGAACTTTGGGCGGACTCAATCTTCAGATAGCGCATATGTG

***cx35*-sg-2**
right arm-*lamGolden*-left arm-CDS
CCAGTGACTCTGCCTACGTTTAATGCCCGTGCAGGAAGTCGTTGACCCATGCCGAAT
TTCGGAAGAACCCAGTCTTCAGATAGCGCATATGTG

***cx47.1*-sg-1**
right arm-*lamGolden*-left arm-CDS
CTGGTGGCACAACGACAGAGCAGAACCCGTGCAGGAAGTCGTTGACCCATTTCAAG
AGCCAGCAGCCCCGTTTCGGGCGGTACGACAACGGAGCAAAATCGCATCAACATGG
CTCAGGAGAAGCAGGGCGCTCGGCCCAAAGCAAGCACCGAGAGAGCTGGGACACT
AGTAAAAAATGGAAAAACTTCGGTGTGGATT

***cx47.1*-sg-2**
right arm-*lamGolden*-left arm-CDS
TTCTGGTGGCACAACGACAGAGCAGCCCGTGCAGGAAGTCGTTGACCCATATTTCA
AGAGCCAGCAGCCCCGTAAGCGGGGGAACCACTACCGAGCAAAACCGCATCAACAT
GGCTCAGGAGAAGCAGGGCGCTCGGCCCAAAGCAAGCACCGAGAGAGCTGGGAC
ACTAGTAAAAAATGGAAAAACTTCGGTGTGGATT

***cx52.6*-sg-1**
right arm-*lamGolden*-left arm-CDS
ATGACACCACCCCCTGCAGCAGGACCCCGTGCAGGAAGTCGTTGACCCTATCACTC
AAGGGGAGAGTCCCGCTATGACCCCGCCTCCAGCTGCCGGGCGTAGAATGTCAATG
AGCATGATCCTGGAACTTTCTTCAATCATGAAAAAG

***cx52.6*-sg-2**
right arm-*lamGolden*-left arm-CDS
TGAGAGTGCAGCCTCCAAGAGTGGACCCGTGCAGGAAGTCGTTGACCCCAAGGGG
ACTTTCAGAGAGCCCGTCGGAATCCGCCGCTAGTAAATCCGGCTCAGACACAGAGG
CCAACCGTATCACTCAAGGGGAGAGTCCCGCTATGACACCACCCCCTGCAGCAGGA
CGTAGAATGTCAATGAGCATGATCCTGGAACTTTCTTCAATCATGAAAAAG

***cx44.1*-sg-1**

right arm-*lamGolden*-left arm-CDS-GGGGS$_3$-mCherry-SV40 polyA-bGH polyA

GCATCGTCAGGAATTCTGCGCAGGGCCCGTGCAGGAAGTCGTTGACCCCCAGACG
ACCGTGATTGGACCGAGTGCCAGTAGCGGGATCTTAAGACGTGATGAAGATGAGGA
CGAGTTGGCTGTGGAGGCAGACATGGAGGCCAGCGAGACGATAGAAGATACACGAC
CTCTCAGCAGCCTGAGCAAGGCCAGCAGTCGCGCAAGGTCAGATGACTTGACGGTA
GTCGACGGAGGAGGTGGTTCAGGTGGTGGAGGATCTGGAGGTGGAGGTTCAGTGA
GCAAGGGCGAGGAGGATAACATGGCCATCATCAAGGAGTTCATGCGCTTCAAGGTG
CACATGGAGGGCTCCGTGAACGGCCACGAGTTCGAGATCGAGGGCGAGGGCGAG
GGCCGCCCCTACGAGGGCACCCAGACCGCCAAGCTGAAGGTGACCAAGGGTGGC
CCCCTGCCCTTCGCCTGGGACATCCTGTCCCCTCAGTTCATGTACGGCTCCAAGGC
CTACGTGAAGCACCCCGCCGACATCCCCGACTACTTGAAGCTGTCCTTCCCCGAGG
GCTTCAAGTGGGAGCGCGTGATGAACTTCGAGGACGGCGGCGTGGTGACCGTGAC
CCAGGACTCCTCCCTCCAGGACGGCGAGTTCATCTACAAGGTGAAGCTGCGCGGCA
CCAACTTCCCCTCCGACGGCCCCGTAATGCAGAAGAAGACCATGGGCTGGGAGGC
CTCCTCCGAGCGGATGTACCCCGAGGACGGCGCCCTGAAGGGCGAGATCAAGCAG
AGGCTGAAGCTGAAGGACGGCGGCCACTACGACGCTGAGGTCAAGACCACCTACA
AGGCCAAGAAGCCCGTGCAGCTGCCCGGCGCCTACAACGTCAACATCAAGTTGGAC
ATCACCTCCCACAACGAGGACTACACCATCGTGGAACAGTACGAACGCGCCGAGGG
CCGCCACTCCACCGGCGGCATGGACGAGCTGTACAAGTAATCTAGAGATCCAGACAT
GATAAGATACATTGATGAGTTTGGACAAACCACAACTAGAATGCAGTGAAAAAAATGC
TTTATTTGTGAAATTTGTGATGCTATTGCTTTATTTGTAACCATTATAAGCTGCAATAAAC
AAGTTAACAACAACAATTGCATTCATTTTATGTTTCAGGTTCAGGGGGAGGTGTGGGA
GGTTTTTTAATTCGCGGCCGCGAATTCACTAGTGATTGCTCGAGTGGCCAGATCCTG
TGCCTTCTAGTTGCCAGCCATCTGTTGTTTGCCCCTCCCCCGTGCCTTCCTTGACCC
TGGAAGGTGCCACTCCCACTGTCCTTTCCTAATAAAATGAGGAAATTGCATCGCATTG
TCTGAGTAGGTGTCATTCTATTCTGGGGGGTGGGGTGGGGCAGGACAGCAAGGGG
GAGGATTGGGAAGACAATAGCAGGCATGCTGGGGATGCGGTGGGCTCTATGG

***cx44.1*, *cx48.5*, *cx55.5* KI donors were the same as *cx44.1*-sg-1 KI donor except for microhomologous arms and complementary CDS**

***cx44.1*-sg-2**

right arm-*lamGolden*-left arm-CDS

CTGTGGAGGCAGACATGGAGGCCAGCCCGTGCAGGAAGTCGTTGACCCAGGGATG
AAGATGAGGACGAGTTGGCCGTTGAAGCCGATATGGAAGCTTCTGAGACGATAGAA
GATACACGACCTCTCAGCAGCCTGAGCAAGGCCAGCAGTCGCGCAAGGTCAGATGA
CTTGACGGTA

***cx48.5*-sg-1**

right arm-*lamGolden*-left arm-CDS

CGGAGGAGGGGCACGTCACCACCACCCCGTGCAGGAAGTCGTTGACCCGCTGGTG
GTGGTTTAAGCACTGGGCCCGAAGAAGGTCACGTGACGACGACCGTGGAGATGCA
CGAGCCGCCCGTCATTTTCACTGACGCTCGACGACTGAGCAGGGCTAGTAAAGCCA
GCAGTGTGAGAGCGAGGCCCAATGATCTGGCGGTG

***cx48.5*-sg-2**

right arm-*lamGolden*-left arm-CDS

CGGTGGAGATGCACGAGCCGCCCGTCCCGTGCAGGAAGTCGTTGACCCCCGGAGG
AGGGGCACGTCACCACCACCGTAGAAATGCACGAACCTCCTGTAATTTTCACTGACG
CTCGACGACTGAGCAGGGCTAGTAAAGCCAGCAGTGTGAGAGCGAGGCCCAATGAT
CTGGCGGTG

***cx55.5*-sg-1**

right arm-*lamGolden*-left arm-CDS

TGGCCAGCAGTTCCAGCAGCAGACGCCCGTGCAGGAAGTCGTTGACCCGCGTCAC
CAAGCCATCGTGCCTCATTAGCATCATCCAGTTCGTCTCGTAGGGCAGCTCCCACAG
ACTTACAAATT

***cx55.5*-sg-2**

right arm-*lamGolden*-left arm-CDS

TGAGCAGGCCCACCTCCCCTGATTCCCCGTGCAGGAAGTCGTTGACCCACCTCATC
CAGGTCTGATACCAAGCTATCTCGACCAACAAGTCCCGACAGTGTCGAAGAATCGAG
CTCTGAGTCACGGCATAGTCCACGAGCGTCACCAAGCCATCGTGCCTCATTGGCCA
GCAGTTCCAGCAGCAGACGAGCAGCTCCCACAGACTTACAAATT

***cx43.4*-V**

right arm-*lamGolden*-left arm-CDS-P2A-Gal4-VP64-SV40 polyA-bGH polyA-3×SV40 polyA-5×UAS-E1b-eGFP-SV40 polyA-bGH polyA-2×insulator

GCGCAGGCACCTGAAACTGGCTCAGCCCGTGCAGGAAGTCGTTGACCCCGTCATC
GCGAGACATTGACCGCCTTCGTCGTCATTTAAAGTTAGCCCAACAGCACCTGGACCT
CGCCTATCAGAATGGCGAGAGCAGTCCTTCACGCAGCAGCAGCCCAGAGTCCAACG
GCACTGCTGTCGAGCAGAACAGACTTAACTTTGCTCAGGAGAAGCAGGGGAGCAAA
TGTGAAAAAGGGATCCATGCTGGTACCGCTACTAACTTCAGCCTGCTGAAGCAGGCT
GGAGACGTGGAGGAGAACCCTGGACCTCCCAAGAAAAAACGCAAGGTGGGTTCTAA
GCTACTGTCTTCTATCGAACAAGCATGCGATATTTGCCGACTTAAAAAGCTCAAGTGC
TCCAAAGAAAAACCGAAGTGCGCCAAGTGTCTGAAGAACAACTGGGAGTGTCGCTA
CTCTCCCAAAACCAAAAGGTCTCCGCTGACTAGGGCACATCTGACAGAAGTGGAATC
AAGGCTAGAAAGACTGGAACAGCTATTTCTACTGATTTTTCCTCGAGAAGACCTTGAC
ATGATTTTGAAAATGGATTCTTTACAGGATATAAAAGCATTGTTAACAGGATTATTTGTA
CAAGATAATGTGAATAAAGATGCCGTCACAGATAGATTGGCTTCAGTGGAGACTGATA
TGCCTCTAACATTGAGACAGCATAGAATAAGTGCGACATCATCATCGGAAGAGAGTAG
TAACAAAGGTCAAAGACAGTTGACTGTATCGGGATCTGACGCATTGGACGATTTTGAT
CTGGATATGCTGGGAAGTGACGCCCTCGATGATTTTGACCTTGACATGCTTGGTTCG
GATGCCCTTGATGACTTTGACCTCGACATGCTCGGCAGTGACGCCCTTGATGATTTC
GACCTGGACATGCTGGGTAGTCCTAAGAAAAAGCGGAAAGTGTAATCTAGAGATCCA
GACATGATAAGATACATTGATGAGTTTGGACAAACCACAACTAGAATGCAGTGAAAAA
AATGCTTTATTTGTGAAATTTGTGATGCTATTGCTTTATTTGTAACCATTATAAGCTGCA
ATAAACAAGTTAACAACAACAATTGCATTCATTTTATGTTTCAGGTTCAGGGGGAGGT

GTGGGAGGTTTTTTAATTCGCGGCCGCGAATTCACTAGTGATTGCTCGAGTGGCCAG
ATCCTGTGCCTTCTAGTTGCCAGCCATCTGTTGTTTGCCCCTCCCCCGTGCCTTCCT
TGACCCTGGAAGGTGCCACTCCCACTGTCCTTTCCTAATAAAATGAGGAAATTGCATC
GCATTGTCTGAGTAGGTGTCATTCTATTCTGGGGGGTGGGGTGGGGCAGGACAGCA
AGGGGGAGGATTGGGAAGACAATAGCAGGCATGCTGGGGATGCGGTGGGCTCTATG
GCACGTGCGCCGGTGCGGAACTTGTTTATTGCAGCTTATAATGGTTACAAATAAAGCA
ATAGCATCACAAATTTCACAAATAAAGCATTTTTTTCACTGCATTCTAGTTGTGGTTTGT
CCAAACTCATCAATGTATCTTATCATGTCTGGAAACTTGTTTATTGCAGCTTATAATGGT
TACAAATAAAGCAATAGCATCACAAATTTCACAAATAAAGCATTTTTTTCACTGCATTCT
AGTTGTGGTTTGTCCAAACTCATCAATGTATCTTATCATGTCCTGAAACTTGTTTATTG
CAGCTTATAATGGTTACAAATAAAGCAATAGCATCACAAATTTCACAAATAAAGCATTTT
TTTCACTGCATTCTAGTTGTGGTTTGTCCAAACTCATCAATGTATCTTATCATGTCTGG
ATCGGAGGACGACTCCCGGGCGGAGTACTGTCCTCCGAGCGGATTAGAAGCCACC
GGATCCGGGTGACAGCCCTCCGTCTTCACGGGATACTCTACACCGTAGGGTTCCGG
AGTACTGTCCTCCGCGGAGACTCTAGAGGGTATATAATGGATCCCATCGCGTCTCAG
CCTCACTTTGAGCTCCTCCACACGAATTCGTCGACATGGTGAGCAAGGGCGAGGAG
CTGTTCACCGGGGTGGTGCCCATCCTGGTCGAGCTGGACGGCGACGTAAACGGCC
ACAAGTTCAGCGTGTCCGGCGAGGGCGAGGGCGATGCCACCTACGGCAAGCTGAC
CCTGAAGTTCATCTGCACCACCGGCAAGCTGCCCGTGCCCTGGCCCACCCTCGTGA
CCACCCTGACCTACGGCGTGCAGTGCTTCAGCCGCTACCCCGACCACATGAAGCAG
CACGACTTCTTCAAGTCCGCCATGCCCGAAGGCTACGTCCAGGAGCGCACCATCTT
CTTCAAGGACGACGGCAACTACAAGACCCGCGCCGAGGTGAAGTTCGAGGGCGAC
ACCCTGGTGAACCGCATCGAGCTGAAGGGCATCGACTTCAAGGAGGACGGCAACAT
CCTGGGGCACAAGCTGGAGTACAACTACAACAGCCACAACGTCTATATCATGGCCGA
CAAGCAGAAGAACGGCATCAAGGTGAACTTCAAGATCCGCCACAACATCGAGGACG
GCAGCGTGCAGCTCGCCGACCACTACCAGCAGAACACCCCCATCGGCGACGGCCC
CGTGCTGCTGCCCGACAACCACTACCTGAGCACCCAGTCCGCCCTGAGCAAAGACC
CCAACGAGAAGCGCGATCACATGGTCCTGCTGGAGTTCGTGACCGCCGCCGGGAT
CACTCTCGGCATGGACGAGCTGTACAAGTAATCTAGAGATCCAGACATGATAAGATAC
ATTGATGAGTTTGGACAAACCACAACTAGAATGCAGTGAAAAAAATGCTTTATTTGTG
AAATTTGTGATGCTATTGCTTTATTTGTAACCATTATAAGCTGCAATAAACAAGTTAACA
ACAACAATTGCATTCATTTTATGTTTCAGGTTCAGGGGGAGGTGTGGGAGGTTTTTTA
ATTCGCGGCCGCGAATTCACTAGTGATTGCTCGAGTGGCCAGATCCTGTGCCTTCTA
GTTGCCAGCCATCTGTTGTTTGCCCCTCCCCCGTGCCTTCCTTGACCCTGGAAGGT
GCCACTCCCACTGTCCTTTCCTAATAAAATGAGGAAATTGCATCGCATTGTCTGAGTA
GGTGTCATTCTATTCTGGGGGGTGGGGTGGGGCAGGACAGCAAGGGGGAGGATTG
GGAAGACAATAGCAGGCATGCTGGGGATGCGGTGGGCTCTATGGCGAGGGACAGC
CCCCCCCCAAAGCCCCCAGGGATGTAATTACGTCCCTCCCCCGCTAGGGGGCAGCA
GCGAGCCGCCCGGGGCTCCGCTCCGGTCCGGCGCTCCCCCCGCATCCCCGAGCC
GGCAGCGTGCGGGGACAGCCCGGGCACGGGGAAGGTGGCACGGGATCGCTTTCC
TCTGAACGCTTCTCGCTGCTCTTTGAGCCTGCAGACACCTGGGGGGATACGGGGAA
AAGAGGGACAGCCCCCCCCCAAAGCCCCCAGGGATGTAATTACGTCCCTCCCCCGC
TAGGGGGCAGCAGCGAGCCGCCCGGGGCTCCGCTCCGGTCCGGCGCTCCCCCCG
CATCCCCGAGCCGGCAGCGTGCGGGGACAGCCCGGGCACGGGGAAGGTGGCACG

GGATCGCTTTCCTCTGAACGCTTCTCGCTGCTCTTTGAGCCTGCAGACACCTGGGG
GGATACGGGGAAAACGCGCCTAAAA

***cx43.4*-VH**

right arm-*lamGolden*-left arm-CDS-P2A-Gal4-VP64-HSF1-SV40 polyA-bGH polyA-3× SV40 polyA-5×UAS-E1b-eGFP-SV40 polyA-bGH polyA-2×insulator

GCGCAGGCACCTGAAACTGGCTCAGCCCGTGCAGGAAGTCGTTGACCCCGTCATC
GCGAGACATTGACCGCCTTCGTCGTCATTTAAAGTTAGCCCAACAGCACCTGGACCT
CGCCTATCAGAATGGCGAGAGCAGTCCTTCACGCAGCAGCAGCCCAGAGTCCAACG
GCACTGCTGTCGAGCAGAACAGACTTAACTTTGCTCAGGAGAAGCAGGGGAGCAAA
TGTGAAAAAGGATCCATGCTGGTACCGCTACTAACTTCAGCCTGCTGAAGCAGGCT
GGAGACGTGGAGGAGAACCCTGGACCTCCCAAGAAAAAACGCAAGGTGGGTTCTAA
GCTACTGTCTTCTATCGAACAAGCATGCGATATTTGCCGACTTAAAAAGCTCAAGTGC
TCCAAAGAAAAACCGAAGTGCGCCAAGTGTCTGAAGAACAACTGGGAGTGTCGCTA
CTCTCCCAAAACCAAAAGGTCTCCGCTGACTAGGGCACATCTGACAGAAGTGGAATC
AAGGCTAGAAAGACTGGAACAGCTATTTCTACTGATTTTTCCTCGAGAAGACCTTGAC
ATGATTTTGAAAATGGATTCTTTACAGGATATAAAAGCATTGTTAACAGGATTATTTGTA
CAAGATAATGTGAATAAAGATGCCGTCACAGATAGATTGGCTTCAGTGGAGACTGATA
TGCCTCTAACATTGAGACAGCATAGAATAAGTGCGACATCATCATCGGAAGAGAGTAG
TAACAAAGGTCAAAGACAGTTGACTGTATCGGGATCTGACGCATTGGACGATTTTGAT
CTGGATATGCTGGGAAGTGACGCCCTCGATGATTTTGACCTTGACATGCTTGGTTCG
GATGCCCTTGATGACTTTGACCTCGACATGCTCGGCAGTGACGCCCTTGATGATTTC
GACCTGGACATGCTGGGTAGTGGCTCAGGATCCGGCTTCAGCGTGGACACCAGTG
CCCTGCTGGACCTGTTCAGCCCCTCGGTGACCGTGCCCGACATGAGCCTGCCTGA
CCTTGACAGCAGCCTGGCCAGTATCCAAGAGCTCCTGTCTCCCCAGGAGCCCCCCA
GGCCTCCCGAGGCAGAGAACAGCAGCCCGGATTCAGGGAAGCAGCTGGTGCACTA
CACAGCGCAGCCGCTGTTCCTGCTGGACCCCGGCTCCGTGGACACCGGGAGCAAC
GACCTGCCGGTGCTGTTTGAGCTGGGAGAGGGCTCCTACTTCTCCGAAGGGGACG
GCTTCGCCGAGGACCCCACCATCTCCCTGCTGACAGGCTCGGAGCCTCCCAAAGC
CAAGGACCCCACTGTCTCCGGTAGCCCTAAGAAAAAGCGGAAAGTGTAATCTAGAGA
TCCAGACATGATAAGATACATTGATGAGTTTGGACAAACCACAACTAGAATGCAGTGA
AAAAAATGCTTTATTTGTGAAATTTGTGATGCTATTGCTTTATTTGTAACCATTATAAGCT
GCAATAAACAAGTTAACAACAACAATTGCATTCATTTTATGTTTCAGGTTCAGGGGGA
GGTGTGGGAGGTTTTTTAATTCGCGGCCGCGAATTCACTAGTGATTGCTCGAGTGGC
CAGATCCTGTGCCTTCTAGTTGCCAGCCATCTGTTGTTTGCCCCTCCCCCGTGCCTT
CCTTGACCCTGGAAGGTGCCACTCCCACTGTCCTTTCCTAATAAAATGAGGAAATTG
CATCGCATTGTCTGAGTAGGTGTCATTCTATTCTGGGGGGTGGGGTGGGGCAGGAC
AGCAAGGGGGAGGATTGGGAAGACAATAGCAGGCATGCTGGGGATGCGGTGGGCT
CTATGGCACGTGCGCCGGTGCGGAACTTGTTTATTGCAGCTTATAATGGTTACAAATA
AAGCAATAGCATCACAAATTTCACAAATAAAGCATTTTTTTCACTGCATTCTAGTTGTG
GTTTGTCCAAACTCATCAATGTATCTTATCATGTCTGGAAACTTGTTTATTGCAGCTTAT
AATGGTTACAAATAAAGCAATAGCATCACAAATTTCACAAATAAAGCATTTTTTTCACTG
CATTCTAGTTGTGGTTTGTCCAAACTCATCAATGTATCTTATCATGTCCTGAAACTTGT
TTATTGCAGCTTATAATGGTTACAAATAAAGCAATAGCATCACAAATTTCACAAATAAAG

CATTTTTTTCACTGCATTCTAGTTGTGGTTTGTCCAAACTCATCAATGTATCTTATCATG
TCTGGATCGGAGGACGACTCCCGGGCGGAGTACTGTCCTCCGAGCGGATTAGAAGC
CACCGGATCCGGGTGACAGCCCTCCGTCTTCACGGGATACTCTACACCGTAGGGTT
CCGGAGTACTGTCCTCCGCGGAGACTCTAGAGGGTATATAATGGATCCCATCGCGTC
TCAGCCTCACTTTGAGCTCCTCCACACGAATTCGTCGACATGGTGAGCAAGGGCGA
GGAGCTGTTCACCGGGGTGGTGCCCATCCTGGTCGAGCTGGACGGCGACGTAAAC
GGCCACAAGTTCAGCGTGTCCGGCGAGGGCGAGGGCGATGCCACCTACGGCAAGC
TGACCCTGAAGTTCATCTGCACCACCGGCAAGCTGCCCGTGCCCTGGCCCACCCTC
GTGACCACCCTGACCTACGGCGTGCAGTGCTTCAGCCGCTACCCCGACCACATGAA
GCAGCACGACTTCTTCAAGTCCGCCATGCCCGAAGGCTACGTCCAGGAGCGCACCA
TCTTCTTCAAGGACGACGGCAACTACAAGACCCGCGCCGAGGTGAAGTTCGAGGGC
GACACCCTGGTGAACCGCATCGAGCTGAAGGGCATCGACTTCAAGGAGGACGGCA
ACATCCTGGGGCACAAGCTGGAGTACAACTACAACAGCCACAACGTCTATATCATGG
CCGACAAGCAGAAGAACGGCATCAAGGTGAACTTCAAGATCCGCCACAACATCGAG
GACGGCAGCGTGCAGCTCGCCGACCACTACCAGCAGAACACCCCCATCGGCGACG
GCCCCGTGCTGCTGCCCGACAACCACTACCTGAGCACCCAGTCCGCCCTGAGCAA
AGACCCCAACGAGAAGCGCGATCACATGGTCCTGCTGGAGTTCGTGACCGCCGCC
GGGATCACTCTCGGCATGGACGAGCTGTACAAGTAATCTAGAGATCCAGACATGATA
AGATACATTGATGAGTTTGGACAAACCACAACTAGAATGCAGTGAAAAAAATGCTTTAT
TTGTGAAATTTGTGATGCTATTGCTTTATTTGTAACCATTATAAGCTGCAATAAACAAGT
TAACAACAACAATTGCATTCATTTTATGTTTCAGGTTCAGGGGGAGGTGTGGGAGGTT
TTTTAATTCGCGGCCGCGAATTCACTAGTGATTGCTCGAGTGGCCAGATCCTGTGCC
TTCTAGTTGCCAGCCATCTGTTGTTTGCCCCTCCCCCGTGCCTTCCTTGACCCTGGA
AGGTGCCACTCCCACTGTCCTTTCCTAATAAAATGAGGAAATTGCATCGCATTGTCTG
AGTAGGTGTCATTCTATTCTGGGGGGTGGGGTGGGGCAGGACAGCAAGGGGGAGG
ATTGGGAAGACAATAGCAGGCATGCTGGGGATGCGGTGGGCTCTATGGCGAGGGAC
AGCCCCCCCCCAAAGCCCCCAGGGATGTAATTACGTCCCTCCCCCGCTAGGGGGCA
GCAGCGAGCCGCCCGGGGCTCCGCTCCGGTCCGGCGCTCCCCCCGCATCCCCGA
GCCGGCAGCGTGCGGGGACAGCCCGGGCACGGGGAAGGTGGCACGGGATCGCTT
TCCTCTGAACGCTTCTCGCTGCTCTTTGAGCCTGCAGACACCTGGGGGGATACGGG
GAAAAGAGGGACAGCCCCCCCCCAAAGCCCCCAGGGATGTAATTACGTCCCTCCCC
CGCTAGGGGGCAGCAGCGAGCCGCCCGGGGCTCCGCTCCGGTCCGGCGCTCCCC
CCGCATCCCCGAGCCGGCAGCGTGCGGGGACAGCCCGGGCACGGGGAAGGTGGC
ACGGGATCGCTTTCCTCTGAACGCTTCTCGCTGCTCTTTGAGCCTGCAGACACCTG
GGGGGATACGGGGAAAACGCGCCTAAAA

***cx43.4*-SPH**

right arm-*lamGolden*-left arm-CDS-P2A-Gal4-GCN4-SV40 polyA-bGH polyA-CMV-scFV GCN4-P65-HSF1-SV40 polyA-bGH polyA-3×SV40 polyA-5×UAS-E1b-eGFP-SV40 polyA-bGH polyA-2×insulator

GCGCAGGCACCTGAAACTGGCTCAGCCCGTGCAGGAAGTCGTTGACCCCGTCATC
GCGAGACATTGACCGCCTTCGTCGTCATTTAAAGTTAGCCCAACAGCACCTGGACCT
CGCCTATCAGAATGGCGAGAGCAGTCCTTCACGCAGCAGCAGCCCAGAGTCCAACG
GCACTGCTGTCGAGCAGAACAGACTTAACTTTGCTCAGGAGAAGCAGGGGAGCAAA

TGTGAAAAAGGGATCCATGCTGGTACCGCTACTAACTTCAGCCTGCTGAAGCAGGCT
GGAGACGTGGAGGAGAACCCTGGACCTAAGCTACTGTCTTCTATCGAACAAGCATG
CGATATTTGCCGACTTAAAAAGCTCAAGTGCTCCAAAGAAAAACCGAAGTGCGCCAA
GTGTCTGAAGAACAACTGGGAGTGTCGCTACTCTCCCAAAACCAAAAGGTCTCCGC
TGACTAGGGCACATCTGACAGAAGTGGAATCAAGGCTAGAAAGACTGGAACAGCTAT
TTCTACTGATTTTTCCTCGAGAAGACCTTGACATGATTTTGAAAATGGATTCTTTACAG
GATATAAAAGCATTGTTAACAGGATTATTTGTACAAGATAATGTGAATAAAGATGCCGTC
ACAGATAGATTGGCTTCAGTGGAGACTGATATGCCTCTAACATTGAGACAGCATAGAA
TAAGTGCGACATCATCATCGGAAGAGAGTAGTAACAAAGGTCAAAGACAGTTGACTG
TATCGGGATCTTATCCCTATGACGTGCCCGATTATGCCAGCCTGGGCAGCGGCTCCC
CCAAGAAAAAACGCAAGGTGGAAGATCCTAAGAAAAAGCGGAAAGTGGACGGCATT
GGTAGTGGGAGCAACGGCAGCAGCGGATCCaaCGGTCCGactgacgCCGCGGaaGAA
GAACTTTTGAGCAAGAATTATCATCTTGAGAACGAAGTGGCTCGTCTTAAGAAAGGTT
CTGGCAGTGGAGAAGAACTGCTTTCAAAGAATTACCACCTGGAAAATGAGGTAGCTA
GACTGAAAAAGGGGAGCGGAAGTGGGGAGGAGTTGCTGAGCAAAAATTATCATTTG
GAGAACGAAGTAGCACGACTAAAGAAAGGGTCCGGATCGGGTGAGGAGTTACTCTC
GAAAAATTATCATCTCGAAAACGAAGTGGCTCGGCTAAAAAAGGGCAGTGGTTCTGG
AGAAGAGCTATTATCTAAAAACTACCACCTCGAAAATGAGGTGGCACGCTTAAAAAAG
GGAAGTGGCAGTGGTGAAGAGCTACTATCCAAGAATTATCATCTTGAGAACGAGGTA
GCGCGTTTGAAGAAGGGTTCCGGCTCAGGAGAGGAACTGCTCTCGAAGAACTATCA
TCTTGAAAATGAGGTCGCTCGATTAAAAAAGGGATCGGGCAGTGGTGAGGAACTACT
TTCAAAGAATTACCACCTCGAAAACGAAGTAGCTCGATTAAAGAAAGGTTCAGGGTC
GGGTGAAGAATTACTGAGTAAAAATTATCATCTGGAAAATGAGGTAGCGAGACTAAAA
AAGGGGAGTGGTTCTGGCGAGGAATTGCTATCGAAAAATTATCATCTTGAGAACGAA
GTTGCTAGGCTCAAAAAGGGCTCAGGCTCAGGCACCGCGGtaaACATAGGTGGTGG
AACCGGTCCGatggatctacagcggccgcaaGGTGGAGGTGGACCCAAGAAGAAGCGCAAG
GTGTAATCTAGAGATCCAGACATGATAAGATACATTGATGAGTTTGGACAAACCACAA
CTAGAATGCAGTGAAAAAAATGCTTTATTTGTGAAATTTGTGATGCTATTGCTTTATTTG
TAACCATTATAAGCTGCAATAAACAAGTTAACAACAACAATTGCATTCATTTTATGTTTC
AGGTTCAGGGGGAGGTGTGGGAGGTTTTTTAATTCGCGGCCGCGAATTCACTAGTG
ATTGCTCGAGTGGCCAGATCCTGTGCCTTCTAGTTGCCAGCCATCTGTTGTTTGCCC
CTCCCCCGTGCCTTCCTTGACCCTGGAAGGTGCCACTCCCACTGTCCTTTCCTAATA
AAATGAGGAAATTGCATCGCATTGTCTGAGTAGGTGTCATTCTATTCTGGGGGGTGG
GGTGGGGCAGGACAGCAAGGGGGAGGATTGGGAAGACAATAGCAGGCATGCTGGG
GATGCGGTGGGCTCTATGGTCATGAGACATTGATTATTGACTAGTTATTAATAGTAATC
AATTACGGGGTCATTAGTTCATAGCCCATATATGGAGTTCCGCGTTACATAACTTACGG
TAAATGGCCCGCCTGGCTGACCGCCCAACGACCCCCGCCCATTGACGTCAATAATG
ACGTATGTTCCCATAGTAACGCCAATAGGGACTTTCCATTGACGTCAATGGGTGGACT
ATTTACGGTAAACTGCCCACTTGGCAGTACATCAAGTGTATCATATGCCAAGTACGCC
CCCTATTGACGTCAATGACGGTAAATGGCCCGCCTGGCATTATGCCCAGTACATGAC
CTTATGGGACTTTCCTACTTGGCAGTACATCTACGTATTAGTCATCGCTATTACCATGG
TGATGCGGTTTTGGCAGTACATCAATGGGCGTGGATAGCGGTTTGACTCACGGGGAT
TTCCAAGTCTCCACCCCATTGACGTCAATGGGAGTTTGTTTTGGCACCAAAATCAAC
GGGACTTTCCAAAATGTCGTAACAACTCCGCCCCATTGACGCAAATGGGCGGTAGG

CGTGTACGGTGGGAGGTCTATATAAGCAGCGCGTTTTGCCTGTACTGGGTCTCTCTG
GTcccaagcttGCCACCATGCCTAAGAAGAAAAGAAAGGTGGGAAGCATGGGCCCCGAC
ATCGTGATGACCCAGAGCCCCAGCAGCCTGAGCGCCAGCGTGGGCGACCGCGTGA
CCATCACCTGCCGCAGCAGCACCGGCGCCGTGACCACCAGCAACTACGCCAGCTG
GGTGCAGGAGAAGCCCGGCAAGCTGTTCAAGGGCCTGATCGGCGGCACCAACAAC
CGCGCCCCCGGCGTGCCCAGCCGCTTCAGCGGCAGCCTGATCGGCGACAAGGCC
ACCCTGACCATCAGCAGCCTGCAGCCCGAGGACTTCGCCACCTACTTCTGCGCCCT
GTGGTACAGCAACCACTGGGTGTTCGGCCAGGGCACCAAGGTGGAGCTGAAGCGC
GGCGGCGGCGGCAGCGGCGGCGGCGGCAGCGGCGGCGGCGGCAGCAGCGGCG
GCGGCAGCGAGGTGAAGCTGCTGGAGAGCGGCGGCGGCCTGGTGCAGCCCGGC
GGCAGCCTGAAGCTGAGCTGCGCCGTGAGCGGCTTCAGCCTGACCGACTACGGCG
TGAACTGGGTGCGCCAGGCCCCCGGCCGCGGCCTGGAGTGGATCGGCGTGATCTG
GGGCGACGGCATCACCGACTACAACAGCGCCCTGAAGGACCGCTTCATCATCAGCA
AGGACAACGGCAAGAACACCGTGTACCTGCAGATGAGCAAGGTGCGCAGCGACGA
CACCGCCCTGTACTACTGCGTGACCGGCCTGTTCGACTACTGGGGCCAGGGCACC
CTGGTGACCGTGAGCAGCTACCCATACGATGTTCCAGATTACGCTGGTGGAGGCGG
AGGTTCTGGGGGAGGAGGTAGTGGCGGTGGTGGTTCAGGAGGCGGCGGAAGCccta
agaaaaagaggaaggtgGCGGCCGCTGGATCCCCTTCAGGGCAGATCAGCAACCAGGCC
CTGGCTCTGGCCCCTAGCTCCGCTCCAGTGCTGGCCCAGACTATGGTGCCCTCTAG
TGCTATGGTGCCTCTGGCCCAGCCACCTGCTCCAGCCCCTGTGCTGACCCCAGGAC
CACCCCAGTCACTGAGCGCTCCAGTGCCCAAGTCTACACAGGCCGGCGAGGGGAC
TCTGAGTGAAGCTCTGCTGCACCTGCAGTTCGACGCTGATGAGGACCTGGGAGCTC
TGCTGGGGAACAGCACCGATCCCGGAGTGTTCACAGATCTGGCCTCCGTGGACAAC
TCTGAGTTTCAGCAGCTGCTGAATCAGGGCGTGTCCATGTCTCATAGTACAGCCGAA
CCAATGCTGATGGAGTACCCCGAAGCCATTACCCGGCTGGTGACCGGCAGCCAGCG
GCCCCCCGACCCCGCTCCAACTCCCCTGGGAACCAGCGGCCTGCCTAATGGGCTG
TCCGGAGATGAAGACTTCTCAAGCATCGCTGATATGGACTTTAGTGCCCTGCTGTCA
CAGATTTCCTCTAGTGGGCAGGGAGGAGGTGGAAGCGGCTTCAGCGTGGACACCA
GTGCCCTGCTGGACCTGTTCAGCCCCTCGGTGACCGTGCCCGACATGAGCCTGCC
TGACCTTGACAGCAGCCTGGCCAGTATCCAAGAGCTCCTGTCTCCCCAGGAGCCCC
CCAGGCCTCCCGAGGCAGAGAACAGCAGCCCGGATTCAGGGAAGCAGCTGGTGCA
CTACACAGCGCAGCCGCTGTTCCTGCTGGACCCCGGCTCCGTGGACACCGGGAGC
AACGACCTGCCGGTGCTGTTTGAGCTGGGAGAGGGCTCCTACTTCTCCGAAGGGG
ACGGCTTCGCCGAGGACCCCACCATCTCCCTGCTGACAGGCTCGGAGCCTCCCAA
AGCCAAGGACCCCACTGTCTCCGCTAGCGAGGGCAGAGGAAGTCTGCTAACATGCC
CTAAGAAGAAGCGCAAGGTGTAATCTAGAGATCCAGACATGATAAGATACATTGATGA
GTTTGGACAAACCACAACTAGAATGCAGTGAAAAAAATGCTTTATTTGTGAAATTTGT
GATGCTATTGCTTTATTTGTAACCATTATAAGCTGCAATAAACAAGTTAACAACAACAAT
TGCATTCATTTTATGTTTCAGGTTCAGGGGGAGGTGTGGGAGGTTTTTTAATTCGCGG
CCGCGAATTCACTAGTGATTGCTCGAGTGGCCAGATCCTGTGCCTTCTAGTTGCCAG
CCATCTGTTGTTTGCCCCTCCCCCGTGCCTTCCTTGACCCTGGAAGGTGCCACTCC
CACTGTCCTTTCCTAATAAAATGAGGAAATTGCATCGCATTGTCTGAGTAGGTGTCATT
CTATTCTGGGGGGTGGGGTGGGGCAGGACAGCAAGGGGGAGGATTGGGAAGACAA
TAGCAGGCATGCTGGGGATGCGGTGGGCTCTATGGCACGTGCGCCGGTGCGGAAC

TTGTTTATTGCAGCTTATAATGGTTACAAATAAAGCAATAGCATCACAAATTTCACAAAT
AAAGCATTTTTTTCACTGCATTCTAGTTGTGGTTTGTCCAAACTCATCAATGTATCTTAT
CATGTCTGGAAACTTGTTTATTGCAGCTTATAATGGTTACAAATAAAGCAATAGCATCA
CAAATTTCACAAATAAAGCATTTTTTTCACTGCATTCTAGTTGTGGTTTGTCCAAACTC
ATCAATGTATCTTATCATGTCCTGAAACTTGTTTATTGCAGCTTATAATGGTTACAAATAA
AGCAATAGCATCACAAATTTCACAAATAAAGCATTTTTTTCACTGCATTCTAGTTGTGG
TTTGTCCAAACTCATCAATGTATCTTATCATGTCTGGATCGGAGGACGACTCCCGGGC
GGAGTACTGTCCTCCGAGCGGATTAGAAGCCACCGGATCCGGGTGACAGCCCTCCG
TCTTCACGGGATACTCTACACCGTAGGGTTCCGGAGTACTGTCCTCCGCGGAGACTC
TAGAGGGTATATAATGGATCCCATCGCGTCTCAGCCTCACTTTGAGCTCCTCCACACG
AATTCGTCGACATGGTGAGCAAGGGCGAGGAGCTGTTCACCGGGGTGGTGCCCAT
CCTGGTCGAGCTGGACGGCGACGTAAACGGCCACAAGTTCAGCGTGTCCGGCGAG
GGCGAGGGCGATGCCACCTACGGCAAGCTGACCCTGAAGTTCATCTGCACCACCG
GCAAGCTGCCCGTGCCCTGGCCCACCCTCGTGACCACCCTGACCTACGGCGTGCA
GTGCTTCAGCCGCTACCCCGACCACATGAAGCAGCACGACTTCTTCAAGTCCGCCA
TGCCCGAAGGCTACGTCCAGGAGCGCACCATCTTCTTCAAGGACGACGGCAACTAC
AAGACCCGCGCCGAGGTGAAGTTCGAGGGCGACACCCTGGTGAACCGCATCGAGC
TGAAGGGCATCGACTTCAAGGAGGACGGCAACATCCTGGGGCACAAGCTGGAGTAC
AACTACAACAGCCACAACGTCTATATCATGGCCGACAAGCAGAAGAACGGCATCAAG
GTGAACTTCAAGATCCGCCACAACATCGAGGACGGCAGCGTGCAGCTCGCCGACCA
CTACCAGCAGAACACCCCCATCGGCGACGGCCCCGTGCTGCTGCCCGACAACCAC
TACCTGAGCACCCAGTCCGCCCTGAGCAAAGACCCCAACGAGAAGCGCGATCACAT
GGTCCTGCTGGAGTTCGTGACCGCCGCCGGGATCACTCTCGGCATGGACGAGCTG
TACAAGTAATCTAGAGATCCAGACATGATAAGATACATTGATGAGTTTGGACAAACCAC
AACTAGAATGCAGTGAAAAAAATGCTTTATTTGTGAAATTTGTGATGCTATTGCTTTATT
TGTAACCATTATAAGCTGCAATAAACAAGTTAACAACAACAATTGCATTCATTTTATGTT
TCAGGTTCAGGGGGAGGTGTGGGAGGTTTTTTAATTCGCGGCCGCGAATTCACTAG
TGATTGCTCGAGTGGCCAGATCCTGTGCCTTCTAGTTGCCAGCCATCTGTTGTTTGC
CCCTCCCCCGTGCCTTCCTTGACCCTGGAAGGTGCCACTCCCACTGTCCTTTCCTAA
TAAAATGAGGAAATTGCATCGCATTGTCTGAGTAGGTGTCATTCTATTCTGGGGGGTG
GGGTGGGGCAGGACAGCAAGGGGGAGGATTGGGAAGACAATAGCAGGCATGCTGG
GGATGCGGTGGGCTCTATGGCGAGGGACAGCCCCCCCCCAAAGCCCCCAGGGATG
TAATTACGTCCCTCCCCCGCTAGGGGGCAGCAGCGAGCCGCCCGGGGCTCCGCTC
CGGTCCGGCGCTCCCCCCGCATCCCCGAGCCGGCAGCGTGCGGGGACAGCCCGG
GCACGGGGAAGGTGGCACGGGATCGCTTTCCTCTGAACGCTTCTCGCTGCTCTTTG
AGCCTGCAGACACCTGGGGGGATACGGGGAAAAGAGGGACAGCCCCCCCCCAAAG
CCCCCAGGGATGTAATTACGTCCCTCCCCCGCTAGGGGGCAGCAGCGAGCCGCCC
GGGGCTCCGCTCCGGTCCGGCGCTCCCCCCGCATCCCCGAGCCGGCAGCGTGCG
GGGACAGCCCGGGCACGGGGAAGGTGGCACGGGATCGCTTTCCTCTGAACGCTTC
TCGCTGCTCTTTGAGCCTGCAGACACCTGGGGGGATACGGGGAAAACGCGCCTAAA
A

mini promotor
miniCMV

GGTAGGCGTGTACGGTGGGAGGCCTATATAAGCAGAGCTCGTTTAGTGAACCGTCA
GATCGC

E1b

CATCGCGTCTCAGCCTCACTTT

TATA

ACGCCAGCTGGCGAAAGGGGGTATATAAGCAAGGCGATTAAGTTGGGTAACGCCAG
GGTTTTCC

random

ACGCCAGCTGGCGAAAGGGGGATCTGCTGCAAGGCGATTAAGTTGGGTAACGCCAG
GGTTTTCC
