# Supplementary material for: Optimal tagging strategies for illuminating expression profiles of genes with different abundance in zebrafish
Source: Commun Biol. 2023 Dec 21;6:1300. doi: 10.1038/s42003-023-05686-1 (PMC10739737; doi:10.1038/s42003-023-05686-1)
Supplement: Supplementary file 10 — Reporting Summary [file 42003_2023_5686_MOESM10_ESM.pdf]

nature portfolio

Corresponding author(s): Jiahuai Han and Yingying Zhang

Last updated by author(s): Nov 28, 2023

Reporting Summary

Nature Portfolio wishes to improve the reproducibility of the work that we publish. This form provides structure for consistency and transparency in reporting. For further information on Nature Portfolio policies, see our Editorial Policies and the Editorial Policy Checklist.

Statistics

For all statistical analyses, confirm that the following items are present in the figure legend, table legend, main text, or Methods section.

n/a	Confirmed	
☐	☒	The exact sample size (*n*) for each experimental group/condition, given as a discrete number and unit of measurement
☐	☒	A statement on whether measurements were taken from distinct samples or whether the same sample was measured repeatedly
☐	☒	The statistical test(s) used AND whether they are one- or two-sided<br>*Only common tests should be described solely by name; describe more complex techniques in the Methods section.*
☒	☐	A description of all covariates tested
☒	☐	A description of any assumptions or corrections, such as tests of normality and adjustment for multiple comparisons
☐	☒	A full description of the statistical parameters including central tendency (e.g. means) or other basic estimates (e.g. regression coefficient) AND variation (e.g. standard deviation) or associated estimates of uncertainty (e.g. confidence intervals)
☐	☒	For null hypothesis testing, the test statistic (e.g. *F*, *t*, *r*) with confidence intervals, effect sizes, degrees of freedom and *P* value noted<br>*Give P values as exact values whenever suitable.*
☒	☐	For Bayesian analysis, information on the choice of priors and Markov chain Monte Carlo settings
☒	☐	For hierarchical and complex designs, identification of the appropriate level for tests and full reporting of outcomes
☒	☐	Estimates of effect sizes (e.g. Cohen's *d*, Pearson's *r*), indicating how they were calculated

Our web collection on statistics for biologists contains articles on many of the points above.

Software and code

Policy information about availability of computer code

Data collection	1. RT-qPCR was performed using the Roche LightCycler® 96 Real-time system.<br>2. Images were taken using the SZX16 stereomicroscope (Olympus) and the Axio Observer 7 fluorescence microscope (Zeiss).
Data analysis	1. RT-qPCR data were analyzed using the LightCycler software.<br>2. Cleavage efficiency of sgRNA was analyzed using the TIDE Web tool (https://tide.nki.nl/).<br>3. Mean fluorescence intensity was calculated using the Cellsens software (Olympus).<br>4. Zebrafish images were processed using the ZEN 2.5 pro imaging software.<br>5. Statistical analyses were performed using the GraphPad Prism 8.0.1 software.

For manuscripts utilizing custom algorithms or software that are central to the research but not yet described in published literature, software must be made available to editors and reviewers. We strongly encourage code deposition in a community repository (e.g. GitHub). See the Nature Portfolio guidelines for submitting code & software for further information.

Data

Policy information about availability of data

All manuscripts must include a data availability statement. This statement should provide the following information, where applicable:

- Accession codes, unique identifiers, or web links for publicly available datasets
- A description of any restrictions on data availability
- For clinical datasets or third party data, please ensure that the statement adheres to our policy

All data supporting the findings of this study are available within the article and Supplementary information files, and also are available from the corresponding author upon reasonable request.

Research involving human participants, their data, or biological material

Policy information about studies with human participants or human data. See also policy information about sex, gender (identity/presentation), and sexual orientation and race, ethnicity and racism.

Reporting on sex and gender	N/A.
Reporting on race, ethnicity, or other socially relevant groupings	N/A.
Population characteristics	N/A.
Recruitment	N/A.
Ethics oversight	N/A.

Note that full information on the approval of the study protocol must also be provided in the manuscript.

Field-specific reporting

Please select the one below that is the best fit for your research. If you are not sure, read the appropriate sections before making your selection.

☒ Life sciences ☐ Behavioural & social sciences ☐ Ecological, evolutionary & environmental sciences

For a reference copy of the document with all sections, see nature.com/documents/nr-reporting-summary-flat.pdf

Life sciences study design

All studies must disclose on these points even when the disclosure is negative.

Sample size	Sample size and statistical analysis were included in appropriate places in the manuscript.
Data exclusions	No data were excluded.
Replication	Experimental replicates were indicated in the figure legends or Materials & Methods. All replication attempts were successful.
Randomization	Zebrafish inbreeding was performed by random selection of mating pairs. For microinjection, the allocation of zebrafish embryos into control or editing group was done randomly.
Blinding	Blinding was not necessary in our quantitative experiments.

Reporting for specific materials, systems and methods

We require information from authors about some types of materials, experimental systems and methods used in many studies. Here, indicate whether each material, system or method listed is relevant to your study. If you are not sure if a list item applies to your research, read the appropriate section before selecting a response.

Materials & experimental systems

n/a	Involved in the study
☒	☐ Antibodies
☒	☐ Eukaryotic cell lines
☒	☐ Palaeontology and archaeology
☐	☒ Animals and other organisms
☒	☐ Clinical data
☒	☐ Dual use research of concern
☒	☐ Plants

Methods

n/a	Involved in the study
☒	☐ ChIP-seq
☒	☐ Flow cytometry
☒	☐ MRI-based neuroimaging

Animals and other research organisms

Policy information about studies involving animals; ARRIVE guidelines recommended for reporting animal research, and Sex and Gender in Research

Laboratory animals	The parental zebrafish strain used in this research was wild-type Tübingen (Tü). Selection of mating pairs was random from a pool of 24 males and 24 females.
Wild animals	The study did not involve wild animals.
Reporting on sex	This finding was irrelevant to gender.
Field-collected samples	The study did not involve samples collected from the field.
Ethics oversight	All zebrafish husbandry and experiments were reviewed and approved by Laboratory Animal Management and Ethics Committee of Xiamen University and were in strict accordance with good animal practice as defined by Xiamen University Laboratory Animal Center.

Note that full information on the approval of the study protocol must also be provided in the manuscript.

Plants

Seed stocks	n/a
Novel plant genotypes	n/a
Authentication	n/a
